# A20 Modulates Lipid Metabolism and Energy Production to Promote Liver Regeneration

**DOI:** 10.1371/journal.pone.0017715

**Published:** 2011-03-17

**Authors:** Scott M. Damrauer, Peter Studer, Cleide G. da Silva, Christopher R. Longo, Haley E. Ramsey, Eva Csizmadia, Gautam V. Shrikhande, Salvatore T. Scali, Towia A. Libermann, Manoj K. Bhasin, Christiane Ferran

**Affiliations:** 1 Division of Vascular Surgery, Center for Vascular Biology Research and the Transplant Institute, Department of Surgery, Beth Israel Deaconess Medical Center, Harvard Medical School, Boston, Massachusetts, United States of America; 2 Division of Interdisciplinary Medicine and Biotechnology, Department of Medicine, Beth Israel Deaconess Medical Center, Harvard Medical School, Boston, Massachusetts, United States of America; Keio University, Japan

## Abstract

**Background:**

Liver Regeneration is clinically of major importance in the setting of liver injury, resection or transplantation. We have demonstrated that the NF-κB inhibitory protein A20 significantly improves recovery of liver function and mass following extended liver resection (LR) in mice. In this study, we explored the Systems Biology modulated by A20 following extended LR in mice.

**Methodology and Principal Findings:**

We performed transcriptional profiling using Affymetrix-Mouse 430.2 arrays on liver mRNA retrieved from recombinant adenovirus A20 (rAd.A20) and rAd.βgalactosidase treated livers, before and 24 hours after 78% LR. A20 overexpression impacted 1595 genes that were enriched for biological processes related to inflammatory and immune responses, **c**ellular proliferation, energy production, oxidoreductase activity, and lipid and fatty acid metabolism. These pathways were modulated by A20 in a manner that favored decreased inflammation, heightened proliferation, and optimized metabolic control and energy production. Promoter analysis identified several transcriptional factors that implemented the effects of A20, including NF-κB, CEBPA, OCT-1, OCT-4 and EGR1. Interactive scale-free network analysis captured the key genes that delivered the specific functions of A20. Most of these genes were affected at basal level and after resection. We validated a number of A20's target genes by real-time PCR, including p21, the mitochondrial solute carriers SLC25a10 and SLC25a13, and the fatty acid metabolism regulator, peroxisome proliferator activated receptor alpha. This resulted in greater energy production in A20-expressing livers following LR, as demonstrated by increased enzymatic activity of cytochrome c oxidase, or mitochondrial complex IV.

**Conclusion:**

This Systems Biology-based analysis unravels novel mechanisms supporting the pro-regenerative function of A20 in the liver, by optimizing energy production through improved lipid/fatty acid metabolism, and down-regulated inflammation. These findings support pursuit of A20-based therapies to improve patients’ outcomes in the context of extreme liver injury and extensive LR for tumor treatment or donation.

## Introduction

Among the viscera the liver has the unique capacity to regenerate following liver resection (LR), or liver injury. The mechanisms involved in this highly orchestrated repair process have been extensively studied in humans undergoing LR, and following liver transplantation or liver donation; and in animal models of partial hepatectomy (PH) [1]. Both directed studies and transcriptome-based approaches have demonstrated that the early regenerative phase is marked by rapid hepatocyte proliferation that usually occurs at the expense of a partial impairment of the liver metabolic function [2], [3], [4].

Liver regeneration or more precisely its “compensatory” growth is a complex process that comprises three main stages: transition of the quiescent hepatocyte into the cell cycle (priming); progression beyond the restriction point into the G1 phase of the cycle; and regulation of liver mass by apoptosis of excess hepatocytes in order to restore optimal liver mass/body mass ratio. In optimal priming and cycling conditions, it is the concerted activation of a transcriptional network including NF-κB, STAT-3, AP-1, C/EBP and peroxisome proliferator activated receptor (PPARα) that enables the up-regulation of all genes necessary for the restoration of a normal liver mass and function [5], [6], [7]. More than 70 genes are associated with liver regeneration including immediate, early genes (c-fos, c-jun,junB, c-myc), regulators of apoptosis (bcl-xL, MAPK, JNK); cell cycle (Cyclins, CDK, CDKI), and inflammatory and oxidative response genes (SOCS3, SOD and HO-1) [2], [3], [8], [9]. The expression of immediate early and delayed genes in liver regeneration does not lead to DNA replication unless cells can progress through the cell cycle following stimulation with growth factors, including Epithelial Growth Factor (EGF), Transforming Growth Factor β (TGFβ) and Hepatocyte Growth Factor (HGF) [10].

In order to maintain life, the regenerating liver must still support, even if partially, the metabolic demands of the host and provide for some liver synthetic function. Accordingly, a minimal “healthy” remnant liver mass is required to achieve these tasks. Experimentally, a 66–70% LR is always well tolerated in mice and rats. However extended (78%) or radical (87–90%) LR have a 50% and 100%, mortality rate, in mice [11], [12]. Also, the usually safe 66% LR in healthy mice is plagued with significant lethality when performed on livers with underlying metabolic disease, such as in PPARα-/- and Leptin or Leptin-receptor deficient mice [13], [14]. Similar issues are encountered in patients, where it is widely appreciated that recovery of liver mass following hepatectomy requires a metabolic compromise between differentiated function and organ re-growth. Accordingly, in clinical settings, hepatic failure after resection is more common when the organ is diseased such as in livers suffering from severe steatosis, or in small for size liver grafts [15].

Our aim over the past few years was to unravel novel therapeutic targets that would promote liver regeneration and function, and hence improve outcome of marginal and small for size liver grafts, and allow for safer extended liver resection in patients with large hepatic tumors. We have identified the NF-κB dependent and NF-κB-inhibitory [16], [17], [18], ubiquitin-editing protein A20/tnfaip3 [19] as a critical cytoprotective gene in the liver. A20 is induced in hepatocytes in response to inflammatory insults or injury, including liver resection, as part of a negative regulatory feedback loop aimed at re-establishing homeostasis [11], [20], [21]. A20 knockout mice are born cachectic and die within 3 weeks of birth as a result of unfettered inflammation in several organs, including the liver [22], which indicates the importance of A20 in the physiologic anti-inflammatory response, particularly in hepatocytes. To complement this, we have shown that overexpression of A20 in the liver protects mice from toxic hepatitis induced by D-galactosamine/lipopolysaccharide [21], fulminant hepatic failure following extended (78%) or radical (87%) LR [11], and severe liver ischemia/reperfusion injury [23]. The survival advantage afforded by A20 in these models of extreme liver injury relates to A20's ability to limit inflammation by down-regulating NF-κB activity, to inhibit TNF-induced apoptosis [21], to accelerate hepatocyte proliferation by decreasing transcription of the Cyclin-Dependent Kinase Inhibitor (CDKI) p21 [11], [22], and to protect hepatocytes from oxidative stress by increasing PPARα expression [23]. Given the central role of A20 in liver regeneration and repair, we set to explore, using a transcriptome-based approach, the critical pathways and gene networks that are modulated by A20 following extended (78%) LR that best models extensive liver resection in patients.

## Results

### Transgene Expression in mouse livers prior to resection

Expression of A20 and the β-galactosidase (βgal) transgenes in the mouse liver was achieved by penile vein injection of 1x10^9^ pfu of rAd. in 100 µL of normal saline, which results in optimal expression 5 days after injection in 30% to 40% of hepatocytes, as demonstrated by either Xgal staining, as described [21], or quantitative real time PCR (qPCR) that was performed in all tested samples to check for expression of human A20 and E. Coli βgalactosidase transgenes (Fig. S1 A). All LR were accordingly performed 5 days following rAd. injections (Fig. S1 B).

### Response to resection dominates transgene effect in influencing the overall transcriptome

We performed an unsupervised analysis of the microarray data in order to determine the relationship between samples and duplicates, and the effect of A20 overexpression and resection on global transcript expression (Fig. S2). By principal component analysis, we demonstrated that samples separated on the basis of resection status along primary component (PC) 1, which accounted for 47% of the variation between samples. On the other hand, the transgene effect was plotted along PC2, and accounted for 26% of the variation. We also performed hierarchical clustering using a Pearson correlation distance metric (Fig. S2, inset dendrogram), and demonstrated that sample similarity was strongly based on resection status. Importantly, all biological replicates clustered together.

### A20 modulates qualitative and quantitative transcript expression both at the basal state and in response to liver resection

**Figure 1:**
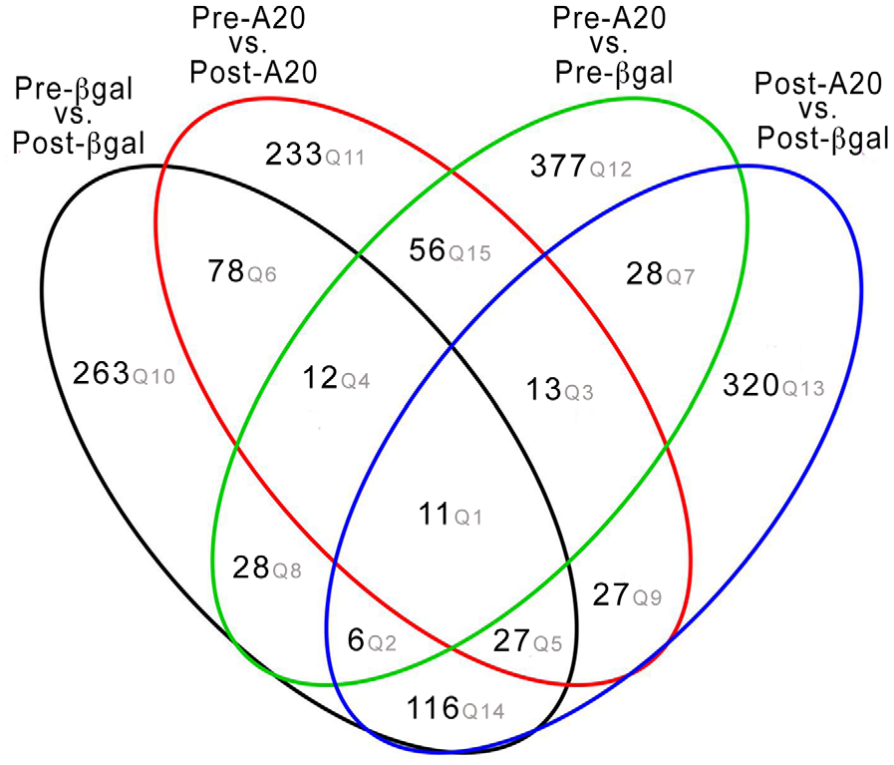
Venn diagram analysis on A20 modulated genes. Venn diagram showing the overlap among genes identified in any of the following comparisons: i) rAd.βgal after vs. before resection (β-gal post vs. pre), ii) rAd.A20 after vs. before resection (post-A20 vs. pre-A20), iii) rAd.A20 before resection vs. rAd.β gal before resection (pre-A20 vs. pre-βgal), or iv) rAd.A20 after resection vs. rAd. βgal after resection (post-A20 vs. post-βgal).

As stated in the methods, a given gene was considered differentially expressed, if 90% lower confidence bound (LCB) of the fold change (FC) between the two groups was above 1.5 [24]. LCB is a stringent estimate of FC and is considered to be a better ranking statistic [24], [25]. We identified a total of 1, 595 unique genes that met this criterion in at least one of the following comparisons: rAd.β gal after resection (post-βgal) vs. rAd.βgal before resection (pre-βgal) (541 genes), rAd.A20 after resection (post-A20) vs. rAd.A20 before resection (pre-A20) (457 genes), rAd.A20 before resection (pre-A20) vs. rAd.β-gal before resection (pre-βgal) (531 genes), or rAd.A20 after resection (post-A20) vs. rAd.β-gal after resection (post-βgal) (548 genes). Of the 1,595 genes, 402 were differentially expressed in more than one condition (Fig. 1; Table S2).

**Figure 2:**
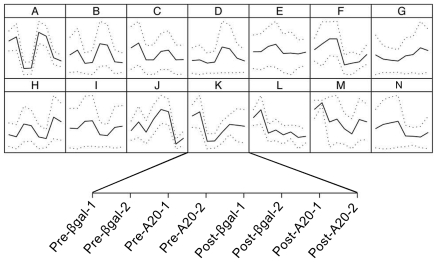
Transcriptional changes induced by A20 at the basal level and following liver resection. Self-organizing map (SOM) analysis representing the different expression patterns. Genes are selected using supervised analysis on the basis of LCB of the fold change (FC) in the following groups: “βgal before resection [pre-βgal-1 and pre-βgal-2]”, “βgal after resection [post-βgal-1 and post-βgal-2]”, “A20 before resection [pre-A20-1 and pre-A20-2]” and “A20 after resection [post-A20-1 and post-A20-2]. SOM clustering is performed on the 1,595 genes obtained after analysis of the genes that were differentially expressed among the four possible groups mentioned above (Informative genes analysis section in methods). To understand the biological theme of SOM patterns, Gene Ontology (GO) analysis was performed on each pattern, and identified a number of genes that regulated various interesting biological processes.

When using gene clustering and self-organizing maps (SOM) to detect groups of differentially expressed genes with similar expression patterns, we arbitrarily drew 100 separate maps according to Pearson correlation coefficient based distance metrics (data not shown). Evaluation of the maps showed significant similarity in the structure of many of the expression patterns, which allowed us to reduce the number of distinct expression profiles from 100 to 14 (Fig. 2). Gene expression patterns in which A20 altered the basal levels (group C), the final levels (group H), both basal and final levels (group I), basal levels but not the response to resection (group F), and the response to resection but not basal levels (group E), were identified (Fig. 2).

Analysis for functional enrichment by GO overrepresentation in co-expressed gene clusters demonstrated significant (Benjamini-Hochberg adjusted P-value 0.05) enrichment in 6 of the 14 clusters (Table S3). Most notably, we noted enrichment for acute-phase response (group A), immune and defense responses (group G), and oxidation/reduction genes (groups E and P).

### A20 affects liver processes involved in both normal function and recovery from injury

In order to better understand how A20 was affecting liver regeneration, we narrowed the list of differentially expressed genes to focus on those genes that were changing as a result of A20 overexpression, i.e. genes that met the following criteria: after resection LCB ≥ 1.5, or before resection LCB ≥ 1.5, or A20 LCB ≥ 1.5 and βgal LCB not ≥ 1.5. We assessed GO enrichment of the A20 interacting genes by using BiNGO, a Cytoscape plugin which maps significantly enriched GO terms onto a visual representation of the GO hierarchy [12], [26], [27]. Our results demonstrated a significant enrichment (Benjamini-Hochberg adjusted P-value < 0.05) in all 3 categories of GO terms: cellular component, molecular function, and biological process (Fig. S3). In particular, the enrichment analysis demonstrated that A20 significantly affected cell cycle, as well as immune, and regulatory processes that are important for liver proliferation in response to injury. A20 overexpression in the liver also enriched for oxidoreductase and electron carrier activity, as well as oxidation-reduction processes, which highly, suggests that A20 impacts the ability of the liver to produce energy and handle oxidative stress. A20 overexpressing liver were also enriched for genes involved in amino acid, fatty acid, steroid, and cholesterol biosynthesis, indicating that A20 primarily modulates the liver biosynthetic and metabolic processes.

**Figure 3:**
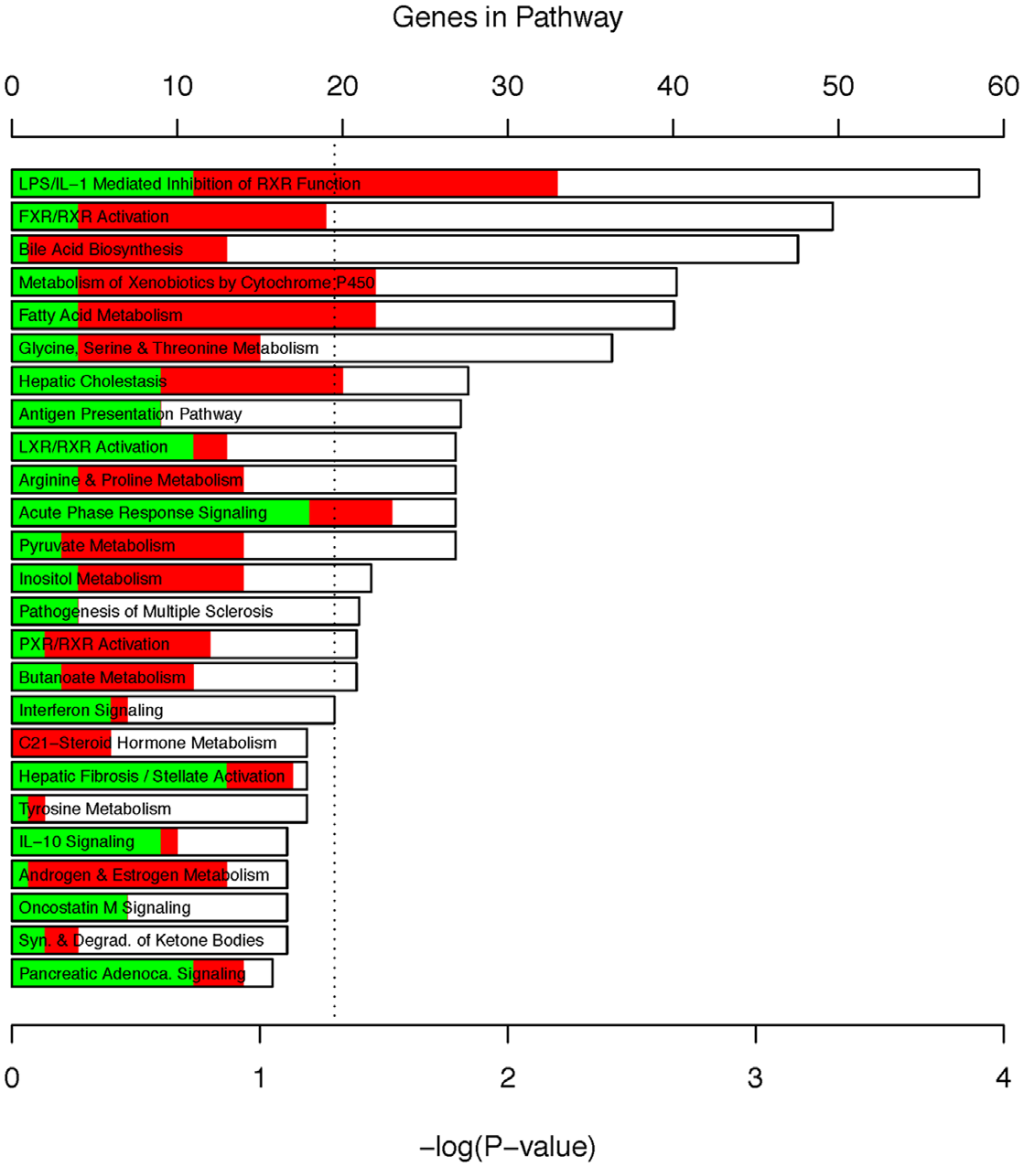
Analysis of pathways enriched by A20 overexpression. Each Bar represents a significantly enriched pathway as determined using the Benjamini-Hochberg hypothesis corrected P value (shown on primary X-axis). The directionality of the genes in each pathway is depicted using a pseudocolor (red for upregulated genes, green for downregulated genes and white for unmodified genes). The total numbers of genes in each pathway are shown on the secondary X-axis. The analysis for canonical pathways was performed using Ingenuity Systems.

Additionally, we probed for canonical pathways enrichment using the Ingenuity Pathway Analysis (IPA) tools and calculated the multiple test adjusted P-value for each pathway according to the fit of user's data to IPA database. This analysis confirmed a significant enrichment in genes involved in several biosynthetic and metabolic pathways, as well as acute phase response and inflammatory signaling (Fig. 3). In keeping with the well-described anti-inflammatory properties of A20, antigen presentation, interferon signaling, and acute phase response pathways showed an overall down-regulation of relevant transcripts. At the same time, the bulk of genes involved in metabolic pathways were upregulated by A20. Close examination of these metabolic pathways suggested that A20 overexpression enhanced the activity of these pathways, even after extended LR. These pathways being central to the metabolic function of the liver, this suggests that A20 overexpression not only enhances the liver's synthetic and metabolic functions, but also preserves these functions during the proliferative phase of liver regeneration, when they are usually down-regulated as a result of energy shunting.

### A20 impacts key nodes within inflammation, metabolism, energy production, and proliferation networks before and following extended liver resection

**Figure 4:**
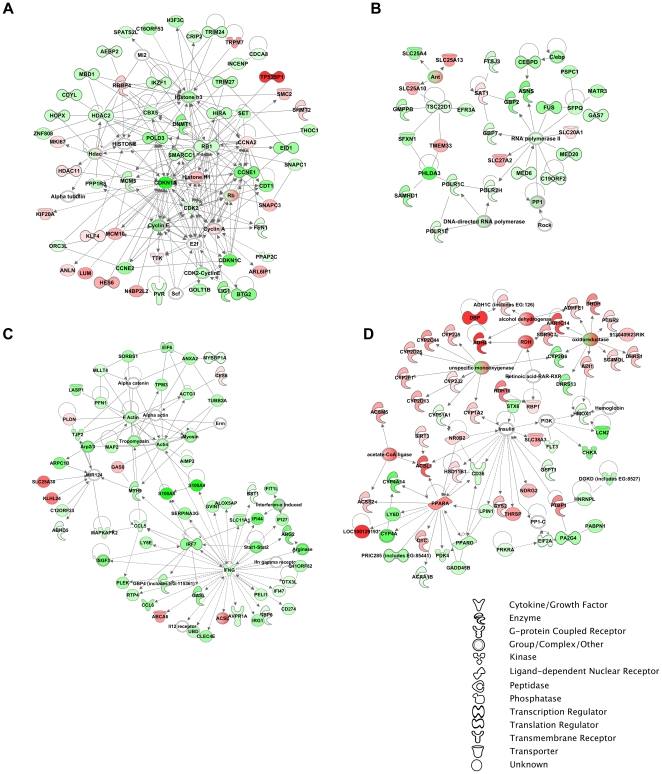
Network representation of the cellular functions supporting the effects of A20 overexpression. Networks shown: **A)** Cellular proliferation related genes with CDKN1A and CDK2 as primary regulatory nodes; **B)** Genes involved in energy production and small molecule biochemistry, such as SLC25A10 and SLC25A13; **C)** Inflammatory disease and immune response related genes with IFNγ as a critical regulatory node; and **D)** Genes involved in lipid and carbohydrate metabolism as well as oxidoreductase activity, with PPARα representing the dominant node. We used the Ingenuity Pathways Analysis tool (www.ingenuity.com) to generate the networks of genes influenced by A20 overexpression, and merged the major networks with obvious related functions. The intensity of the node color indicates the degree of up-regulation (red) and down-regulation (green), while white nodes indicate non-modified genes that may be affected in a non-transcriptional manner. All networks shown were significantly affected by A20 overexpression, with a score >10. The gene classes are represented by different symbols, as we depict in the attached legend.

Network analysis with IPA software was used to analyze the functional networks of the interacting genes and to identify the key nodes within these networks. Scale free networks were constructed based on the physical or regulatory interaction between genes or gene products. Our results showed a significantly enrichment of networks controlling cellular proliferation (Fig. 4A), energy production (Fig. 4B), inflammation (Fig. 4C), and metabolic functions (Fig. 4D), mirroring the result of the GO analysis. We merged the top 15 networks to create an overall system-wide network (Fig. S4). Key nodes were isolated based on edge density, and included the following molecules: CDKN1A (p21), RB1, CDK2, TGFBR1, CyclinA, PPARα, AP1, and IFNγ. While the individual fold changes of these molecules were not necessarily always significant, their centrality within the networks indicated that they were key modulators of A20's effects on liver regeneration.

### A limited, set of promoters is enriched in A20-expressing livers following extended resection

**Figure 5:**
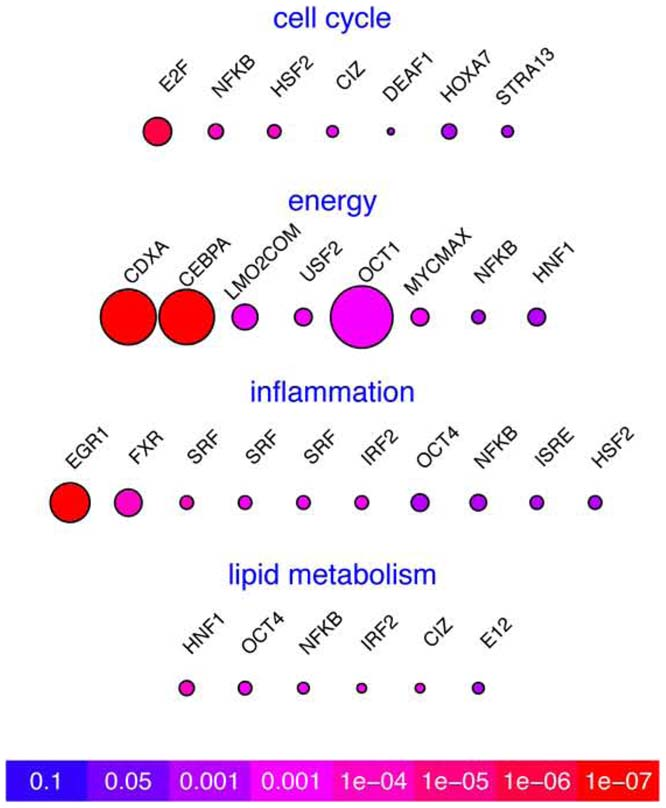
Regulatory analysis of over-represented transcription factor-binding sites (TFBS) based on the genes affected by A20 overexpression. Significantly enriched TFBS were determined by promoter analysis of genes that were affected by A20 within the following networks: cell cycle, energy production, inflammation and immune system related, and lipid and carbohydrate metabolism. The number of genes impacted by a given transcription factor are reflected by the node size and statistical enrichment (P value), using a color scale (red for highest enrichment and blue for no enrichment). P values were derived from Fisher Exact test. The TFBS analysis was performed using ExPlain 3.0 (http://explain.biobase-international.com/) for detection of over-represented transcription factor binding sites. The analysis was performed on 2000 bp upstream to 100 bp downstream of the transcription start site of each gene.

We analyzed the promoter regions of genes within each of the highly enriched networks for the presence of over-represented transcription factor binding sites. Putative transcription factor binding sites were identified in each network based on a >2 fold enrichment (yes set versus no set) and on a P-value from a one-tailed Fisher's exact test >0.05 (Fig 5). We estimated the importance of each transcription factor by calculating the number of differentially regulated genes within each network that contained a putative binding site for this transcription factor. Based on this analysis, we identified a total of 21 putative transcription factors, with 6 of them found in more than one network (NF-κB, HSF2, CIZ, HNF1, IRF2, and OCT4). NF-κB, the central target of A20 [28], [29], was the only transcription factor common to all 4 networks analyzed including cell cycle, energy production, inflammation, and metabolic processes (Fig. 4). However, other than in the cell cycle network, where it had the greatest fold enrichment, NF-κB did not rank first in any of our significance metrics in the other networks, indicating a relatively small (even if broad and key) number of genes affected by NF-κB. The lipid metabolism and inflammation networks shared 2 transcription factors (IRF2 and OCT4), whereas the cell cycle and inflammation networks shared one transcription factor (HSF2), as did cell cycle and lipid metabolism (CIZ), and energy and lipid metabolism (HNF1). In the inflammation network 3 separate serum response factor (SRF) binding matrices met our selection criteria, highlighting the likely involvement of SRF in mediating A20's effects on liver regeneration.

### A20 modulates the expression of genes that are key to the liver's regenerative capacity

In order to determine how A20 affects an externally validated set of key genes involved in liver regeneration, we interrogated a previously published signature set of genes detailing the liver transcriptome response 24 hrs following liver resection [30]. CEL files were obtained from the Gene Expression Omnibus at the National Institutes of Health (http://www.ncbi.nlm.nih.gov/geo/) and processed with dChip, as outlined in the methods section. We identified a seed set of 157 genes that peaked or showed a nadir in their expression at 24 hrs and had an absolute LCB ≥ 2 from 0 to 24 hrs. When multiple probe sets existed for the same gene, the probe set with the larger LCB was used. By network analysis and after merging overlapping networks, we identified Key nodes by their edge density. We included all constituent sub-units and transcript variants, which helped us picking a set of 99 transcripts (93 of which mapped back to the microarray) that represented the 24 hrs liver regeneration signature.

We did hierarchal clustering using a correlation-based metric Pearson test, and rendered heat maps using standardized LCB values for the regeneration signature, the rAd.βgal and the rAd.A20 treated livers. The gene transcript profile was most similar between rAd.A20 treated livers and the regeneration signature, indicating that A20 overexpression compensated for any detrimental effects of viral transduction. These results highlight the fact that recombinant adenovirus transduction differentially impacts liver regeneration, and stresses the importance of using rAd.βgal transduced, rather than non-transduced livers, as a control group in our study.

Clustering based on the magnitude of the response of specific transcripts to resection (LCB) showed distinct sets of genes. Clusters I-b and III-a related to genes whose behavior was altered as a result of adenoviral transduction in control rAd.βgal transduced livers but not rAd.A20 overexpressing livers, indicating the ability of A20 to protect from adenoviral toxicity. Clusters I-a and III-b, represented genes that distinguished both of our sets of animals from the seed set; these likely represent differences relating to mouse strain (Balb/c versus C57BL/6), adenoviral effects that could not be compensated for by A20, and/or the extent of liver resection (78% versus 66%).

We identified two key gene expression clusters that were modified by A20 (Fig. S5). A20 upregulated the expression of a subset of signature liver regeneration genes, even prior to resection (Fig. S5 B), including many of the RNA polymerase subcomponents, indicating that the cells are ready to engage in cell replication. These genes were not subsequently upregulated following resection, as they had already fulfilled their regenerative need. The mirror image was also true (Fig. S5 C); A20 down-regulated the basal expression of a subset of signature genes that are usually only down-regulated upon liver resection, such as genes involved in MAP-kinase signaling and cytokine and growth factor responses, Many of these genes returned to their basal levels within 24 hours of liver resection, indicating that their rebound was accelerated in A20 overexpressing livers.

### In vivo validation of target genes and functional enrichment

**Figure 6:**
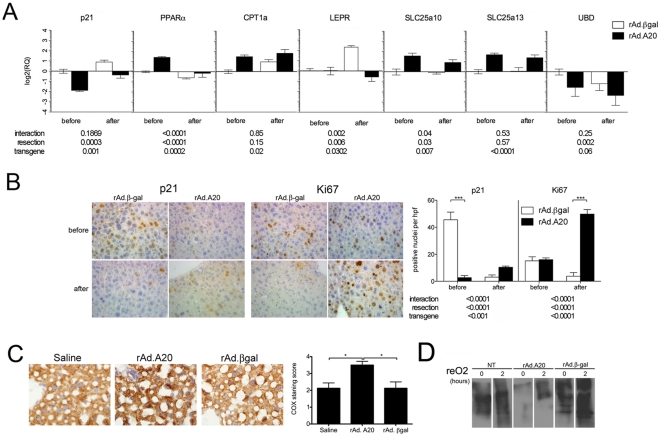
validation of A20 target genes in hepatocytes. **A)** qRT-PCR was performed on mouse liver samples harvested before and 24 h after extended LR. Graph represents the statistical analyzes of relative mRNA levels after normalization by βactin. Results are expressed as mean ± SEM of 4–6 animals. P values were calculated by using 2-way ANOVA for resection, transgene, and the interaction. P values were listed under the graph. Differences were considered significant when P<0.05. **B)** Representative photomicrographs showing decreased p21^waf1^ positive cells in liver samples transduced with recombinant adenovirus (rAd.)A20, as compared to control rAd. βgalactosidase (βgal) before resection and increased Ki67 staining 48 hrs after LR. Graph shows quantification of positive nuclei per high power field. Each bar represents the mean±SE from 4–6 different animals. Original magnification, X400. ***p<0.001. **C)** Representative photomicographs showing increased COX activity in saline, rAd.A20 and rAd. βgal transduced liver tissues 24 hrs following 78% LR. Graph shows scores of COX activity staining per high power field. Each bar represents the mean±SE from 4–5 different animals. *p<0.05. **D)** Representative Oxyblot of rat primary hepatocytes transduced with rAd.A20 or rAd. βgal control adenovirus for 48 h and exposed to 60 minutes hypoxia (1% O2) followed by 120 minutes reoxygenation. Cell lysates were immunoblotted for the detection of carbonyl groups (n = 3).

Key genes forming focus hubs within significantly modulated interaction networks from, GO groups and canonical pathways were selected for *in vivo* validation. Using qPCR, the expression of CDKN1A (p21), PPARα and its target gene the mitochondrial enzyme Carnitine Palmytyol Transferase 1 (CPT1a) [31], the mitochondrial solute carrier genes SLC25A10 and SLC25A13, as well as the IFNγ target gene Ubiquitin D (UBD), were measured in all samples (Fig. 6A). CDKN1A/p21 levels demonstrated significant changes in expression before and 24 hrs after resection, as compared to rAd.βgal controls: while both rAd.A20 and rAd.βgal treated livers underwent significant up-regulation of p21 levels in response to resection, rAd.A20 livers had significantly less p21 expression to start with, and as a result continued to have significantly lower levels following resection, as demonstrated by qPCR (Fig. 6A). These results suggest that A20 overexpressing hepatocytes were pre-primed to proliferate. At the protein level by immunohistochemisty, we showed significantly less nuclear p21 immunostaining at baseline in rAd.A20 treated livers, as compared to rAd.βgal treated controls, which conversely translated in significant increase of proliferating hepatocytes, as indicated by the number of Ki67 positive cells in rAd.A20, as compared to rAd.βgal treated livers, 48 hrs after resection (Fig. 6B). At that time, i.e. 48 hrs following resection, p21 protein levels had significantly decreased in rAd.βgal expressing livers of the animals that had survived the surgery, indicating delayed regeneration was about to proceed. Hepatocyte proliferation started by day 4 to 7 after 78% LR in rAd.βgal expressing livers, as indicated by Ki67 staining (data not shown), and as reported in other models of delayed liver regeneration [32]. Since we have not measured p21 mRNA at 48 hrs after LR, we do not know if this is related to decreased p21 transcription at this time point or to post-transcriptional or post-translational block in p21 expression. In contrast, p21 protein levels were higher in rAd.A20 expressing livers 48 hrs following LR as expected in livers that had undergone sufficient regeneration and needed to stop the proliferative process [33] (Fig. 6A).

A20 overexpression also resulted in significant up-regulation of PPARα mRNA levels in mouse livers at baseline, and prevented their down-regulation following extended LR, as demonstrated by qPCR (Fig. 6A). We checked that PPARα was functional in the system by showing that its upregulation translated into a significant increase in carnitine palmytoyl transferase 1a (CPT1a) mRNA (qPCR), a transcriptional target of PPARα and critical metabolic regulator of fatty acid (FA) metabolism, both before and after 78% LR (Fig. 6A).

PPARα is known to promote a net bioenergetics gain in the cells through promoting mitochondrial ATP production. Accordingly, we checked for cytochrome c oxidase (COX), i.e. mitochondrial oxidation phosphorylation (OXPHOS) complex IV, activity in saline, rAd.A20 and rAd.βgal treated livers 24 hours following LR, by means of an enzymatic histology method, as described in the methods section. Our data indicate that A20 overexpressing livers show a significant increase in mitochondrial cytochrome c oxidase activity in the liver post LR, a direct indication of enhanced energy production in these organs (Fig. 6C, n = 4–5 animals per group; p<0.05).

PPARα being also an important modulator of the response to oxidative stress and lipid peroxidation, oxidoreductase activity and oxidation/reduction processes were both significantly enriched in our gene ontology (GO) analysis. In order to validate this finding and determine the net directionality of A20's effect, we performed OxyBlots on whole cell lystate from primary derived rat hepatocytes that were subjected to 60 min. of hypoxia, followed by 120 min. of reoxygenation, as a way to *in vitro* model the oxidative stress imposed on hepatocytes, such as in the setting of LR or ischemia reperfusion injury. Equal amounts of proteins were carefully loaded in the gels, then analyzed for protein oxidation. Our results showed decreased protein oxidation in rAd.A20-transduced hepatocytes relative to either non-transduced or rAd.βgal transduced hepatocytes, both immediately following hypoxia and 2 hours after reoxygenation (Fig. 6D). Combined with the GO process and function enrichment data, such *in vitro* validation argues for a novel anti-oxidant affect of A20, that we had demonstrated to be, least in part, PPARα-dependent [23].

Additionally, a number of mitochondrial carrier genes that shuttle a variety of metabolites across the inner mitochondrial membranes were also up-regulated in A20 overexpressing livers, as determined by qPCR of mouse liver RNA before and after 78% LR (Fig. 6A). These include mitochondrial solute carrier family 25 members 10 (Slc25a10), referred to as dicarboxylase carrier (DIC), and SLC25 member 13, referred to as Aspartate Glutamate Carrier (AGC2) or Citrin [34]. Both these proteins are involved in lipid and glucose metabolism, contributing to increasing the energy supply required for cellular functions.

By qPCR we also demonstrated that overexpression of A20 in the liver significantly abrogated the upregulation of the leptin receptor following 78% LR (Fig. 6A). The significance of this finding is being explored.

We also determined that Interferon signaling was significantly affected by A20 overexpression in the liver, with type II Interferon (IFNγ) being a significant node. Although IFNγ levels *per se* were not affected, IFNγ target genes were significantly down-regulated in rAd.A20, as opposed to rAd.βgal treated livers, indicating a disruption in IFNγ signaling. Accordingly, overexpression of A20 reduced the mRNA levels of the IFNγ-target gene, UbiquitinD (UBD)/FAT10 in rAd.A20, as opposed to rAd.βgal treated livers, as demonstrated by qPCR (Fig. 6A). UBD/FAT10 is a ubiquitin-like molecule that could be involved in post-translational modifications of proteins, and hence may further add to the ubiquitin-editing function of A20 [35], [36].

## Discussion

Liver regeneration is a physiologic repair mechanism that occurs following liver damage as a result of toxic, viral, metabolic, or ischemic insults, or after liver resection for tumor or living donation. When liver regeneration is impaired, liver failure ensues leading to significant morbidity or even death, unless the patients undergo liver transplantation. Accordingly, defining the molecular basis of liver regeneration could be instrumental in unveiling novel therapeutic tools to optimize this process in the clinical setting.

We have identified A20 as a potent hepatoprotective protein that is part of the physiologic anti-apoptotic, anti-inflammatory and regenerative response of the liver [11], [21], [23], [37]. Overexpression of A20 in the liver afforded a significant survival and functional advantage in mouse models of extreme liver injury and resection. In patients undergoing living donor liver transplantation (LDLT), expression of A20 also significantly increased 1 hour after reperfusion, as determined by gene microarray analysis, validating the fact that A20 is part of the regenerative response in humans [20]. Based on the above, we engaged in this study to clarify the effect of A20 overexpression on the transcriptome in an extended LR model. Extended LR is plagued with increased lethality as a result of “small for size” remnant liver mass, and hence best reproduces massive liver resections in patients with large tumors, or small for size liver grafts, as sometimes seen following LDLT [38].

Even if does not establish a dose-effect relationship between A20 and the different target genes identified this microarray-based analysis of the transcriptome in livers before and 24 hours after 78% LR, provided insight into the global transcriptional network that was directly or indirectly affected by overexpression of A20. Indeed, we demonstrated that A20 targeted multiple pathways that were, as expected, central to cell cycle and immune and inflammatory responses of the liver, but also unraveled novel pathways impacted by A20 such as oxidoreductase balance, and biosynthetic and metabolic liver processes. Systems Biology based analysis of the data allowed for the first time to depict a global picture of the regenerative process and its interactive pathways, and to highlight the central nodes affected by A20 overexpression, when compared to liver regeneration signature, as recently determined in Dr. Karp's study in mice[30]. Interestingly, our data analysis also demonstrated that the effect of A20 preceded the resection, further highlighting the impact of A20 on the liver transcriptome, independently from the changes in gene profile that are triggered by LR.

This approach stressed the effect of A20 overexpression in hepatocytes upon optimizing and enhancing lipid metabolism, in a way that is likely to increase energy production, hence providing extra fuel to support the regenerative response. This outcome is ideal, as it relieves the need to shunt energy away from other cellular activities, namely the synthetic function of hepatocytes, and hence avoids the usual impairment of the liver metabolic functions when the organ is engaged in extensive proliferation. Remarkably, most of the effects of A20 on these pathways were present at baseline, and maintained following LR, indicating that A20 overexpressing livers were prepared to face a greater challenge than their control counterparts that require some time before mounting a regulatory response to resection.

By pathways, overexpression of A20 down-regulated overall inflammation and inflammatory signaling in the liver, as expected for a NF-κB-inhibitory protein [19], [29], [39].

The other most notable pathway that was obviated by our analysis relates to the significant impact that A20 had on cell cycle in hepatocytes. A thorough mining of the data demonstrated that overexpression of A20 led to a significant down-regulation of the universal inhibitor of Cyclin/CDK activity, CDKN1A/p21, before and after 78% LR. CDKN1A plays an important role in regulating hepatocyte cell cycle progression and exit during morphogenesis, differentiation, and repair [40], [41], [42]. The precise regulation of p21 expression is mandatory for synchronization of the first wave of hepatocyte proliferation following resection [43]. This has been clearly demonstrated in p21 knockout mice that show accelerated hepatocyte proliferation following LR [44]. Therefore, reduced levels of p21 in A20 over-expressing livers positions hepatocytes in a “pre-primed” state, engaging them in an accelerated response to the proliferative stimuli triggered by LR.

Lipid metabolism was another pathway that was significantly impacted by A20. It is well established that mobilization of peripheral fat, hepatic FA uptake, and rapid accumulation of intracellular triglycerides in hepatocytes are important for optimal liver regeneration [45], [46]. Cytokine signals after liver injury or LR promote the release of FA into the circulation and their uptake by hepatocytes [47]. As a result, lipid droplets form in hepatocytes during the early stages of liver regeneration, providing energy to fuel the proliferative response. Even though this transient steatosis is part of the physiologic proliferative response, when excessive or uncontrolled, it can impair liver regeneration [48], [49]. Several signals and molecules contribute to lipid accumulation during the regenerative process including Leptin signaling [50], hepatic Glucocorticoid-Receptor signaling, and Caveolin-1[51], [52]. Our results show no evidence for A20 affecting the expression of Caveolin-1 or the Glucocorticoid-receptor complex, but obviates that overexpression of A20 significantly inhibits the up-regulation of the Leptin-receptor following 78% LR (31-339, Suppl. Table 2). This points to an intriguing regulation of this system by A20 and supports the hypothesis of Newberry *et a*l and Leclercq *et a*l, that Leptin signaling *per se* is not directly involved in liver regeneration, but rather required to promote the inflammatory and priming response, following PH [50], [53]. Accordingly, when the priming response and the energy stores are already amplified by A20, we surmise that upregulation of the Leptin receptor is no longer required.

Besides priming, allocation of energy is the second crucial step for the hepatocyte before entering cell cycle. Proliferating hepatocytes require triglycerides as an energy source [54], [55]. Generation of ATP form triglycerides relies on mitochondrial β-oxidation as well as microsomal omega-oxidation of FA. One of the main regulators of this process is PPARα, a central node affected by A20 in our system. PPARα, a member of the nuclear receptor superfamily, is a key player in the liver's response to metabolic dysregulation, inflammation, injury and resection [56]. Accordingly, PPARα knockout mice have impaired liver regeneration[14]. Our data demonstrates a significant increase in PPARα and of its downstream target CPT1a expression before LR in livers of A20 treated mice, indicating that A20 overexpressing livers are pre-set to ramp-up their energy supply following LR, as to meet increased demand of proliferating hepatocytes. This advantage remained following 78% LR, as A20 expressing livers showed less decrease in PPARα and CPT1a levels than βgal-treated livers. We present strong evidence confirming this hypothesis by demonstrating increased mitochondrial COX activity in A20 expressing livers as opposed to controls, 24 hrs following 78% LR.

PPARα also enhances the degradation of lipid-derived inflammatory mediators by promoting β-oxidation i.e. “fat burning”, hence shunts these mediators away from the lipid peroxidation (LPO) pathway, decreasing reactive oxygen species (ROS) burden [47], [56]. Decreased ROS in turn limits inflammation [57], [58], and protects hepatocytes from apoptosis [59], [60], which adds to the anti-inflammatory and anti-apoptotic function of A20 in hepatocytes.

In addition, overexpression of A20 in the livers increased the expression of the mitochondrial solute carriers DIC/SLC25a10 and Citrin/SLC25a13, which has the potential to further optimize energy supply, and improve hepatocyte survival in A20-overexpressing livers [61], [62].

Our data also demonstrated a profound decrease in IFNγsignaling in A20 overexpressing livers. Whereas, most of the other pathways that we evaluated were based on their implication in the response to the procedure (i.e. surgical resection), the IFNγ pathway came up in our analysis probably because of the adenoviral gene therapy vector we used [63]. Reduced IFNγ signaling in A20 overexpressing livers reflects the ability of A20 to dampen the anti-viral immune response, which would be in keeping with previous data demonstrating that A20 decreases anti-viral responses [64].

Remarkably, the number of transcription factors upstream of genes modified by A20 highlighted the impact of A20 on affecting all NF-κB dependent genes, but also unraveled a number of other transcription factors that were not so far identified as targets of A20. These included E2F, CEBPA, EGR1, OCT4 and HNF1 that were all over-represented in the promoter of genes impacted by A20. Future work is aimed at determining whether A20 affects directly or indirectly the activity of these transcription factors.

In summary, this Systems Biology-based analysis depicting the impact of overexpression of A20 on liver function following extended LR stresses the critical role of A20 in promoting liver regeneration and function by optimizing energy production and usage through improved lipid metabolism and down-regulation of inflammation. In turn, this spares the need for energy consumption involved in immune activation, therefore channeling the supplies towards regenerative and metabolic functions of the liver. Ongoing loss of function experiments using A20 +/− mice totally support this statement (Studer *et al*, manuscript in preparation).

Our results are particularly important in light of the recent clinical validation of A20/tnfaip3 but also p21, SOCS3, and PPARα, as part of the integral regenerative response of the liver in the setting of LDLT [20], which supports our pursuit of A20-based therapies to improve the outcomes of LDLT, marginal liver grafts, and after extensive resections for tumor.

## Materials and Methods

### Ethics statement

The Institutional Animal Care and Use Committee of the Beth Israel Deaconess Medical Center (BIDMC) approved all experimental projects. All animal use is in compliance with all current US government regulations concerning the care and use of laboratory animals. There are no veterinary concerns related to the use of mice as performed in this paper. Supervision of animal care was conducted by staff members of a fully Association for Assessment and Accreditation of Laboratory Animal Care (AAALAC) accredited facility headed by Dr. Garibaldi. All personnel handling the animals followed a specialized training prior to starting. The BIDMC has been certified for Animal Welfare Assurance. The number is A3153-01, expiring on 2/28/2014. The approved protocol number is #064-2008.

### Recombinant adenoviruses

Recombinant adenoviruses (rAd.) encoding full length human A20 or the control β-galacatosidase (βgal) were generated in our laboratory, as previously described [65]. A20 or β-galactosidase (βgal) gene expression in the mouse liver was achieved by penile vein injection of 1x10^9^ pfu of rAd. in 100 µL of normal saline, which results in optimal transgene expression 5 days after injection in 30% to 40% of hepatocytes [11].

### Surgical model of liver resection

Extended (78%) LR, consisting of resection of the lateral, medial, left, and right lobes, was performed 5 days following rAd. administration in 8-week old BALB/c mice weighing 25 to 30 grams (Taconic, Germantown, NY), as described [11]. We took extreme care to avoid blood loss and maintain euvolemia and normal body temperature [11]. RNA was extracted from the resected portion of the liver (before samples) and from the remnant liver 24 hours after resection (after samples). Animals received care according to the criteria outlined in the *Guide for the Care and Use of Laboratory Animals*. All procedures were approved by the Institutional Animal Care and Use Committee.

### Data analysis

For transcriptional profiling, the mouse genome 430 2.0 Affymetrix GeneChip, containing more than 45,000 transcripts, was used. RNA from three animals was pooled per microarray and 2 microarrays per group were analyzed. Microarray analysis was conducted by the Genomics Center at the Beth Israel Deaconess Medical Center according to previously described protocols for total RNA extraction and purification, complementary DNA (cDNA) synthesis, *in vitro* transcription for production of biotin-labeled cRNA, hybridization of cRNA with mouse genome 430 2.0 Affymetrix gene chips, and scanning of image output files [66]. The raw gene expression data, in MIAME compliant format was submitted to GEO database at NCBI for public access (GSE24203). The quality of scanned arrays images were determined on the basis of background values, percent present calls, scaling factors, and 3′–5′ ratio of βactin and GAPDH using the simpleaffy package for R [30].

Scanned array images were analyzed by dChip. The raw probe level data was normalized using smoothing-spline invariant set method and the signal value for each transcript was summarized using the PM-only based signal modeling algorithm in which the signal value corresponds to the absolute level of expression of a transcript [25]. Normalized and modeled signal values for each transcript were used for all further high-level bioinformatics analysis. During the calculation of model based expression signal values, array and probe outliers were interrogated and image spikes were treated as signal outliers. The outlier detection was carried out using dChip outlier detection algorithm. A chip was considered to be an outlier if the probe, single or array outlier percentage exceeded a default threshold of 5%. To compare probe expression values, 90% lower confidence bound (LCB) of the fold change (FC) was calculated using dChip.

### Informative genes

A set of informative genes was initially created from the union of probes with an absolute expression ≥ 40 and LCB ≥1.5 for each of the following sample comparisons: rAd. βgal after LR (post-βgal) vs. rAd. βgal before LR (pre-βgal); rAd.A20 after LR (post-A20) vs. rAd.A20 before LR (pre-A20), rAd.A20 before LR (pre-A20) vs. rAd. βgal before LR (pre-βgal), or rAd.A20 after LR (post-A20) vs. rAd.βgal after LR (post-βgal). The before and after comparisons identify genes differentially affected by A20, while the A20 and βgal comparisons identify genes differentially affected by extended LR. To focus on the effect of A20, subsequent analysis, was limited to genes that met the following criteria: after LCB ≥ 1.5 or before LCB ≥ 1.5 or (A20 LCB ≥ 1.5 and βgal LCB no ≥ 1.5). In order to quantify the effect of A20 on gene expression, we calculated an interacting score using Equation 1:

Y_i_  =  (rAd.A20_pre,i_ − rAd. βgal_ pre, i_) + (rAd.A20_ post, i_ − rAd. βgal _post, i_). EQ 1

Where Yi is interaction score for each transcript generated using the log2 transformed expression of each transcript in before (pre,i) and after (post,i) resection conditions.

This equation captures the effects of A20 on baseline gene expression as well as on the genes differentially affected by liver resection; i.e. genes with the absolute highest scores affected the most by A20 overexpression. We reduced the list of differentially expressed probes to unique genes by selecting for the probe set with the largest absolute interacting score among all the probe sets mapping to the same Entrez ID using the BioConductor package Genefilter [52], [54], [67].

### Self Organizing Maps (SOM)

In order to identify gene expression profiles that are functionally related to the biological state of interest, we performed SOM clustering on transcript expression values using Pearson correlation coefficient based distance metrics and a target of 100 groups. SOM allow the grouping of gene expression patterns into an imposed structure in which adjacent clusters are related, thereby identifying sets of genes that follow certain expression patterns across different conditions [68].

### Enrichment, pathway, and network analysis

In order to identify over-represented gene ontology categories from the informative set of genes, we used the Cytoscape plugin BiNGO [12], [26], [27]. BiNGO maps the functions impacted by a given gene set on the GO hierarchy and outputs this mapping as a Cytoscape graph that assists in understanding biological theme. The mouse genome was used as the background set of genes and P-value correction was performed by the method of Benjamani and Hochberg [69]. Additionally for analyzing enrichment in SOM clusters, we used the Database for Annotation, Visualization and Integrated Discovery (DAVID) [6], [55].

We analyzed interactive networks and pathways using the commercial system biology oriented package Ingenuity Pathways Analysis (IPA 4.0) (http://www.ingenuity.com/). The knowledge base of this software consists of ontology and network models derived by systematically exploring the peer reviewed scientific literature. It calculates the P value for each network, and pathway according to the fit of user's data to IPA database. It displays the results as score (-log *P value*) indicating the likelihood of a gene to be found in a network or pathways by random chance. For example, a network achieving a score of 2 has at least 99% confidence of not being generated by chance alone.

### Promoter analysis

We performed promoter analysis using the online tool ExPlain 3.0 (http://explain.biobase-international.com/) for detection of over-represented transcription factor binding sites. ExPlain uses MatchTM, a weight matrix-based tool for searching putative transcription factor binding sites [42], [49]. For the analysis we selected regions between 2000 bp upstream to 100 bp downstream of the transcription start site of each gene (Yes set) that were conserved between human, mouse, and rat. The enrichment was calculated against a random set of promoters obtained from mouse housekeeping genes (No set). We used the entire vertebrate non-redundant set of transcription factors matrix from transfac database in scanning for potential binding sites [70]. In addition to promoter analysis, we used the exPlain package to identify the key “upstream” signaling molecules that account for the observed gene expression profile.

### Quantitative Real Time PCR

cDNA was prepared using the iScript cDNA Synthesis Kit (BioRad USA, Hercules, California). Quantitative PCR reactions were prepared in duplicate using iTaq Fast SYBR Green Supermix With ROX (BioRad) and gene specific primers (Table S1). qPCR was performed on a 7500 Fast Real-Time PCR System (Applied Biosystems, Foster City, California). β-actin was used as an internal housekeeping reference. Gene expression was quantified using the relative quantification method of Livak [71]. We analyzed qPCR results by both one- and two-way ANOVA (Prism 5.0, GraphPad Software, La Jolla, California) for matched pairs, when possible.

### In vitro hypoxia/reoxygenation experiments

Rat primary hepatocytes were purchased from Cambrex (Lonza, Walkersville, Maryland) and cultured at 37°C in 5% CO_2_ according to the supplier specifications. Cells were transduced with rAd.A20 or the control rAd. βgal at a multiplicity of infection of 100, which achieves transgene expression in >95% of cells at 48 hrs with minimal toxicity. At 48 hrs after transduction cells were subjected to 60 min of hypoxia at 1% O_2_, followed by incubation under normoxic conditions for 120 min. Protein lystates were then extracted and immunodetection of carbonyl groups was performed by OxyBlot (Millipore, Billerica, Massachusetts).

### Enzymatic histology method for detection of Cytochrome c Oxidase (COX, mitochondrial complex IV) activity in liver tissues

COX couples the transfer of electrons from reduced cytochrome c to oxygen, and is a good biomarker for the bioenergetics function of the mitochondria. In brief, snap frozen tissue sections from saline, rAd.A20 and rAd. βgal transduced livers are treated with an incubation mediun (5 mg of the electron donor 3–3′ diaminobenzidine tetrahydrochloride (DAB), 10 mg of cytochrome c and 20 µg of catalase in 10 ml of phosphate buffer, pH 7.4) for 15 min. in a humidity chamber at 37°C. This was followed by rinsing, counterstaining with hematoxylin, ethanol dehydration and Xylene treatment. In this context DAB produces an insoluble brown product that reflects the function of complex IV. This method has shown excellent mitochondrial specificity in several tissues including skeletal muscle and liver[72]. Intensity of the brown staining per section was scored by two investigators blinded to the study. Scores ranged from 1 to 4 from fainter to stronger. Significance between groups was analyzed by ANOVA.

### Immunohistochemistry

Tissue samples from rAd.A20 and rAd. βgal treated livers were recovered before and 48 hrs after extended LR, and fixed in 10% buffered formalin. Five µm sections were immunostained with Ki67 (Dako, Glostrup, Denmark) and anti-p21^waf1^ (BD Biosciences, San Jose, CA) antibodies. Ki67 and p21 positive cells were counted from 3 high power fields (hpf) per mouse liver by two investigators blinded to the study.

## Supporting Information

Figure S1 **Expression of the transgenes in rAd.tranduced livers and time table protocol. A)** Expression of human A20 and nuclear E. Coli β-galactosidase in all mouse livers used for microarray analysis was verified by qPCR, and by Xgal staining for the βgalactosidase transgene in mouse livers 5 days following rAd. iv injections. **B)** Protocol of transgene delivery prior to liver resection in mice.
(TIF)

Figure S2 **Principal component analysis of transcriptional data.** Open and filled blue and red circles represent control (rAd. βgal) and rAd.A20 arrays respectively. The first principal component with highest variation (47%) is shown on the X-axis and separates the arrays on the basis of resection status (before and after resection). The second component with median variance (26%) is displayed on the Y-axis and separates the arrays on the basis of the treatment (rAd. βgal vs. rAd.A20 arrays). An inset with overall correlation of the arrays is also shown as a dendrogram. The clustering depicts two major clusters on the basis of resection status.
(TIF)

Figure S3 **Hierarchical map of significantly enriched Gene Ontology (GO) categories including biological processes, molecular functions and cellular components affected by A20 overexpression. (A)** GO categories that are significantly affected are shown by pseudocolor shading within the hierarchical tree of GO classification. Each node corresponds to one GO category. Each Branch starts with a general category and divides stepwise into more specific categories. Highlighted regions within the hierarchical tree represent significantly affected groups of GO Categories, namely [I] cell cycle, [II] regulatory processes, [III] immune responses, (IV) molecular functions related to oxidoreductase, electron carrier activity, and oxidation-reduction processes, V) cellular components (details not shown), and VI) metabolic processes involved in amino acid, fatty acid, steroid, lipid, and cholesterol biosynthesis. The number of genes in each node is reflected by the node size and statistical enrichment (P value) using a pseudo color scale (Orange for highly enriched, yellow for significantly enriched and white for no enrichment). P values are derived from hypergeometric test adjusted using Benjamini-Hochberg. The GO categories graph was prepared using the Cytoscape plugin, BiNGO.
(TIF)

Figure S4 **Network of molecular interactions based on the set of 1,595 differentially expressed unique genes.** This network was derived by merging the top 15 networks that were the most significantly affected by A20 overexpression. Key nodes (shown in BOLD) are determined based on edge density, and include the following molecules: CDKN1A (p21), RB1, CDK2, CyclinA, PPARα, IFNγ, and AP1. The intensity of the node color indicates the degree of up-regulation (red), down-regulation (green) or no effect (white).
(PDF)

Figure S5 **Validation of the role of A20 in liver regeneration by comparing with external transcriptional data.** The behavior of 93 liver regeneration signature genes obtained from a previously published data set in mice were evaluated in rAd.A20 and rAd. βgal treated livers. The heat map depicts the LCB values of transcripts obtained by comparing before and after resection groups in the seed set (regeneration signature), the βgal and the A20 groups. The genes form four major clusters (I-IV) that consist of genes with different expression patterns. The columns represent the βgal, A20 and regeneration signature sets and the rows represent the 93 genes that are modified upon LR. Gene expression is shown with pseudocolor scale (−3 to 3) with red denoting high LCB (gene up regulation) and green denoting low LCB (gene down regulation). **B)** Subset of genes involved in regeneration that are upregulated by A20 even prior to resection. **C)** Subset of genes involved in regeneration that are down regulated by A20 even prior to resection.
(TIF)

Table S1 **List of primers used in real-time PCR.**
(PDF)

Table S2 **List of 1,595 unique genes, whose expression is uniquely affected by A20.** The genes were identified in any of following comparison: i) βgal after vs. before resection, ii) A20 after vs. before resection, iii) A20 before resection vs. βgal before resection, or iv) A20 after resection vs. βgal after resection. The gene list was divided in 15 sections on the basis of the Venn diagram shown in Fig. S2. The table depicts the average expression level of the gene in A20 before and after resection as well as βgal before and after resection groups.
(PDF)

Table S3 **Gene Ontology analysis of SOM patterns.** This analysis was performed using the Database for Annotation, Visualization and Integrated Discovery (DAVID ) v6.7 and gene ontology categories with multiple test (Holm–Bonferroni method) corrected P value <0.05 were considered significant.
(PDF)
