# Supplementary figures and images for: A20 Modulates Lipid Metabolism and Energy Production to Promote Liver Regeneration
Source: PLoS One. 2011 Mar 17;6(3):e17715. doi: 10.1371/journal.pone.0017715 (PMC3060102; doi:10.1371/journal.pone.0017715)

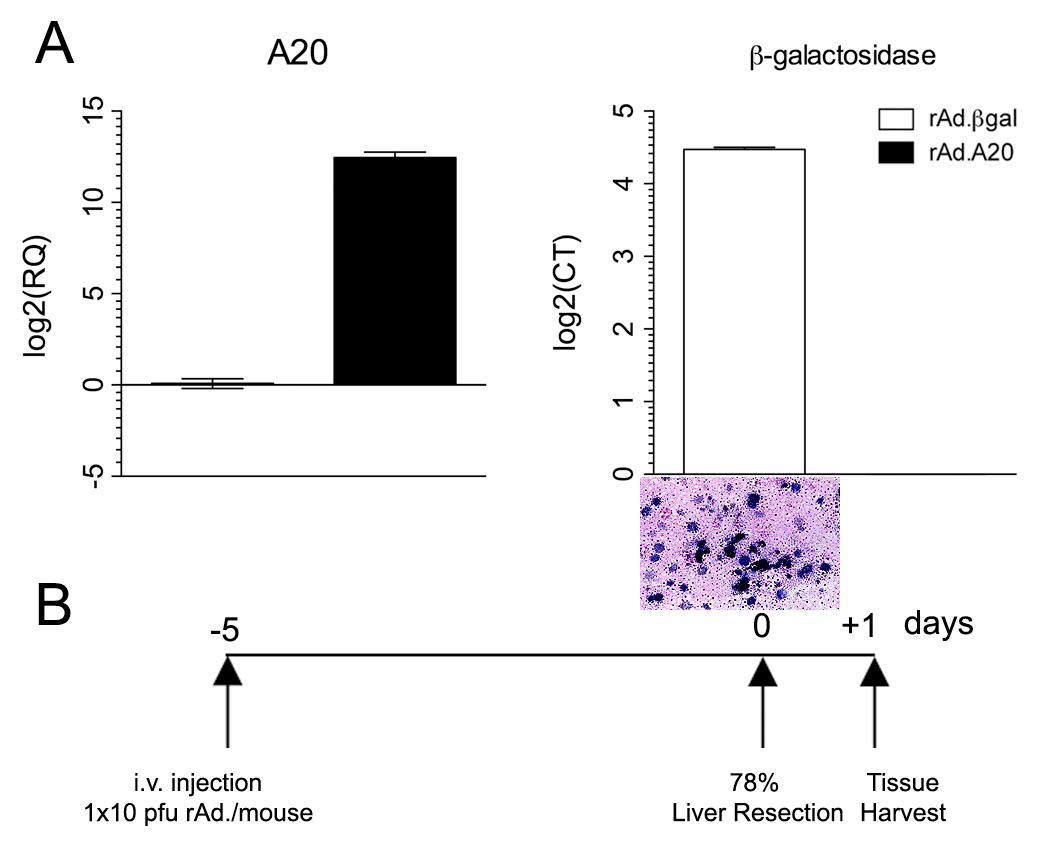

Supplement: Figure S1 — Expression of the transgenes in rAd.tranduced livers and time table protocol. A) Expression of human A20 and nuclear E. Coli β-galactosidase in all mouse livers used for microarray analysis was verified by qPCR, and by Xgal staining for the βgalactosidase transgene in mouse livers 5 days following rAd. iv injections. B) Protocol of transgene delivery prior to liver resection in mice. (TIF) [file pone.0017715.s001.tif]

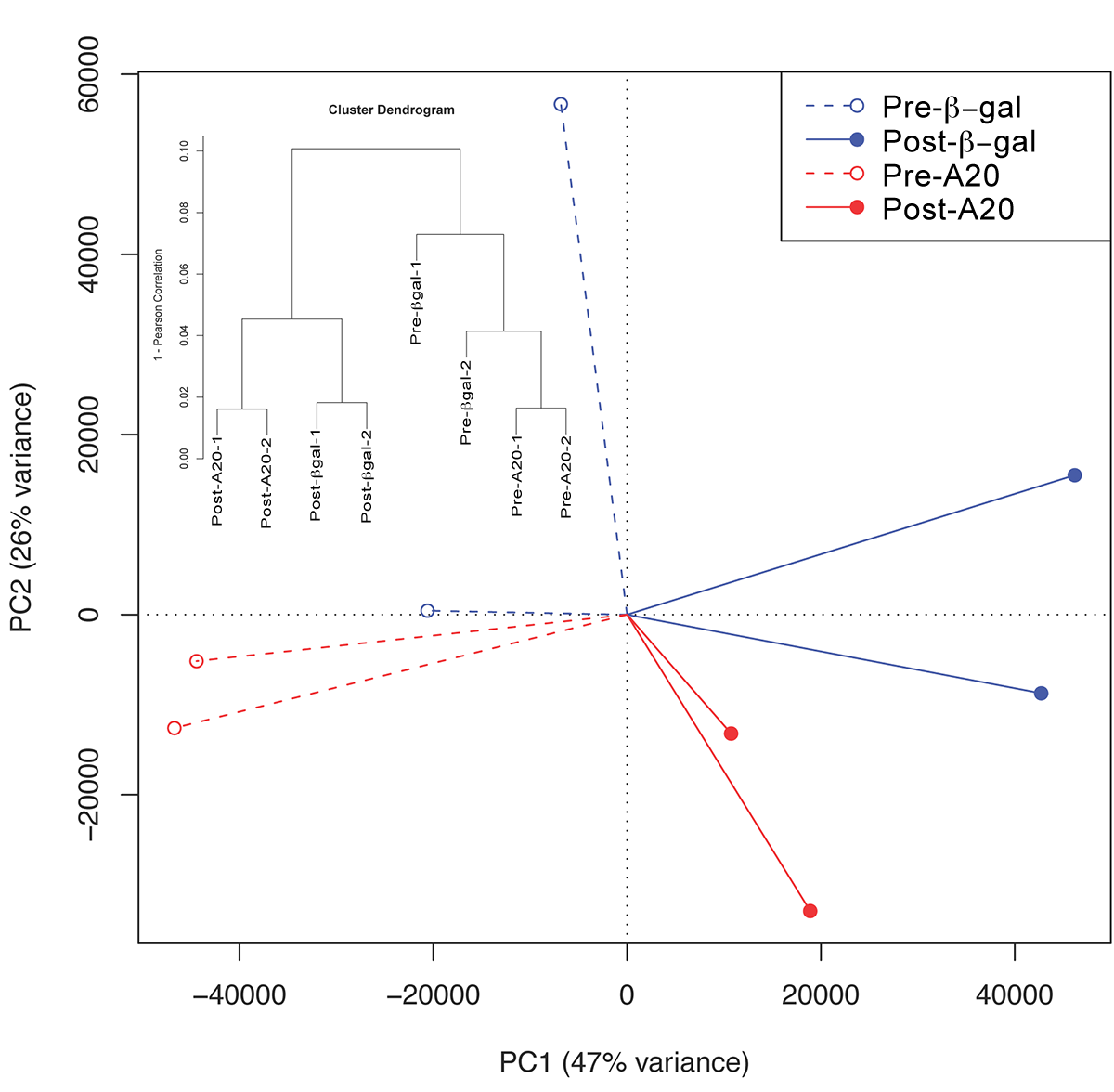

Supplement: Figure S2 — Principal component analysis of transcriptional data. Open and filled blue and red circles represent control (rAd. βgal) and rAd.A20 arrays respectively. The first principal component with highest variation (47%) is shown on the X-axis and separates the arrays on the basis of resection status (before and after resection). The second component with median variance (26%) is displayed on the Y-axis and separates the arrays on the basis of the treatment (rAd. βgal vs. rAd.A20 arrays). An inset with overall correlation of the arrays is also shown as a dendrogram. The clustering depicts two major clusters on the basis of resection status. (TIF) [file pone.0017715.s002.tif]

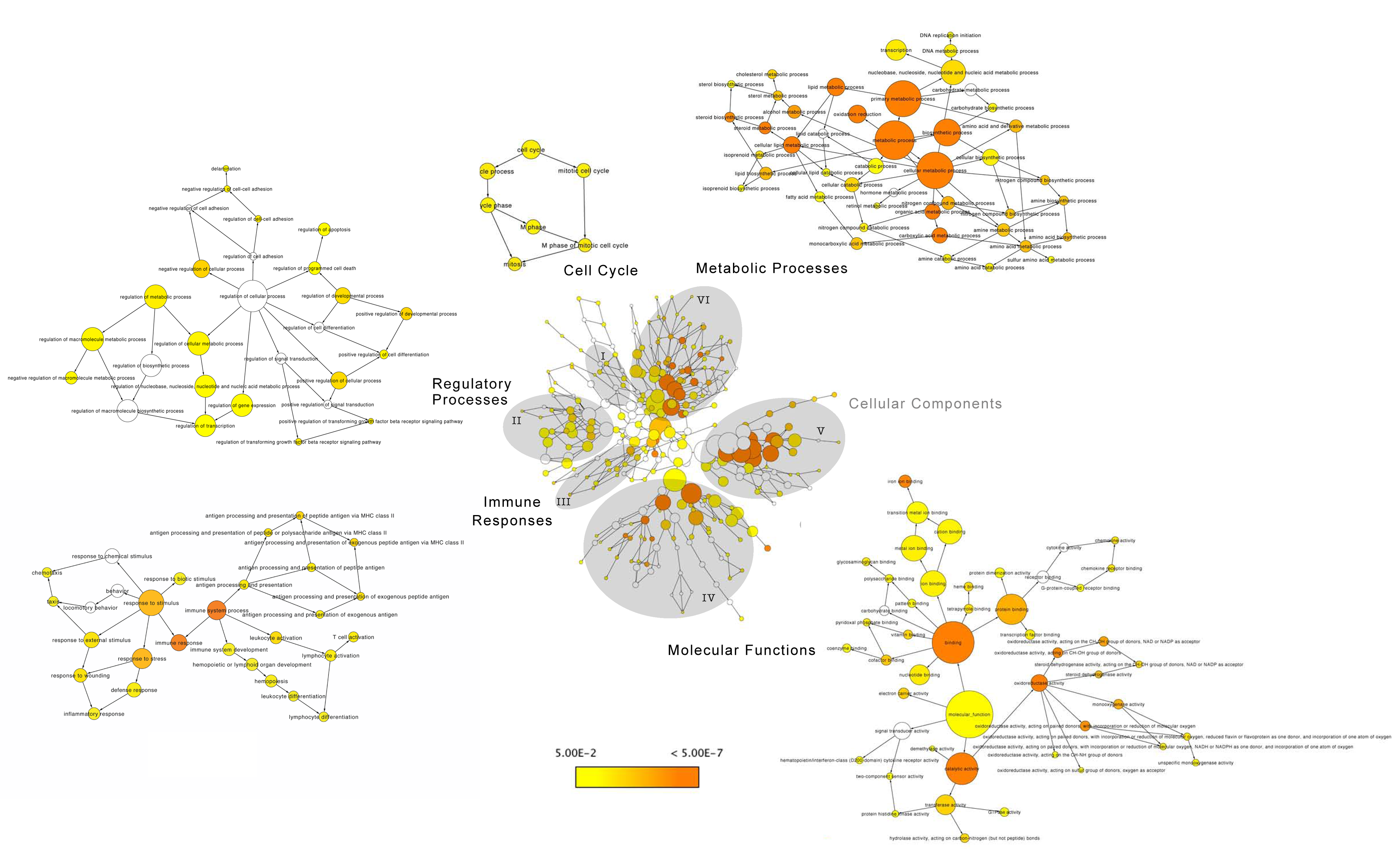

Supplement: Figure S3 — Hierarchical map of significantly enriched Gene Ontology (GO) categories including biological processes, molecular functions and cellular components affected by A20 overexpression. (A) GO categories that are significantly affected are shown by pseudocolor shading within the hierarchical tree of GO classification. Each node corresponds to one GO category. Each Branch starts with a general category and divides stepwise into more specific categories. Highlighted regions within the hierarchical tree represent significantly affected groups of GO Categories, namely [I] cell cycle, [II] regulatory processes, [III] immune responses, (IV) molecular functions related to oxidoreductase, electron carrier activity, and oxidation-reduction processes, V) cellular components (details not shown), and VI) metabolic processes involved in amino acid, fatty acid, steroid, lipid, and cholesterol biosynthesis. The number of genes in each node is reflected by the node size and statistical enrichment (P value) using a pseudo color scale (Orange for highly enriched, yellow for significantly enriched and white for no enrichment). P values are derived from hypergeometric test adjusted using Benjamini-Hochberg. The GO categories graph was prepared using the Cytoscape plugin, BiNGO. (TIF) [file pone.0017715.s003.tif]

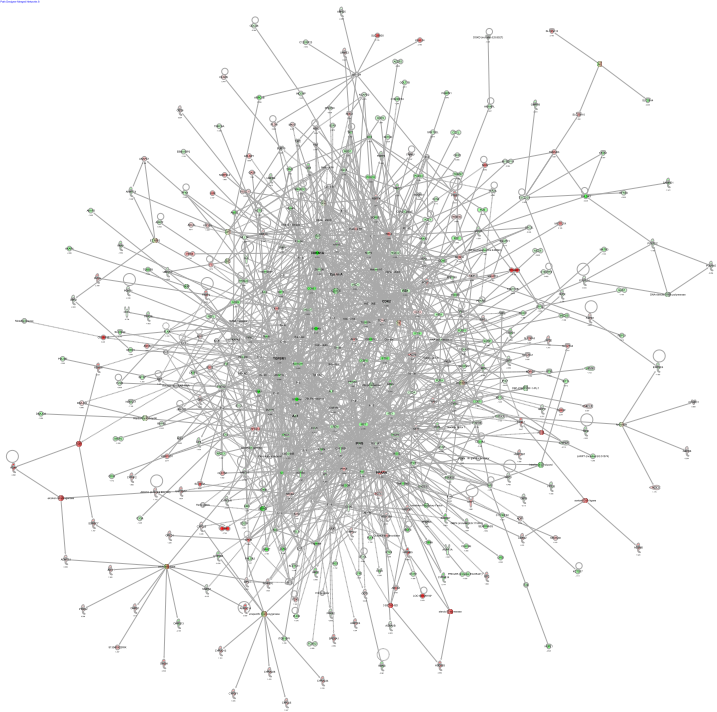

Supplement: Figure S4 — Network of molecular interactions based on the set of 1,595 differentially expressed unique genes. This network was derived by merging the top 15 networks that were the most significantly affected by A20 overexpression. Key nodes (shown in BOLD) are determined based on edge density, and include the following molecules: CDKN1A (p21), RB1, CDK2, CyclinA, PPARα, IFNγ, and AP1. The intensity of the node color indicates the degree of up-regulation (red), down-regulation (green) or no effect (white). (PDF) [file pone.0017715.s004.pdf]

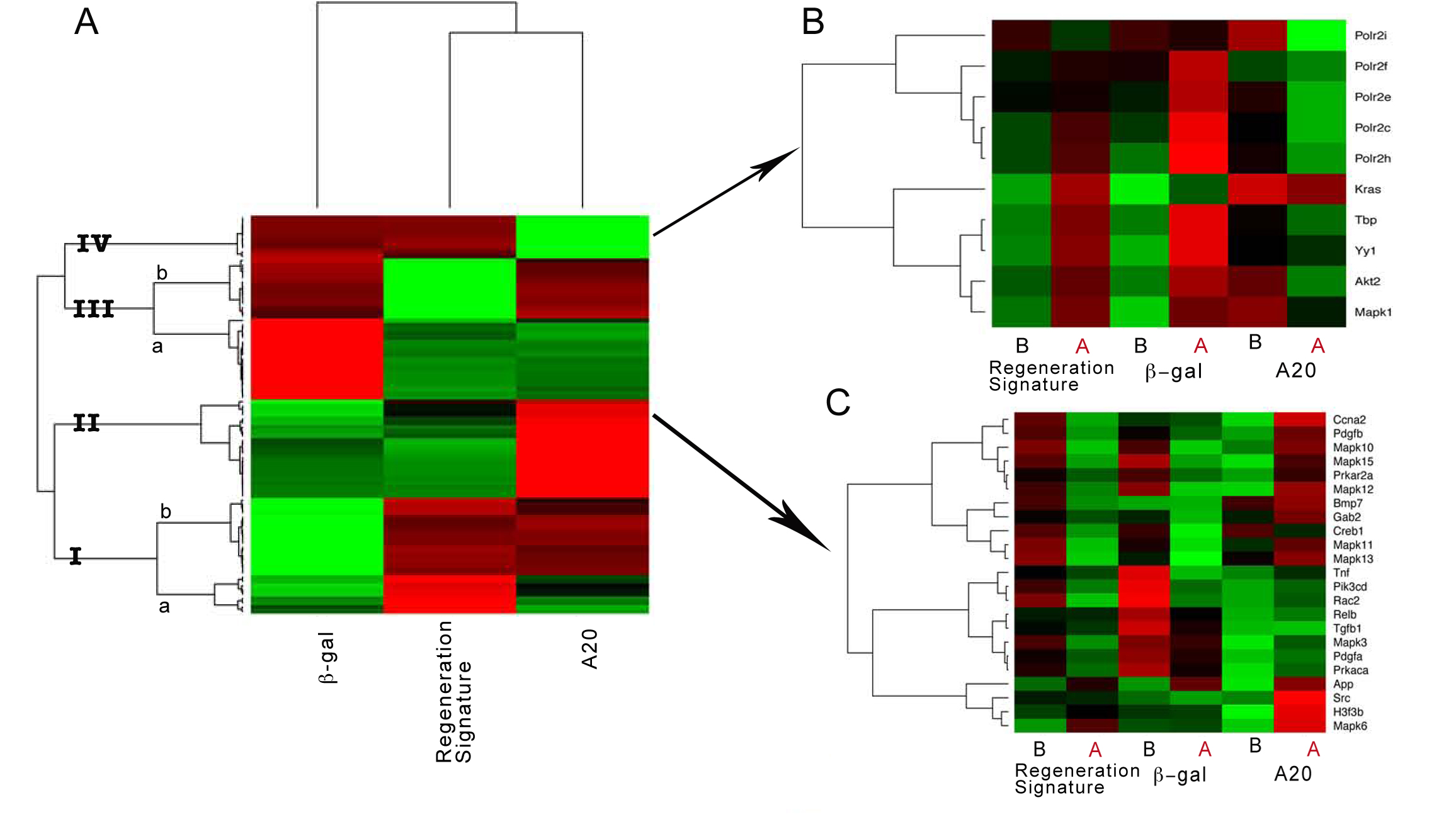

Supplement: Figure S5 — Validation of the role of A20 in liver regeneration by comparing with external transcriptional data. The behavior of 93 liver regeneration signature genes obtained from a previously published data set in mice were evaluated in rAd.A20 and rAd. βgal treated livers. The heat map depicts the LCB values of transcripts obtained by comparing before and after resection groups in the seed set (regeneration signature), the βgal and the A20 groups. The genes form four major clusters (I-IV) that consist of genes with different expression patterns. The columns represent the βgal, A20 and regeneration signature sets and the rows represent the 93 genes that are modified upon LR. Gene expression is shown with pseudocolor scale (−3 to 3) with red denoting high LCB (gene up regulation) and green denoting low LCB (gene down regulation). B) Subset of genes involved in regeneration that are upregulated by A20 even prior to resection. C) Subset of genes involved in regeneration that are down regulated by A20 even prior to resection. (TIF) [file pone.0017715.s005.tif]
