# Supplementary material for: A20 Modulates Lipid Metabolism and Energy Production to Promote Liver Regeneration
Source: PLoS One. 2011 Mar 17;6(3):e17715. doi: 10.1371/journal.pone.0017715 (PMC3060102; doi:10.1371/journal.pone.0017715)
Supplement: Table S1 — List of primers used in real-time PCR. (PDF) [file pone.0017715.s006.pdf]

Definition	locus	Forward	Reverse
Homo sapiens tumor necrosis factor, alpha-induced protein 3 (TNFAIP3)	NM_006290	AGTCCCACAGCGTCCAG	GCTCGATCTCAGTTGCTCT
Mus musculus peroxisome proliferator activated receptor alpha (Ppara)	NM_011144<br>NM_001113418	CGCCAGCACGGACGAGT	AGCCCTTACAGCCTTCACAT
Mus musculus cyclin-dependent kinase inhibitor 1A (P21) (Cdkn1a)	NM_001111099<br>NM_007669	ATCCTGGTGATGTCCGACCT	CGAAGTCAAAGTTCCACCGTT
Mus musculus actin, beta (Actb)	NM_007393	GGGAAATCGTGCGTGACAT	AAGGAAGGCTGGAAAAGAG
Mus musculus leptin receptor (Lepr)	NM_146146<br>NM_010704<br>NM_001122899	TGAGGAGGTACGTGGTGAAGCATC	CCGAGGGAATTGACAGCCAGAACT
beta-D-galactosidase [Escherichia coli BL21(DE3)]	ECD_00298	AGGCCACGGCGCTAATCACGA	GCTCCGCCGCCTTCATACTGC
Mus musculus solute carrier family 25 (mitochondrial carrier, adenine nucleotide translocator), member 13 (Slc25a13), nuclear gene encoding mitochondrial protein	NM_001177572<br>NM_015829	CCTGCGGCATCTTTAGTGACC	AATGCTTTGGGGCCCTCTTCTC
Mus musculus solute carrier family 25 (mitochondrial carrier, dicarboxylate transporter), member 10 (Slc25a10), nuclear gene encoding mitochondrial protein	NM_013770	GCCCCTCGATGTGCTGAAGAC	GATGCCCGCGGGAAAGAGACC
Mus musculus ubiquitin D (Ubd)	NM_023137	GCCCCATCGAAAATTGTCATCCTA	GTGCCTTTGCCCCTCGTTTTTG
