# Supplementary material for: A20 Modulates Lipid Metabolism and Energy Production to Promote Liver Regeneration
Source: PLoS One. 2011 Mar 17;6(3):e17715. doi: 10.1371/journal.pone.0017715 (PMC3060102; doi:10.1371/journal.pone.0017715)
Supplement: Table S2 — List of 1,595 unique genes, whose expression is uniquely affected by A20. The genes were identified in any of following comparison: i) βgal after vs. before resection, ii) A20 after vs. before resection, iii) A20 before resection vs. βgal before resection, or iv) A20 after resection vs. βgal after resection. The gene list was divided in 15 sections on the basis of the Venn diagram shown in Fig. S2. The table depicts the average expression level of the gene in A20 before and after resection as well as βgal before and after resection groups. (PDF) [file pone.0017715.s007.pdf]

Supplementary Table2: List of 1,595 unique genes, whose expression is uniquely affected by A20

Location	Entrez ID	Symbol	Gene Name	rAd.β-gal before	rAd.β-gal after	rAd.A20 before	rAd.A20 after
**q1**	13056	Cyb561	cytochrome b-561	82.48905	268.7655	36.32355	157.937
	18746	Pkm2	pyruvate kinase, muscle	807.015	1481.99	344.7825	800.6425
	18787	Serpine1	serine (or cysteine) peptidase inhibitor, clade E, member 1	648.4485	2973.5	230.418	954.0115
	226143	Cyp2c44	cytochrome P450, family 2, subfamily c, polypeptide 44	466.866	189.812	940.113	411.0415
	26877	B3galt1	UDP-Gal:betaGlcNAc beta 1,3-galactosyltransferase, polypeptide 1	79.77345	401.558	169.113	83.77705
	27400	Hsd17b6	hydroxysteroid (17-beta) dehydrogenase 6	1828.165	527.58	4075.455	1696.065
	27528	D0H4S114	DNA segment, human D4S114	197.627	65.4549	1196.401	154.9695
	57436	Gabarapl1	gamma-aminobutyric acid (GABA) A receptor-associated protein-like 1	1246.82	683.453	2656.34	1433.21
	72747	Ttc39c	tetratricopeptide repeat domain 39C	279.803	128.3875	871.9385	314.8
	75292	Prkd3	protein kinase D3	107.1161	51.2866	431.6705	150.263
	76654	Upp2	uridine phosphorylase 2	459.011	22.10945	2310.63	423.546
**q2**	11853	Rhoc	ras homolog gene family, member C	350.8375	749.6655	188.797	263.4255
	14864	Gstm3	glutathione S-transferase, mu 3	1229.04	552.8335	363.088	331.5695
	208715	Hmgcs1	3-hydroxy-3-methylglutaryl-Coenzyme A synthase 1	54.987	220.798	28.68725	27.6828
	227059	Slc39a10	solute carrier family 39 (zinc transporter), member 10	109.0722	390.7625	320.479	171.0885
	230603	Ttc39a	tetratricopeptide repeat domain 39A	219.8365	552.199	117.6015	187.6475
	26876	Adh4	alcohol dehydrogenase 4 (class II), pi polypeptide	706.5635	319.8755	2544.78	1746.795
**q3**	100039008	Mup10	major urinary protein 10	168.555	72.52805	2073.81	262.9395
	109212	6720460F02Rik	RIKEN cDNA 6720460F02 gene	317.0505	313.274	1944.02	770.8305
	12305	Ddr1	discoidin domain receptor family, member 1	63.36235	132.765	9.95068	43.00645
	12319	Car8	carbonic anhydrase 8	407.7485	210.483	1198.13	517.111
	20202	S100a9	S100 calcium binding protein A9 (calgranulin B)	896.6625	1938.05	126.606	330.78
	209186	Acnat2	acyl-coenzyme A amino acid N-acyltransferase 2	947.4565	1430.525	323.398	841.7495
	223337	Ugt3a2	UDP glycosyltransferases 3 family, polypeptide A2	1203.038	907.593	3553.5	1976.42
	27223	Trp53bp1	transformation related protein 53 binding protein 1	25.6655	13.2831	152.1735	49.20355
	319800	C730048C13Rik	RIKEN cDNA C730048C13 gene	831.7875	308.9625	2216.145	905.942
	404195	Cyp2c54	cytochrome P450, family 2, subfamily c, polypeptide 54	1035.212	498.191	2599.355	1320.98
	66277	Klf15	Kruppel-like factor 15	754.626	485.717	1681.565	863.9205
	66967	Edem3	ER degradation enhancer, mannosidase alpha-like 3	171.951	137.7735	975.749	366.705
	97998	Depdc6	DEP domain containing 6	323.013	183.496	1091.82	550.718
**q4**	100637	N4bp2l1	NEDD4 binding protein 2-like 1	338.5035	137.8035	661.1775	144.2085
	13119	Cyp4a14	cytochrome P450, family 4, subfamily a, polypeptide 14	1017.586	2204.59	159.7955	1555.835
	15496	Hsd3b5	hydroxy-delta-5-steroid dehydrogenase, 3 beta- and steroid delta-isomerase 5	431.868	111.405	4294.335	334.289
	16819	Lcn2	lipocalin 2	2746.48	13883.7	351.065	12983.45
	16840	Lect1	leukocyte cell derived chemotaxin 1	94.83155	42.61385	416.337	63.60865
	17068	Ly6d	lymphocyte antigen 6 complex, locus D	598.2825	2771.27	234.9875	1439.907
	17750	Mt2	metallothionein 2	6449.605	15454.25	2055.025	12764.15
	20208	Saa1	serum amyloid A 1	5951.1	11711.95	1405.002	11586.25
	20209	Saa2	serum amyloid A 2	2499.535	14444.65	329.5725	12180.3
	234199	Fgl1	fibrinogen-like protein 1	5155.185	9805.215	2054.685	9660.875
	27273	Pdk4	pyruvate dehydrogenase kinase, isoenzyme 4	247.745	944.6285	85.5181	1111.681
	64697	Keg1	kidney expressed gene 1	1446.196	180.682	4611.84	898.2185
**q5**	102293	AW111846	expressed sequence AW111846	1844.915	365.457	2783.81	1411.355
	107272	Psat1	phosphoserine aminotransferase 1	148.159	447.5555	77.49855	200.5715
	12475	Cd14	CD14 antigen	554.733	5378.635	160.251	618.276
	12484	Cd24a	CD24a antigen	143.2635	1313.53	89.8323	325.7525
	13803	Enc1	ectodermal-neural cortex 1	456.4365	1046.801	212.0335	432.5075
	15493	Hsd3b2	hydroxy-delta-5-steroid dehydrogenase, 3 beta- and steroid delta-isomerase 2	519.1685	144.875	723.5195	340.5125
	15494	Hsd3b3	hydroxy-delta-5-steroid dehydrogenase, 3 beta- and steroid delta-isomerase 3	1822.39	277.3655	2584.045	1264.23
	16005	Igfals	insulin-like growth factor binding protein, acid labile subunit	1098.674	360.317	1803.88	801.579
	17387	Mmp14	matrix metallopeptidase 14 (membrane-inserted)	499.2555	869.8165	283.1135	515.3835
	18414	Osmr	oncostatin M receptor	131.0609	340.4765	77.65955	146.857
	19141	Lgmn	legumain	583.269	1568.16	384.789	725.418
	19735	Rgs2	regulator of G-protein signaling 2	186.8975	1016.796	121.7401	321.572
	20315	Cxcl12	chemokine (C-X-C motif) ligand 12	1253.19	481.3915	1968.995	1077.21
	236069	Gm13238	predicted gene 13238	128.0715	496.5085	61.06665	132.0825
	272428	Acsm5	acyl-CoA synthetase medium-chain family member 5	760.828	252.591	1210.99	600.364
	53972	Ngef	neuronal guanine nucleotide exchange factor	266.005	110.8375	421.4135	209.3275
	57752	Tacc2	transforming, acidic coiled-coil containing protein 2	590.8675	1397.12	356.8445	762.4485
	58804	Cdc42ep5	CDC42 effector protein (Rho GTPase binding) 5	304.6255	833.1025	142.6345	426.7745
	60525	Acss2	acyl-CoA synthetase short-chain family member 2	865.1135	261.388	1049.665	521.373
	67307	3110049J23Rik	RIKEN cDNA 3110049J23 gene	1135.17	547.648	1834.605	1032.266
	71145	Scara5	scavenger receptor class A, member 5 (putative)	301.684	1873.685	241.218	802.137
	73205	3110043O21Rik	RIKEN cDNA 3110043O21 gene	205.164	844.2715	217.1185	407.549
	73230	Bmper	BMP-binding endothelial regulator	117.1775	950.4425	66.42105	435.4035
	73710	Tubb2b	tubulin, beta 2B	36.89855	86.49675	18.30615	46.33415
	75761	Apol7a	apolipoprotein L 7a	1829.86	370.411	1736.11	886.889
	76487	Ppp1r3g	protein phosphatase 1, regulatory (inhibitor) subunit 3G	65.36865	138.9225	124.311	50.2704

Location	Entrez ID	Symbol	Gene Name	rAd.β-gal before	rAd.β-gal after	rAd.A20 before	rAd.A20 after
	83397	Akap12	A kinase (PRKA) anchor protein (gravin) 12	144.5885	713.2345	83.80195	290.1755
**q6**	102122	2310065K24Rik	RIKEN cDNA 2310065K24 gene	602.861	1911.36	470.445	1652.175
	103988	Gck	glucokinase	1973.59	886.361	1772.635	730.6045
	107766	Haao	3-hydroxyanthranilate 3,4-dioxygenase	4100.58	1692.865	4581.43	2359.475
	108030	Lin7a	lin-7 homolog A (C. elegans)	458.6525	245.5055	570.8845	317.828
	109901	Cela1	chymotrypsin-like elastase family, member 1	1769.52	817.886	1835.985	656.444
	112407	Egln3	EGL nine homolog 3 (C. elegans)	227.362	562.7955	193.122	438.2265
	114332	Lyve1	lymphatic vessel endothelial hyaluronan receptor 1	176.772	689.3755	126.9165	357.5135
	11504	Adamts1	a disintegrin-like and metallopeptidase (reprolysin type) with thrombospondin type 1 motif, 1	219.027	571.0735	145.117	326.2535
	11770	Fabp4	fatty acid binding protein 4, adipocyte	233.5	1680.135	187.4825	1227.81
	11808	Apoa4	apolipoprotein A-IV	3290.875	13133.25	2414.205	14651.3
	11827	Aqp2	aquaporin 2	248.8315	682.3355	198.3705	407.6075
	12352	Car5a	carbonic anhydrase 5a, mitochondrial	1221.045	251.8535	1301.08	454.047
	12585	Cdr2	cerebellar degeneration-related 2	125.7035	395.548	108.537	243.176
	12623	Ces1	carboxylesterase 1	1302.57	559.4525	1858	723.922
	12739	Cldn3	claudin 3	152.96	354.6285	96.6661	279.1465
	13032	Ctsc	cathepsin C	147.38	50.9839	181.097	31.959
	13195	Ddc	dopa decarboxylase	1900.535	753.1735	1738.955	908.1835
	13423	Dnase2a	deoxyribonuclease II alpha	2137.84	857.324	2006.365	901.5005
	13730	Emp1	epithelial membrane protein 1	41.54545	227.255	36.42825	116.4392
	140483	Hnmt	histamine N-methyltransferase	807.2555	342.0705	1246.605	472.879
	14229	Fkbp5	FK506 binding protein 5	970.668	2145.9	618.9425	1596.615
	14311	Cidec	cell death-inducing DFFA-like effector c	96.96925	1241.725	85.273	794.855
	14453	Gas2	growth arrest specific 2	961.156	314.9795	1236.3	549.861
	14778	Gpx3	glutathione peroxidase 3	277.341	863.628	229.2045	718.1215
	15199	Hebp1	heme binding protein 1	2364.8	1064.283	3741.075	1813.51
	15587	Hyal2	hyaluronoglucosaminidase 2	828.837	1959.755	707.544	1944.01
	15982	Ifrd1	interferon-related developmental regulator 1	274.5085	818.721	302.6405	717.4095
	16597	Klf12	Kruppel-like factor 12	198.2985	88.42665	242.1875	127.2215
	16600	Klf4	Kruppel-like factor 4 (gut)	62.08775	189.849	62.0079	319.086
	18073	Nid1	nidogen 1	323.2395	835.622	202.6815	551.9155
	18113	Nnmt	nicotinamide N-methyltransferase	776.7525	6456.515	393.3175	3018.79
	18405	Orm1	orosomucoid 1	5452.205	12099.1	5147.2	11411.85
	18406	Orm2	orosomucoid 2	1188.526	3724.28	402.095	5933.815
	18407	Orm3	orosomucoid 3	161.0585	532.701	88.51545	344.3475
	20195	S100a11	S100 calcium binding protein A11 (calgizzarin)	871.1355	2518.365	576.3655	2014.72
	20425	Shmt1	serine hydroxymethyltransferase 1 (soluble)	817.9695	434.7395	1118.69	653.473
	20716	Serpina3n	serine (or cysteine) peptidase inhibitor, clade A, member 3N	4365.55	13762.6	4601.93	10291.22
	211949	Spsb4	splA/ryanodine receptor domain and SOCS box containing 4	274.319	105.7964	332.1335	117.1565
	21743	Inmt	indolethylamine N-methyltransferase	4847.03	1904.54	7720.405	1472.127
	225182	Rbbp8	retinoblastoma binding protein 8	450.5905	776.1905	342.0615	641.747
	229521	Syt11	synaptotagmin XI	243.6075	653.7385	166.69	342.451
	234564	AU018778	expressed sequence AU018778	3492.895	1443.195	4645.11	1429.624
	246277	Csad	cysteine sulfinic acid decarboxylase	3914.905	1193.17	3183.61	1128.584
	27279	Tnfrsf12a	tumor necrosis factor receptor superfamily, member 12a	543.907	2421.675	367.823	1842.74
	319202	1200016E24Rik	RIKEN cDNA 1200016E24 gene	2424.935	7500.05	1324.45	4000.51
	320360	Ric3	resistance to inhibitors of cholinesterase 3 homolog (C. elegans)	34.18055	11.7816	44.08995	7.186
	326618	Tpm4	tropomyosin 4	840.357	1738.285	510.842	1141.219
	330401	Tmcc1	transmembrane and coiled coil domains 1	76.7349	235.043	62.34535	199.142
	380712	Tlcd2	TLC domain containing 2	1324.64	639.437	2017.565	778.605
	432720	Akr1c19	aldo-keto reductase family 1, member C19	674.8365	238.2385	865.9485	315.9465
	52633	Nit2	nitrilase family, member 2	1689.45	733.752	3350.885	1317.36
	57266	Cxcl14	chemokine (C-X-C motif) ligand 14	59.4308	235.7935	39.17995	190.1515
	57435	Plin4	perilipin 4	232.324	917.3465	183.2145	458.584
	58198	Sall1	sal-like 1 (Drosophila)	227.8565	904.815	151.392	959.602
	66116	Cml1	camello-like 1	3165.84	1032.844	4524.175	1531.9
	66451	2610528J11Rik	RIKEN cDNA 2610528J11 gene	200.7875	92.73155	335.506	141.873
	67017	2010011I20Rik	RIKEN cDNA 2010011I20 gene	544.7405	1099.81	724.8415	1809.805
	67039	Rbm25	RNA binding motif protein 25	394.9905	870.6585	352.1235	678.081
	67073	Pi4k2b	phosphatidylinositol 4-kinase type 2 beta	250.246	672.182	222.747	642.522
	67866	Wfdc1	WAP four-disulfide core domain 1	106.6189	512.182	141.93	704.597
	67951	Tubb6	tubulin, beta 6	645.336	1376.605	420.2515	1083.525
	68312	Gstm7	glutathione S-transferase, mu 7	1181.505	604.326	1064.715	595.3395
	68631	Cryl1	crystallin, lambda 1	434.214	230.8255	540.927	219.2645
	69716	Trip13	thyroid hormone receptor interactor 13	174.9415	337.035	161.348	328.7035
	69861	2010003K11Rik	RIKEN cDNA 2010003K11 gene	466.5465	1510.415	317.4745	869.129
	70408	Polr3f	polymerase (RNA) III (DNA directed) polypeptide F	207.345	413.012	236.5965	636.612
	71693	Colec11	collectin sub-family member 11	549.4435	211.998	737.9365	311.1355
	71760	Agxt2l1	alanine-glyoxylate aminotransferase 2-like 1	1390.21	553.9295	1768.96	909.4285
	71921	2310058N22Rik	RIKEN cDNA 2310058N22 gene	78.75165	197.991	73.42555	177.0675
	72007	Fndc3b	fibronectin type III domain containing 3B	526.751	1324.62	548.723	1126.11
	72033	Tsc22d2	TSC22 domain family, member 2	229.164	632.199	260.2485	528.293
	75137	Rprd2	regulation of nuclear pre-mRNA domain containing 2	67.7565	137.8605	90.7091	229.925
	75178	4930528F23Rik	RIKEN cDNA 4930528F23 gene	256.397	135.533	340.521	159.601
	75985	Rab30	RAB30, member RAS oncogene family	114.2398	672.7755	188.8735	1096.098
	76574	Mfsd2	major facilitator superfamily domain containing 2	328.5625	3122.835	701.254	3058.515
	76905	Lrg1	leucine-rich alpha-2-glycoprotein 1	6231.08	13215.8	5086.725	13330.85
	77097	Tanc2	tetratricopeptide repeat, ankyrin repeat and coiled-coil containing 2	107.8075	263.7175	75.51255	174.555

Location	Entrez ID	Symbol	Gene Name	rAd.β-gal before	rAd.β-gal after	rAd.A20 before	rAd.A20 after
	77579	Myh10	myosin, heavy polypeptide 10, non-muscle	407.5745	824.3595	298.12	572.8485
**q7**	101565	6330503K22Rik	RIKEN cDNA 6330503K22 gene	444.705	769.435	202.7485	286.1765
	11867	Arpc1b	actin related protein 2/3 complex, subunit 1B	1001.413	1212.76	430.8115	486.184
	12355	Nr1i3	nuclear receptor subfamily 1, group I, member 3	658.6085	300.4235	1462.16	1321.285
	12447	Ccne1	cyclin E1	1164.099	1669.08	455.5875	642.1485
	12575	Cdkn1a	cyclin-dependent kinase inhibitor 1A (P21)	1295.38	1911.32	290.771	357.597
	13123	Cyp7b1	cytochrome P450, family 7, subfamily b, polypeptide 1	1840.825	2168.375	5531.18	3992.705
	16841	Lect2	leukocyte cell-derived chemotaxin 2	1218.155	1503.695	2967.25	4284.97
	16880	Lifr	leukemia inhibitory factor receptor	694.1	457.45	1576.635	1109.955
	16922	Phyh	phytanoyl-CoA hydroxylase	2902.47	2697.045	6866.92	5234.765
	170942	Erdr1	erythroid differentiation regulator 1	2062.155	2148.12	821.901	1115.235
	17769	Mthfr	5,10-methylenetetrahydrofolate reductase	417.345	569.0465	168.6055	253.619
	19118	Prm1	protamine 1	2011.61	1193.01	10.17975	11.68465
	20201	S100a8	S100 calcium binding protein A8 (calgranulin A)	764.9695	1735.65	161.85	361.1725
	20555	Slfn1	schlafen 1	122.5735	196.7215	56.68305	74.3125
	20558	Slfn4	schlafen 4	239.9135	486.678	91.08025	152.252
	226691	AI607873	expressed sequence AI607873	155.379	187.792	57.4467	85.9997
	268756	Gulo	gulonolactone (L-) oxidase	1608.9	916.66	3285.52	3501.485
	27280	Phlda3	pleckstrin homology-like domain, family A, member 3	1505.72	1873.74	438.5265	590.3925
	54446	Nfat5	nuclear factor of activated T-cells 5	93.97595	89.96295	320.226	184.8225
	67432	Dhdpsl	dihydrodipicolinate synthase-like, mitochondrial	646.2665	392.2695	1333.095	774.5005
	68234	2400009B08Rik	RIKEN cDNA 2400009B08 gene	227.44	260.1545	100.1146	138.5165
	68774	Ms4a6d	membrane-spanning 4-domains, subfamily A, member 6D	395.1855	664.189	132.0665	191.4635
	73681	Trmt11	tRNA methyltransferase 11 homolog (S. cerevisiae)	202.275	210.544	513.904	371.0185
	73866	Fam122c	family with sequence similarity 122, member C	120.685	101.2577	431.553	255.675
	74442	Sgms2	sphingomyelin synthase 2	255.8425	210.111	523.1375	547.5725
	77300	Raph1	Ras association (RalGDS/AF-6) and pleckstrin homology domains 1	1469.935	2106.23	684.619	917.1155
	77440	9430087N24Rik	RIKEN cDNA 9430087N24 gene	52.6857	47.7088	232.6315	95.0201
	80515	A030009H04Rik	RIKEN cDNA A030009H04 gene	123.773	170.909	64.4363	58.09545
**q8**	100037258	Dnajc3	DnaJ (Hsp40) homolog, subfamily C, member 3	318.925	762.451	703.5605	601.62
	100702	Mpa2l	macrophage activation 2 like	3278.56	885.154	1621	1654.724
	103142	Rdh9	retinol dehydrogenase 9	368.386	828.2495	946.5695	2053.36
	11655	Alas1	aminolevulinic acid synthase 1	991.136	2272.215	3736.58	3198.76
	11826	Aqp1	aquaporin 1	744.611	1339.09	1222.69	1297.078
	13649	Egfr	epidermal growth factor receptor	408.481	1150.42	1495.255	1465.61
	14955	H19	H19 fetal liver mRNA	1719.884	136.9845	94.7635	116.3765
	15490	Hsd17b7	hydroxysteroid (17-beta) dehydrogenase 7	178.979	485.8435	472.4525	553.347
	15957	Ifit1	interferon-induced protein with tetratricopeptide repeats 1	1220.325	436.358	350.5775	510.9305
	16326	Inhbe	inhibin beta E	300.4095	761.633	558.986	593.6805
	16362	Irf1	interferon regulatory factor 1	3938.09	1711.98	1368.965	2252.52
	16912	Psmb9	proteasome (prosome, macropain) subunit, beta type 9 (large multifunctional peptidase 2)	4130.355	1774.72	1898.915	2285.1
	17386	Mmp13	matrix metallopeptidase 13	134.44	47.9682	44.9278	43.7831
	18457	Pldn	pallidin	172.4195	441.299	368.5135	335.197
	19165	Psen2	presenilin 2	3471.71	1174.18	1614.145	976.6865
	20714	Serpina3k	serine (or cysteine) peptidase inhibitor, clade A, member 3K	980.246	2415.805	3357.365	3755.965
	20846	Stat1	signal transducer and activator of transcription 1	557.4285	128.2395	77.78905	152.0158
	320500	Tmem215	transmembrane protein 215	111.452	56.9816	50.4888	60.40435
	330064	Slc5a6	solute carrier family 5 (sodium-dependent vitamin transporter), member 6	145.313	282.845	263.337	270.3085
	52357	Wwc2	WW, C2 and coiled-coil domain containing 2	71.4634	147.138	140.712	144.8735
	53412	Ppp1r3c	protein phosphatase 1, regulatory (inhibitor) subunit 3C	2391.74	608.3715	931.788	864.858
	54598	Calcrl	calcitonin receptor-like	223.732	122.874	94.3949	126.3775
	56066	Cxcl11	chemokine (C-X-C motif) ligand 11	409.8675	184.4635	169.0975	223.289
	58185	Rsad2	radical S-adenosyl methionine domain containing 2	1747.35	459.396	186.755	486.739
	633640	Gm7120	predicted gene 7120	147.9135	300.964	315.606	207.3575
	66234	Sc4mol	sterol-C4-methyl oxidase-like	797.734	2284.58	2436.51	1517.02
	66729	4921520G13Rik	RIKEN cDNA 4921520G13 gene	377.2705	224.671	182.859	206.0725
	74182	Prei4	preimplantation protein 4	321.515	918.19	1329.06	949.354
**q9**	104771	1200003C05Rik	RIKEN cDNA 1200003C05 gene	543.7675	567.557	506.8785	287.287
	105387	Akr1c14	aldo-keto reductase family 1, member C14	890.7695	242.2245	2668.46	1043.465
	110956	D17H6S56E-5	DNA segment, Chr 17, human D6S56E 5	628.543	785.8295	600.9725	2283.715
	11674	Aldoa	aldolase A, fructose-bisphosphate	1824.28	3735.33	754.0895	1571.015
	12648	Chd1	chromodomain helicase DNA binding protein 1	176.162	222.1925	169.5525	426.027
	13122	Cyp7a1	cytochrome P450, family 7, subfamily a, polypeptide 1	211.8906	75.39125	524.6	185.1455
	13433	Dnmt1	DNA methyltransferase (cytosine-5) 1	403.158	582.2075	142.751	295.2165
	15366	Hmmr	hyaluronan mediated motility receptor (RHAMM)	161.987	204.8945	211.169	413.0975
	15382	Hnrnpa1	heterogeneous nuclear ribonucleoprotein A1	322.834	648.25	207.0655	370.952
	15937	Ier3	immediate early response 3	415.079	1091.066	99.3266	223.109
	16551	Kif11	kinesin family member 11	162.3265	186.2295	184.7715	356.068
	171210	Acot2	acyl-CoA thioesterase 2	926.944	1709.67	456.446	887.7815
	17190	Mbd1	methyl-CpG binding domain protein 1	322.171	536.0025	520.6845	136.8595
	17222	Anapc1	anaphase promoting complex subunit 1	93.1342	195.291	100.7737	404.317
	18005	Nek2	NIMA (never in mitosis gene a)-related expressed kinase 2	293.0955	341.2845	327.938	583.7235
	18024	Nfe2l2	nuclear factor, erythroid derived 2, like 2	85.773	103.4785	118.215	252.942

Location	Entrez ID	Symbol	Gene Name	rAd.β-gal before	rAd.β-gal after	rAd.A20 before	rAd.A20 after
	19252	Dusp1	dual specificity phosphatase 1	1813.74	1794.96	2151.8	1078.915
	19734	Rgs16	regulator of G-protein signaling 16	493.5295	309.7435	977.26	113.4317
	21807	Tsc22d1	TSC22 domain family, member 1	1142.335	841.4065	1376.01	453.8875
	21847	Klf10	Kruppel-like factor 10	289.3625	545.3055	401.257	185.721
	229759	Olfm3	olfactomedin 3	47.3619	20.1728	97.93215	43.03105
	55978	Ift20	intraflagellar transport 20 homolog (Chlamydomonas)	451.594	449.38	412.711	159.4385
	57875	Angptl4	angiopoietin-like 4	18.35915	22.14635	20.7783	64.93905
	64705	Dpys	dihydropyrimidinase	679.092	228.511	1238.295	584.1445
	71988	Esco2	establishment of cohesion 1 homolog 2 (S. cerevisiae)	124.1653	117.0113	160.5465	330.6435
	72027	Slc39a4	solute carrier family 39 (zinc transporter), member 4	1318.065	1090.265	774.626	440.1625
	78894	Aacs	acetoacetyl-CoA synthetase	464.2385	479.259	626.0665	198.746
**q10**	101613	Nlrp6	NLR family, pyrin domain containing 6	1049.016	430.586	1065.467	623.7675
	102123	9130221J18Rik	RIKEN cDNA 9130221J18 gene	265.293	612.8465	394.318	341.425
	103694	Tmed4	transmembrane emp24 protein transport domain containing 4	1466.8	2625.15	1833.185	2544.035
	104112	Acly	ATP citrate lyase	3154.03	1497.825	2072.535	1218.825
	104303	Arl1	ADP-ribosylation factor-like 1	2284.96	3686.81	3605.825	2996.58
	104662	Tsr1	TSR1, 20S rRNA accumulation, homolog (yeast)	304.193	613.922	240.398	350.364
	105148	Iars	isoleucine-tRNA synthetase	528.0735	1101.5	683.563	744.428
	106200	Txndc11	thioredoxin domain containing 11	263.7	491.4465	285.293	330.92
	108682	Gpt2	glutamic pyruvate transaminase (alanine aminotransferase) 2	1512.025	3678.825	1691.795	2218.71
	109222	Rarres1	retinoic acid receptor responder (tazarotene induced) 1	1580.93	3364.885	2389.455	1976.675
	109801	Glo1	glyoxalase 1	1523.005	528.0065	2026.705	1135.319
	111175	Pecr	peroxisomal trans-2-enoyl-CoA reductase	3495.23	1562.58	3937.675	2752.81
	11576	Afp	alpha fetoprotein	69.68985	479.648	13.76185	660.6816
	117167	Steap4	STEAP family member 4	3303.19	7316.63	2005.095	4335.515
	11745	Anxa3	annexin A3	140.857	233.8285	106.7206	167.366
	11750	Anxa7	annexin A7	360.0875	630.223	615.068	589.939
	11891	Rab27a	RAB27A, member RAS oncogene family	334.3765	180.611	234.4855	198.4505
	11988	Slc7a2	solute carrier family 7 (cationic amino acid transporter, y+ system), member 2	377.6275	895.204	535.796	1255.339
	12033	Bcap29	B-cell receptor-associated protein 29	196.079	476.9945	374.889	356.369
	12140	Fabp7	fatty acid binding protein 7, brain	139.676	310.226	93.88695	171.8815
	12350	Car3	carbonic anhydrase 3	281.097	31.76385	513.201	98.4488
	12572	Cdk7	cyclin-dependent kinase 7 (homolog of Xenopus MO15 cdk-activating kinase)	270.722	506.592	410.6475	330.883
	12606	Cebpa	CCAAT/enhancer binding protein (C/EBP), alpha	2616.145	1200.865	4388.9	1715.49
	12608	Cebpb	CCAAT/enhancer binding protein (C/EBP), beta	1106.89	1978.835	1330.113	1422.285
	12662	Chm	choroidermia	173.0865	328.5575	265.369	229.917
	13108	Cyp2g1	cytochrome P450, family 2, subfamily g, polypeptide 1	333.35	78.34965	323.0505	173.387
	13494	Drg1	developmentally regulated GTP binding protein 1	639.275	1224.51	1020.315	846.589
	13518	Dst	dystonin	128.5995	267.2395	146.7425	245.93
	14012	Mpzl2	myelin protein zero-like 2	582.0925	1350.1	744.4245	1008.974
	14131	Fcgr3	Fc receptor, IgG, low affinity III	369.7435	700.096	270.5905	353.3555
	14377	G6pc	glucose-6-phosphatase, catalytic	3698.435	1841.82	4299.345	2945.835
	14455	Gas5	growth arrest specific 5	150.003	370.1875	106.8491	182.606
	14560	Gdf10	growth differentiation factor 10	140.871	277.4865	172.079	246.636
	14585	Gfra1	glial cell line derived neurotrophic factor family receptor alpha 1	685.991	1361.81	782.492	1226.15
	14618	Gjb1	gap junction protein, beta 1	4917.35	2527.68	5374.745	3924.37
	14859	Gsta3	glutathione S-transferase, alpha 3	971.2095	251.326	1229.944	521.036
	14860	Gsta4	glutathione S-transferase, alpha 4	2658.475	1389.77	2650.01	1235.918
	15107	Hadh	hydroxyacyl-Coenzyme A dehydrogenase	8804.675	4835.13	9452.31	7794.06
	15384	Hnrnpab	heterogeneous nuclear ribonucleoprotein A/B	423.4075	752.0915	388.85	553.995
	15959	Ifit3	interferon-induced protein with tetratricopeptide repeats 3	1198.355	519.652	680.723	696.3955
	16164	Il13ra1	interleukin 13 receptor, alpha 1	121.5845	269.639	163.6315	256.044
	16173	Il18	interleukin 18	892.006	491.1135	825.1325	628.8905
	16177	Il1r1	interleukin 1 receptor, type I	401.909	1389.335	481.713	946.851
	16475	Jub	ajuba	173.818	380.6305	164.704	356.477
	16542	Kdr	kinase insert domain protein receptor	712.959	346.6245	703.195	394.6185
	16573	Kif5b	kinesin family member 5B	207.9405	507.252	289.647	378.72
	16598	Klf2	Kruppel-like factor 2 (lung)	119.9175	253.107	119.5222	144.622
	16709	Ktn1	kinectin 1	245.109	641.843	236.936	297.2835
	16866	Lhb	luteinizing hormone beta	247.47	451.5585	270.347	323.388
	16978	Lrrfip1	leucine rich repeat (in FLII) interacting protein 1	328.667	804.268	339.5755	624.497
	17035	Lxn	latexin	275.456	535.205	229.9135	315.019
	170760	Acbd3	acyl-Coenzyme A binding domain containing 3	405.4995	722.002	450.386	661.364
	17217	Mcm4	minichromosome maintenance deficient 4 homolog (S. cerevisiae)	680.499	1188.385	391.4665	910.411
	17388	Mmp15	matrix metallopeptidase 15	613.908	347.381	535.636	393.367
	18082	Nipsnap1	4-nitrophenylphosphatase domain and non-neuronal SNAP25-like protein homolog 1 (C. elegans)	3094.81	1771.105	4027.185	2915.025
	18105	Nqo2	NAD(P)H dehydrogenase, quinone 2	868.3665	393.114	1042.875	734.3155
	18141	Nup50	nucleoporin 50	398.689	767.986	337.6245	585.713
	18148	Npm1	nucleophosmin 1	1388.47	2957.25	1906.73	1935.475
	18585	Pde9a	phosphodiesterase 9A	376.2555	206.2825	299.482	235.162
	18704	Pik3c2a	phosphatidylinositol 3-kinase, C2 domain containing, alpha polypeptide	216.747	641.826	327.352	386.065
	18984	Por	P450 (cytochrome) oxidoreductase	1893.725	3682.995	2619.94	4754.715
	19012	Ppap2a	phosphatidic acid phosphatase type 2A	554.2115	1018.278	615.2375	854.2175
	19043	Ppm1b	protein phosphatase 1B, magnesium dependent, beta isoform	849.7015	363.906	570.579	412.3125
	19116	Prlr	prolactin receptor	498.5285	227.833	276.811	245.601
	19126	Prom1	prominin 1	68.1704	164.628	73.23375	103.8975

Location	Entrez ID	Symbol	Gene Name	rAd.β-gal before	rAd.β-gal after	rAd.A20 before	rAd.A20 after
	192657	Ell2	elongation factor RNA polymerase II 2	594.7155	1254.635	1102.765	950.685
	19266	Ptprd	protein tyrosine phosphatase, receptor type, D	695.682	287.6805	845.411	502.8185
	19277	Ptpro	protein tyrosine phosphatase, receptor type, O	48.80235	162.718	49.70085	88.0007
	19301	Pxmp2	peroxisomal membrane protein 2	2968.86	1541.89	2991.415	2248.435
	19324	Rab1	RAB1, member RAS oncogene family	618.611	1156.935	926.724	1022.034
	19356	Rad17	RAD17 homolog (S. pombe)	317.4475	689.9875	435.743	495.2695
	19416	Rasd1	RAS, dexamethasone-induced 1	100.9889	292.0865	93.32175	187.501
	19652	Rbm3	RNA binding motif protein 3	1242.19	3740.955	1814.175	2752.455
	20148	Dhrs3	dehydrogenase/reductase (SDR family) member 3	1696.205	426.324	1478.53	822.3105
	20219	Apcs	serum amyloid P-component	5216.25	9460.815	5865.355	10009.29
	20454	St3gal5	ST3 beta-galactoside alpha-2,3-sialyltransferase 5	1409.54	3494.78	1569.68	2657.25
	20498	Slc12a4	solute carrier family 12, member 4	309.2915	621.4695	298.4225	376.839
	20509	Slc19a1	solute carrier family 19 (sodium/hydrogen exchanger), member 1	231.507	605.578	273.6305	426.8
	20520	Slc22a5	solute carrier family 22 (organic cation transporter), member 5	409.179	1208.58	595.5335	836.544
	20729	Spin1	spindlin 1	724.581	1507.785	993.0275	1035.925
	20740	Spna2	spectrin alpha 2	637.7465	1219.13	518.981	880.4335
	20742	Spnb2	spectrin beta 2	1162.575	2024.005	1118.82	1652.305
	20773	Sptlc2	serine palmitoyltransferase, long chain base subunit 2	516.4845	941.3125	416.5885	590.812
	20775	Sqle	squalene epoxidase	638.6435	2160.94	1022.574	1399.155
	20787	Srebf1	sterol regulatory element binding transcription factor 1	3246.08	1053.005	2711.47	1802.825
	20810	Srm	spermidine synthase	783.249	1693.96	987.6025	1025.175
	208449	Sgms1	sphingomyelin synthase 1	306.923	1030.036	287.2775	639.8435
	20860	Sult1e1	sulfotransferase family 1E, member 1	45.8426	480.434	44.52775	231.8722
	211548	Nomo1	nodal modulator 1	206.086	529.301	209.884	295.506
	213053	Slc39a14	solute carrier family 39 (zinc transporter), member 14	387.937	952.277	417.274	738.381
	21376	Tbrg1	transforming growth factor beta regulated gene 1	1105.11	2536.575	1153.64	1520.155
	21401	Tcea3	transcription elongation factor A (SII), 3	621.926	145.259	588.5205	285.737
	214498	Cdc73	cell division cycle 73, Paf1/RNA polymerase II complex component, homolog (S. cerevisiae)	264.5755	483.8145	318.464	321.0035
	21580	Tcrb-J	T-cell receptor beta, joining region	56.56185	114.988	58.7108	90.50665
	216456	Gls2	glutaminase 2 (liver, mitochondrial)	1652.855	795.488	1949.695	1230.01
	21824	Thbd	thrombomodulin	50.27855	592.2465	52.9783	217.5795
	21854	Timm17a	translocase of inner mitochondrial membrane 17a	833.94	1740.16	1119.03	1095.65
	22042	Tfrc	transferrin receptor	2023.535	1169.665	1305.858	1211.7
	22200	Uba3	ubiquitin-like modifier activating enzyme 3	342.7835	653.1455	463.978	426.748
	22228	Ucp2	uncoupling protein 2 (mitochondrial, proton carrier)	1155.075	2116.575	777.5085	1449.47
	22234	Ugcg	UDP-glucose ceramide glucosyltransferase	325.7705	645.3705	314.047	524.8445
	22350	Ezr	ezrin	134.58	286.468	110.969	164.137
	22433	Xbp1	X-box binding protein 1	1337.366	4105.755	3007.605	3578.4
	224796	Clic5	chloride intracellular channel 5	115.028	56.0745	109.1819	99.82055
	226418	Yod1	YOD1 OTU deubiquitinating enzyme 1 homologue (S. cerevisiae)	182.766	353.334	220.773	214.1445
	226442	Zfp281	zinc finger protein 281	202.7105	474.9615	260.2095	307.195
	226856	Lpgat1	lysophosphatidylglycerol acyltransferase 1	840.779	2307.265	1070.159	1528.555
	227399	Hisppd1	histidine acid phosphatase domain containing 1	143.15	317.3895	204.0915	269.321
	22750	Zfp9	zinc finger protein 9	164.976	317.8285	197.458	278.4375
	229731	Slc25a24	solute carrier family 25 (mitochondrial carrier, phosphate carrier), member 24	67.1467	149.2685	52.7973	106.6128
	230721	Pabpc4	poly(A) binding protein, cytoplasmic 4	690.286	1311.01	816.923	1129.365
	230979	Tnfrsf14	tumor necrosis factor receptor superfamily, member 14 (herpesvirus entry mediator)	206.8805	101.9054	146.1465	143.309
	231287	Atp10d	ATPase, class V, type 10D	335.0725	705.343	288.031	526.0875
	231474	Paqr3	progestin and adipoQ receptor family member III	102.3259	242.9985	188.417	248.375
	232086	Tmem150	transmembrane protein 150	1976.625	927.32	1916.675	1330.82
	233489	Picalm	phosphatidylinositol binding clathrin assembly protein	1579.72	3203.98	1575.955	2312.825
	234463	Tmem184c	transmembrane protein 184C	165.907	454.212	283.003	371.002
	235459	Gtf2a2	general transcription factor II A, 2	963.0635	2404.93	1346.985	1439.05
	236792	Mmgt1	membrane magnesium transporter 1	561.2115	1015.989	738.9365	700.7245
	23796	Aplnr	apelin receptor	90.0854	226.3195	76.37995	149.5375
	23831	Car14	carbonic anhydrase 14	1039.103	571.2735	779.107	685.4965
	23986	Peci	peroxisomal delta3, delta2-enoyl-Coenzyme A isomerase	3131.485	5583.255	4477.345	5576.21
	240255	Ythdc2	YTH domain containing 2	71.25655	187.733	100.2834	116.526
	243085	Ugt2b35	UDP glucuronosyltransferase 2 family, polypeptide B35	4078.405	2343.955	4927.645	3194.83
	244198	Olfml1	olfactomedin-like 1	219.1225	126.6005	260.3495	145.7265
	244416	Ppp1r3b	protein phosphatase 1, regulatory (inhibitor) subunit 3B	2245.465	1096.76	2600.925	1277.385
	252838	Tox	thymocyte selection-associated high mobility group box	113.924	296.129	136.2655	208.3875
	26409	Map3k7	mitogen-activated protein kinase kinase kinase 7	244.8455	453.2335	321.453	327.9095
	26456	Sema4g	sema domain, immunoglobulin domain (Ig), transmembrane domain (TM) and short cytoplasmic domain, (semaphorin) 4G	2661.25	1226.63	2330.09	2089.53
	269261	Rpl12	ribosomal protein L12	131.3915	280.0935	93.1658	151.623
	27973	Vkorc1	vitamin K epoxide reductase complex, subunit 1	5591.84	3122.405	6314.555	4744.36
	30877	Gnl3	guanine nucleotide binding protein-like 3 (nucleolar)	741.3075	1435.15	946.16	980.9215
	30956	Aass	aminoadipate-semialdehyde synthase	1018.639	2728.565	1995.415	2999.505
	319269	A130040M12Rik	RIKEN cDNA A130040M12 gene	132.788	339.436	144.9618	279.4345
	319625	Galm	galactose mutarotase	1466.525	660.3185	1913.94	1055.985
	319876	Cobll1	Cobl-like 1	509.4305	874.9325	766.646	1015.533
	320024	Nceh1	arylacetamide deacetylase-like 1	127.718	336.45	174.056	217.1805
	320051	Exph5	exophilin 5	172.5565	93.1806	180.8335	138.696
	320226	4930473A06Rik	RIKEN cDNA 4930473A06 gene	76.37945	147.61	77.1805	116.2205
	320817	Atad2b	ATPase family, AAA domain containing 2B	346.5785	612.8555	402.981	408.5585
	330122	Cxcl3	chemokine (C-X-C motif) ligand 3	49.05065	227.3325	46.1129	62.65385
	338467	Morc3	microrchidia 3	321.702	621.2045	330.903	350.6485

Location	Entrez ID	Symbol	Gene Name	rAd.β-gal before	rAd.β-gal after	rAd.A20 before	rAd.A20 after
	378425	Nlrp12	NLR family, pyrin domain containing 12	390.068	1260.37	728.955	871.657
	381038	Parl	presenilin associated, rhomboid-like	575.553	996.25	753.13	656.067
	381066	BC049807	cDNA sequence BC049807	51.1856	121.528	50.5081	89.28785
	399101	Snhg3	small nucleolar RNA host gene (non-protein coding) 3	299.8725	727.682	247.384	468.33
	408064	BC064078	cDNA sequence BC064078	63.1231	135.2625	63.12335	82.1885
	50768	Dlc1	deleted in liver cancer 1	406.093	195.267	394.517	297.8175
	50995	Uba2	ubiquitin-like modifier activating enzyme 2	463.8015	864.991	563.2085	594.67
	52033	Pbk	PDZ binding kinase	271.2865	488.0845	290.7395	702.801
	52615	Suz12	suppressor of zeste 12 homolog (Drosophila)	355.2595	703.166	442.2905	541.312
	52639	Wipi1	WD repeat domain, phosphoinositide interacting 1	74.8481	129.221	92.915	121.193
	52829	D4Bwg0951e	DNA segment, Chr 4, Brigham & Women's Genetics 0951 expressed	603.2015	1427.785	1029.257	1125.259
	52837	Tmx4	thioredoxin-related transmembrane protein 4	346.753	756.2335	638.5375	631.2225
	53627	Porcn	porcupine homolog (Drosophila)	108.19	238.6325	106.2872	156.4845
	54124	Cks1b	CDC28 protein kinase 1b	458.1305	838.6675	500.597	609.782
	54153	Rasa4	RAS p21 protein activator 4	108.49	421.1995	88.18245	213.767
	54353	Skap2	src family associated phosphoprotein 2	599.2675	1020.445	654.082	683.9975
	54391	Rfk	riboflavin kinase	769.0565	1393.255	951.5435	831.3175
	54610	Tbc1d8	TBC1 domain family, member 8	319.3025	633.558	298.751	405.818
	55961	Slc13a1	solute carrier family 13 (sodium/sulfate symporters), member 1	52.67495	21.7808	25.45175	34.13405
	55983	Pdzrn3	PDZ domain containing RING finger 3	105.5387	342.316	111.543	206.856
	56200	Ddx21	DEAD (Asp-Glu-Ala-Asp) box polypeptide 21	383.9195	934.962	357.56	623.3715
	56209	Gde1	glycerophosphodiester phosphodiesterase 1	1076.743	2250.915	1318.12	1464.775
	56298	Atl2	atlastin GTPase 2	1072.726	2249.075	1772.685	2327.525
	56398	1500003O03Rik	RIKEN cDNA 1500003O03 gene	873.092	1666.14	1318.515	1448.035
	56401	Lepre1	leprecan 1	135.14	256.599	134.5315	180.5935
	56403	Syncrip	synaptotagmin binding, cytoplasmic RNA interacting protein	118.0695	225.146	148.917	265.714
	56434	Tspan3	tetraspanin 3	244.194	520.3695	252.451	322.411
	56695	Pnkd	paroxysmal nonkinesiogenic dyskinesia	1634.795	717.914	1013.862	1121.94
	56878	Rbms1	RNA binding motif, single stranded interacting protein 1	635.2405	1174.075	593.9135	878.0415
	57278	Bcam	basal cell adhesion molecule	213.592	489.828	186.871	297.6685
	57874	Ptplad1	protein tyrosine phosphatase-like A domain containing 1	2880.845	5419.49	3228.75	4380.765
	58805	Mlxipl	MLX interacting protein-like	2750.35	917.4945	1981.72	1000.767
	59032	Ppp2r3c	protein phosphatase 2, regulatory subunit B'', gamma	276.7695	525.4125	416.8715	392.1175
	624219	Gm6484	predicted gene 6484	1392.18	371.629	1182.105	472.964
	65019	Rpl23	ribosomal protein L23	184.3135	351.27	221.665	228.9945
	66071	Ethe1	ethylmalonic encephalopathy 1	2139.655	1117.155	1930.905	1188.875
	66120	Fkbp11	FK506 binding protein 11	512.451	1276.687	933.26	720.2765
	66249	Pno1	partner of NOB1 homolog (S. cerevisiae)	466.148	1127.3	960.6385	881.529
	66270	Fam134b	family with sequence similarity 134, member B	178.1685	519.5485	177.9205	276.15
	66314	Tpd52l2	tumor protein D52-like 2	370.638	619.1925	527.7255	411.0625
	66396	Ccdc82	coiled-coil domain containing 82	257.565	462.535	243.7095	316.541
	66510	Rnf181	ring finger protein 181	1915.49	1093.47	1790.205	1446.74
	66578	2610039C10Rik	RIKEN cDNA 2610039C10 gene	212.073	367.8325	239.5475	350.2
	66583	Exosc1	exosome component 1	229.296	428.924	293.1535	260.436
	66663	Uba5	ubiquitin-like modifier activating enzyme 5	324.933	549.165	392.4675	400.279
	66676	Tmed7	transmembrane emp24 protein transport domain containing 7	243.099	513.4255	482.7595	444.4295
	66681	Pgm1	phosphoglucomutase 1	181.564	396.877	173.7915	290.262
	667597	BC023105	cDNA sequence BC023105	2063.705	481.0685	1063.104	1108.874
	66853	Pnpla2	patatin-like phospholipase domain containing 2	957.4895	1919.47	827.1825	1395.865
	66878	Riok3	RIO kinase 3 (yeast)	328.77	579.2145	525.7725	366.6155
	66913	Kdelr2	KDEL (Lys-Asp-Glu-Leu) endoplasmic reticulum protein retention receptor 2	822.627	1573.815	950.1615	1151.82
	66922	Rras2	related RAS viral (r-ras) oncogene homolog 2	575.1055	1321.325	602.4255	839.589
	66943	Pqlc1	PQ loop repeat containing 1	1333.015	706.8375	1427.985	824.9415
	66968	Plin5	perilipin 5	775.7885	1910.48	745.7575	1649.44
	67070	Lsm14a	LSM14 homolog A (SCD6, S. cerevisiae)	281.71	551.5115	365.6965	437.3385
	67242	Gemin6	gem (nuclear organelle) associated protein 6	323.734	598.945	394.8495	438.268
	67392	4833420G17Rik	RIKEN cDNA 4833420G17 gene	110.8725	265.1135	192.139	203.3065
	67722	4921517D21Rik	RIKEN cDNA 4921517D21 gene	98.1896	249.162	95.37495	184.7095
	67800	Dgat2	diacylglycerol O-acyltransferase 2	6325.645	3265.49	6562.74	5040.385
	67815	Sec14l2	SEC14-like 2 (S. cerevisiae)	3332.07	1674.47	3651.155	2623.695
	67856	Echdc3	enoyl Coenzyme A hydratase domain containing 3	1470.52	617.0245	1718.105	1076.55
	67862	2310033P09Rik	RIKEN cDNA 2310033P09 gene	187.5635	401.852	151.8165	358.6925
	67903	Gipc1	GIPC PDZ domain containing family, member 1	319.6255	533.325	333.171	331.3675
	68187	Fam135a	family with sequence similarity 135, member A	297.5615	535.8735	345.758	409.889
	68303	Fam114a1	family with sequence similarity 114, member A1	327.326	728.538	484.436	492.9945
	68705	Gtf2f2	general transcription factor IIF, polypeptide 2	222.0685	532.5895	339.373	378.2605
	68777	Tmem53	transmembrane protein 53	934.052	419.1065	674.582	523.9625
	68979	Nol11	nucleolar protein 11	77.84365	164.8205	85.00325	138.465
	69090	Ascc1	activating signal cointegrator 1 complex subunit 1	1187.435	619.8135	651.5145	589.9995
	69202	Ptms	parathymosin	6262.685	3107.115	5230.125	4256.89
	69219	Ddah1	dimethylarginine dimethylaminohydrolase 1	731.8335	327.592	707.509	455.9885
	69482	Nup35	nucleoporin 35	275.193	472.69	309.5625	311.7125
	69568	Vkorc1l1	vitamin K epoxide reductase complex, subunit 1-like 1	350.2305	681.365	606.75	573.2405
	69608	Sec24d	Sec24 related gene family, member D (S. cerevisiae)	504.134	1217.02	999.098	1035.392
	69635	Dapk1	death associated protein kinase 1	1016.675	575.2345	671.4645	674.774
	69638	Enho	energy homeostasis associated	746.629	342.2305	716.7645	390.8505
	69906	Slc25a32	solute carrier family 25, member 32	139.666	319.407	278.3215	266.496
	70047	Trnt1	tRNA nucleotidyl transferase, CCA-adding, 1	233.4	409.8055	314.577	256.7445
	70241	2810416A17Rik	RIKEN cDNA 2810416A17 gene	211.5805	129.552	153.257	186.6445
	70297	Gcc2	GRIP and coiled-coil domain containing 2	471.677	997.578	458.5985	664.2925

Location	Entrez ID	Symbol	Gene Name	rAd.β-gal before	rAd.β-gal after	rAd.A20 before	rAd.A20 after
	70349	Copb1	coatomer protein complex, subunit beta 1	625.224	1277.45	1192.24	1109.332
	70425	Csnk1g3	casein kinase 1, gamma 3	445.621	850.3365	725.873	766.3065
	70465	Wdr77	WD repeat domain 77	485.0695	973.8335	632.0615	531.93
	71375	Foxn3	forkhead box N3	657.7635	304.9145	581.7375	376.2725
	71704	Arhgef3	Rho guanine nucleotide exchange factor (GEF) 3	220.2455	483.9105	324.665	391.451
	71724	Aox3	aldehyde oxidase 3	2463.715	822.635	4870.645	1843.335
	71833	Dcaf7	DDB1 and CUL4 associated factor 7	179.957	314.567	200.7375	248.351
	71910	Ppapdc1b	phosphatidic acid phosphatase type 2 domain containing 1B	272.9435	961.3685	382.693	533.866
	71919	Rpap3	RNA polymerase II associated protein 3	223.3915	539.2415	235.1855	307.9115
	72056	1810055G02Rik	RIKEN cDNA 1810055G02 gene	553.4415	1812.31	980.1465	1766.955
	72477	Tmem87b	transmembrane protein 87B	150.8865	551.4265	234.712	454.603
	72479	Hsdl2	hydroxysteroid dehydrogenase like 2	528.801	1072.795	791.0005	678.4535
	72655	2810026P18Rik	RIKEN cDNA 2810026P18 gene	176.936	407.339	165.74	324.0355
	72662	Dis3	DIS3 mitotic control homolog (S. cerevisiae)	259.9515	599.931	280.343	358.768
	72810	2810455D13Rik	RIKEN cDNA 2810455D13 gene	95.01615	262.04	104.2889	214.585
	73130	Tmed5	transmembrane emp24 protein transport domain containing 5	667.524	2181.66	1343.415	1634.255
	74197	Gtf2e1	general transcription factor II E, polypeptide 1 (alpha subunit)	229.215	447.9015	307.5945	321.0195
	74245	Ctbs	chitobiase, di-N-acetyl-	198.73	371.521	267.994	287.6745
	74427	Eaf1	ELL associated factor 1	350.711	805.865	560.4665	647.5915
	74509	6820402I19Rik	RIKEN cDNA 6820402I19 gene	133.623	240.077	160.8905	137.954
	74754	Dhcr24	24-dehydrocholesterol reductase	1264.285	518.3585	624.1795	1263.883
	75015	4930503B20Rik	RIKEN cDNA 4930503B20 gene	197.9295	110.3895	250.637	245.276
	75316	Taf1d	TATA box binding protein (Tbp)-associated factor, RNA polymerase I, D	147.0825	270.695	188.663	258.197
	75812	Tasp1	taspase, threonine aspartase 1	26.9872	56.07875	43.10855	33.7783
	76080	Ttpal	tocopherol (alpha) transfer protein-like	156.698	314.4755	137.5415	188.4155
	76491	Abhd14b	abhydrolase domain containing 14b	3132.665	1516.485	3550.15	2325.37
	76522	Lsm8	LSM8 homolog, U6 small nuclear RNA associated (S. cerevisiae)	408.3245	849.2705	420.868	674.2065
	77037	Mrap	melanocortin 2 receptor accessory protein	921.907	3325.44	992.036	2117.345
	77113	Klhl2	kelch-like 2, Mayven (Drosophila)	83.87425	180.892	128.6845	101.6902
	77506	8030497O21Rik	RIKEN cDNA 8030497O21 gene	85.8305	207.5575	101.6055	134.2235
	77559	Agl	amylo-1,6-glucosidase, 4-alpha-glucanotransferase	506.373	279.902	793.6005	461.125
	78830	Slc25a12	solute carrier family 25 (mitochondrial carrier, Aralar), member 12	368.1995	727.5075	439.6035	608.676
	78889	Wsb1	WD repeat and SOCS box-containing 1	367.0715	678.333	462.716	404.775
	80289	Lysmd3	LysM, putative peptidoglycan-binding, domain containing 3	255.6915	583.162	449.294	446.186
	80914	Uck2	uridine-cytidine kinase 2	194.5875	414.9555	205.288	226.02
	81004	Tbl1xr1	transducin (beta)-like 1X-linked receptor 1	477.1455	1061.96	562.324	825.7965
	98238	Lrrc59	leucine rich repeat containing 59	1241.115	3679.025	1398.05	2068.81
	98417	Cnih4	cornichon homolog 4 (Drosophila)	400.397	770.362	569.8025	544.703
	98496	5033414K04Rik	RIKEN cDNA 5033414K04 gene	108.9642	230.155	113.742	140.3225
	99167	Ssx2ip	synovial sarcoma, X breakpoint 2 interacting protein	150.975	323.3905	152.4295	164.342
	99929	Tiparp	TCDD-inducible poly(ADP-ribose) polymerase	206.1425	415.7665	224.8055	292.578
**q11**	100494	Zfand2a	zinc finger, AN1-type domain 2A	142.066	163.087	96.2405	205.595
	100532	Rell1	RELT-like 1	778.069	741.802	277.953	669.086
	100952	Emilin1	elastin microfibril interfacer 1	288.504	379.21	208.62	369.8205
	101476	Plekha1	pleckstrin homology domain containing, family A (phosphoinositide binding specific) member 1	293.167	538.057	235.9765	387.6905
	102022	Ces6	carboxylesterase 6	3767.77	2676.64	5169.47	2024.825
	104009	Qsox1	quiescin Q6 sulfhydryl oxidase 1	1452.185	1912.01	1453.28	2625.615
	104027	Synpo	synaptopodin	168.005	357.174	82.1571	204.276
	104158	Ces3	carboxylesterase 3	4055.84	2036.34	5564.75	2385.265
	105670	Rcbtb2	regulator of chromosome condensation (RCC1) and BTB (POZ) domain containing protein 2	1343.765	1114.05	1230.2	563.0865
	107227	Macrod1	MACRO domain containing 1	576.7045	342.365	727.017	278.531
	108961	E2f8	E2F transcription factor 8	628.1445	935.889	430.5705	828.2955
	109169	6330403M23Rik	RIKEN cDNA 6330403M23 gene	203.103	194.7215	251.2455	144.913
	110033	Kif22	kinesin family member 22	145.4085	152.687	90.3118	163.0115
	110789	Gpr98	G protein-coupled receptor 98	388.027	261.949	575.653	299.466
	11535	Adm	adrenomedullin	153.5535	203.2335	125.638	277.167
	11569	Aebp2	AE binding protein 2	778.1415	1309.265	533.356	1051.06
	11641	Akap2	A kinase (PRKA) anchor protein 2	149.471	318.7205	83.8885	206.4015
	11747	Anxa5	annexin A5	1820.375	2590.7	1435.61	2752.975
	11801	Cd5l	CD5 antigen-like	1743.37	1278.055	797.801	1712
	11829	Aqp4	aquaporin 4	119.347	133.5485	307.8235	147.475
	11845	Arf6	ADP-ribosylation factor 6	1087.604	1629.16	811.229	1463.195
	11861	Arl4a	ADP-ribosylation factor-like 4A	226.805	281.105	144.8375	336.296
	12182	Bst1	bone marrow stromal cell antigen 1	81.77955	111.829	61.9702	107.4265
	12217	Bsn	bassoon	239.125	213.2525	375.6475	204.162
	12236	Bub1b	budding uninhibited by benzimidazoles 1 homolog, beta (S. cerevisiae)	191.6595	171.431	116.8875	224.5975
	12306	Anxa2	annexin A2	732.773	1494.955	321.953	1007.656
	12401	Serpina6	serine (or cysteine) peptidase inhibitor, clade A, member 6	2638.585	863.9605	4091.195	1661.26
	12428	Ccna2	cyclin A2	626.8545	596.5005	445.792	918.7465
	12826	Col4a1	collagen, type IV, alpha 1	1018.985	2169.7	335.0875	1301.17
	13077	Cyp1a2	cytochrome P450, family 1, subfamily a, polypeptide 2	5712.18	2987.1	8898.78	4259.01
	13110	Cyp2j6	cytochrome P450, family 2, subfamily j, polypeptide 6	253.846	213.2725	371.4845	198.592
	13190	Dct	dopachrome tautomerase	224.8835	212.6675	516.774	158.9425
	13605	Ect2	ect2 oncogene	208.8055	246.349	204.839	466.2525
	13653	Egr1	early growth response 1	1272.474	693.725	197.582	1060.817
	13897	Es22	esterase 22	1781.53	823.8435	2322.545	272.777

Location	Entrez ID	Symbol	Gene Name	rAd.β-gal before	rAd.β-gal after	rAd.A20 before	rAd.A20 after
	14026	Evl	Ena-vasodilator stimulated phosphoprotein	99.11315	60.36285	107.7025	49.05105
	14107	Fat1	FAT tumor suppressor homolog 1 (Drosophila)	936.3075	1362.5	489.116	1012.699
	14126	Ms4a2	membrane-spanning 4-domains, subfamily A, member 2	238.4925	154.244	275.3885	163.4165
	14130	Fcgr2b	Fc receptor, IgG, low affinity IIb	556.296	454.0175	955.6935	534.5895
	14156	Fen1	flap structure specific endonuclease 1	393.9385	633.6615	268.622	564.818
	14219	Ctgf	connective tissue growth factor	314.6645	475.726	266.7695	1045.902
	142688	Asb13	ankyrin repeat and SOCS box-containing 13	137.441	118.845	219.9885	131.507
	14281	Fos	FBJ osteosarcoma oncogene	273.1725	432.9495	160.0665	394.106
	14431	Gamt	guanidinoacetate methyltransferase	1796.615	804.098	2443.325	1194.49
	14528	Gch1	GTP cyclohydrolase 1	640.447	335.5335	1077.699	476.918
	14605	Tsc22d3	TSC22 domain family, member 3	936.8285	845.275	1081.91	634.172
	14609	Gja1	gap junction protein, alpha 1	133.5783	276.7345	94.16725	204.231
	14612	Gja4	gap junction protein, alpha 4	209.665	262.5585	136.4535	252.082
	14685	Gnat1	guanine nucleotide binding protein, alpha transducing 1	625.261	745.6845	485.0885	842.0495
	14718	Got1	glutamate oxaloacetate transaminase 1, soluble	1753.408	3549.725	1272.31	5799.59
	14824	Grn	granulin	6489.93	7317.77	3326.51	7096.44
	14858	Gsta2	glutathione S-transferase, alpha 2 (Yc2)	1639.96	1029.905	1813.42	503.8585
	14867	Gstm6	glutathione S-transferase, mu 6	867.1345	591.617	1422.835	557.8685
	15368	Hmox1	heme oxygenase (decycling) 1	571.237	1145.543	427.4905	1021.015
	15450	Lipc	lipase, hepatic	1269.985	848.181	2001.11	670.2885
	15483	Hsd11b1	hydroxysteroid 11-beta dehydrogenase 1	4875.91	4135.79	7965.115	4503.32
	16006	Igfbp1	insulin-like growth factor binding protein 1	3015.465	5583.105	547.4005	4057.545
	16007	Cyr61	cysteine rich protein 61	266.377	298.903	236.6455	427.775
	16009	Igfbp3	insulin-like growth factor binding protein 3	107.9165	158.148	171.909	77.65705
	16319	Incenp	inner centromere protein	309.4985	294.2955	142.4805	308.7345
	16426	Itih3	inter-alpha trypsin inhibitor, heavy chain 3	8045.745	8237.555	5531.815	10470.05
	16564	Kif21a	kinesin family member 21A	742.279	989.904	752.6225	1292.05
	16636	Klra5	killer cell lectin-like receptor, subfamily A, member 5	199.1765	159.077	268.2345	139.6065
	16653	Kras	v-Ki-ras2 Kirsten rat sarcoma viral oncogene homolog	911.767	1328.835	531.5645	908.84
	16668	Krt18	keratin 18	4960.115	8346.815	2558.13	6268.11
	16691	Krt8	keratin 8	4345.555	7904.315	1867.195	6006.4
	16854	Lgals3	lectin, galactose binding, soluble 3	1000.623	1288.655	494.4055	1042.129
	16881	Lig1	ligase I, DNA, ATP-dependent	1113.415	1319.17	416.172	771.7125
	170791	Rbm39	RNA binding motif protein 39	220.272	306.032	196.4385	359.14
	17131	Smad7	MAD homolog 7 (Drosophila)	151.005	168.257	165.603	87.8513
	17132	Maf	avian musculoaponeurotic fibrosarcoma (v-maf) AS42 oncogene homolog	361.127	380.472	706.361	376.7245
	17164	Mapkapk2	MAP kinase-activated protein kinase 2	1017.18	1770.735	644.6305	1232.115
	17279	Melk	maternal embryonic leucine zipper kinase	86.35075	110.4875	74.63225	171.401
	17345	Mki67	antigen identified by monoclonal antibody Ki 67	521.9785	422.958	291.7635	795.431
	17380	Mme	membrane metallo endopeptidase	99.6709	98.01485	182.4215	84.98365
	17748	Mt1	metallothionein 1	315.427	738.431	152.242	487.8485
	17873	Gadd45b	growth arrest and DNA-damage-inducible 45 beta	554.7475	342.6325	158.0828	552.147
	17937	Nab2	Ngfi-A binding protein 2	291.198	231.109	181.502	325.4595
	17988	Ndrg1	N-myc downstream regulated gene 1	202.3885	346.158	123.762	448.1
	18489	Reg3b	regenerating islet-derived 3 beta	57.3579	250.9458	64.99165	406.39
	18770	Pklr	pyruvate kinase liver and red blood cell	705.598	418.0555	1064.225	500.2425
	18810	Plec1	plectin 1	1360.765	1736.805	725.3945	1424.455
	192176	Flna	filamin, alpha	586.8505	819.691	341.9105	598.5535
	19713	Ret	ret proto-oncogene	60.5572	89.31145	62.51685	116.385
	20194	S100a10	S100 calcium binding protein A10 (calpactin)	3382.97	5736.265	2276.36	4732.375
	20229	Sat1	spermidine/spermine N1-acetyl transferase 1	1267.48	2297.28	1165.29	2994.055
	20515	Slc20a1	solute carrier family 20, member 1	97.67985	101.5785	77.3173	162.996
	20525	Slc2a1	solute carrier family 2 (facilitated glucose transporter), member 1	418.481	580.8245	232.631	703.2055
	20617	Snca	synuclein, alpha	134.0425	85.4202	155.159	68.31175
	20848	Stat3	signal transducer and activator of transcription 3	1737.83	1883.225	1042.018	2186.215
	20969	Sdc1	syndecan 1	4110.37	5038.695	2850.825	5600.085
	210808	9030625A04Rik	RIKEN cDNA 9030625A04 gene	467.7245	607.7045	291.6895	606.186
	212442	Lactb2	lactamase, beta 2	1321.41	916.8095	2607.845	1288.395
	212933	Pm20d1	peptidase M20 domain containing 1	994.633	539.2595	1196.725	717.379
	212980	Slc45a3	solute carrier family 45, member 3	349.6225	390.1175	213.171	420.614
	21346	Tagln2	transgelin 2	602.8345	853.212	238.4745	505.8865
	214403	EG214403	predicted gene, EG214403	413.8755	381.443	819.6805	464.9745
	215693	Zmat1	zinc finger, matrin type 1	136.3755	118.016	149.023	81.41245
	217316	Slc16a5	solute carrier family 16 (monocarboxylic acid transporters), member 5	306.3215	279.96	190.671	625.4635
	21817	Tgm2	transglutaminase 2, C polypeptide	2708.715	4713.395	998.3165	3078.41
	21826	Thbs2	thrombospondin 2	201.3555	254.691	164.762	304.324
	21848	Trim24	tripartite motif-containing 24	407.395	705.8615	240.441	490.912
	21873	Tjp2	tight junction protein 2	276.527	368.6215	127.1595	239.622
	21973	Top2a	topoisomerase (DNA) II alpha	726.1255	870.4545	422.2025	1158.455
	22116	Tsks	testis-specific serine kinase substrate	71.0617	208.5245	93.9361	272.6435
	22154	Tubb5	tubulin, beta 5	2855.98	4786.035	1641.895	3525.01
	223650	Eppk1	epiplakin 1	813.6625	1197.719	335.656	1100.615
	224826	Ubr2	ubiquitin protein ligase E3 component n-recognin 2	409.585	386.4675	472.8675	271.583
	226551	AI848100	expressed sequence AI848100	298.6375	669.313	350.2785	590.1195
	22661	Zfp148	zinc finger protein 148	247.734	165.427	450.126	172.9945
	226823	Kctd3	potassium channel tetramerisation domain containing 3	624.461	1022.009	339.6545	613.78
	228026	Pdk1	pyruvate dehydrogenase kinase, isoenzyme 1	142.4265	75.487	211.8845	79.3951
	228482	Arhgap11a	Rho GTPase activating protein 11A	237.034	291.5525	186.188	389.7745
	228812	Pigu	phosphatidylinositol glycan anchor biosynthesis, class U	1425.28	908.8605	1468.845	865.4505
	229302	Tm4sf4	transmembrane 4 superfamily member 4	2437.05	3193.425	2120.175	3761.13

Location	Entrez ID	Symbol	Gene Name	rAd.β-gal before	rAd.β-gal after	rAd.A20 before	rAd.A20 after
	229589	Prune	prune homolog (Drosophila)	810.1745	1031.356	482.722	909.509
	229841	Cenpe	centromere protein E	19.07285	27.66715	17.6269	40.8008
	230101	Gba2	glucosidase beta 2	126.2015	124.6985	195.2555	104.608
	231070	Insig1	insulin induced gene 1	2756.145	2459.55	2700.02	1570.26
	232232	Hdac11	histone deacetylase 11	365.0145	236.3595	549.7165	246.839
	235293	Sc5d	sterol-C5-desaturase (fungal ERG3, delta-5-desaturase) homolog (S. cerevisae)	1384.97	831.766	1935.27	987.8845
	235493	BC031353	cDNA sequence BC031353	450.0065	383.523	781.829	426.357
	235611	Plxnb1	plexin B1	341.3765	355.0535	120.4275	315.434
	235674	Acaa1b	acetyl-Coenzyme A acyltransferase 1B	9314.005	5326.4	9230.165	4356.16
	236904	Klhl15	kelch-like 15 (Drosophila)	110.4455	182.3645	85.24885	154.251
	237436	Gas2l3	growth arrest-specific 2 like 3	120.8208	138.726	130.6307	303.2935
	23849	Klf6	Kruppel-like factor 6	249.0805	335.361	75.1646	238.713
	23872	Ets2	E26 avian leukemia oncogene 2, 3' domain	987.964	1522.51	354.0635	1083.69
	23992	Prkra	protein kinase, interferon inducible double stranded RNA dependent activator	384.1465	348.51	480.725	238.8895
	241447	Lass6	LAG1 homolog, ceramide synthase 6	340.0055	479.6225	154.017	379.3945
	259300	Ehd2	EH-domain containing 2	132.7378	63.1651	122.169	26.91845
	26384	Gnpda1	glucosamine-6-phosphate deaminase 1	748.0025	673.8005	703.2295	382.7625
	26459	Slc27a5	solute carrier family 27 (fatty acid transporter), member 5	2929.96	1985.86	4728.455	2608.96
	268396	Sh3pxd2b	SH3 and PX domains 2B	573.918	521.0415	954.494	378.1575
	268822	Adck5	aarF domain containing kinase 5	323.767	269.477	448.8605	248.092
	26934	Racgap1	Rac GTPase-activating protein 1	139.479	144.586	105.8594	215.469
	269423	3110057O12Rik	RIKEN cDNA 3110057O12 gene	96.7893	79.4182	191.1585	82.7576
	27226	Pla2g7	phospholipase A2, group VII (platelet-activating factor acetylhydrolase, plasma)	162.7925	473.248	119.053	235.0185
	27377	Yme1l1	YME1-like 1 (S. cerevisiae)	98.37365	81.35005	167.2945	86.63515
	27981	D4Wsu53e	DNA segment, Chr 4, Wayne State University 53, expressed	1223.105	1140.395	1586.75	862.208
	29876	Clic4	chloride intracellular channel 4 (mitochondrial)	599.5875	838.842	602.713	1017.234
	319760	D130020L05Rik	RIKEN cDNA D130020L05 gene	584.284	360.0325	812.193	409.621
	399578	8030447M02Rik	RIKEN cDNA 8030447M02 gene	233.8435	181.3785	348.368	154.216
	433771	2310028O11Rik	RIKEN cDNA 2310028O11 gene	156.1625	544.8595	140.603	585.7435
	50702	Cfhr1	complement factor H-related 1	1396.354	891.4115	3108.99	1701.44
	50770	Atp11a	ATPase, class VI, type 11A	281.029	371.629	144.4215	358.7625
	50784	Ppap2c	phosphatidic acid phosphatase type 2C	728.125	1026.796	539.6625	1022.797
	50877	Neu3	neuraminidase 3	139.6245	163.654	77.57105	136.3455
	50918	Myadm	myeloid-associated differentiation marker	771.327	1047.621	565.861	1161.22
	52276	Cdca8	cell division cycle associated 8	652.866	566.6575	360.356	781.302
	52428	Rhpn2	rhophilin, Rho GTPase binding protein 2	580.576	978.376	394.938	1058.165
	52696	Zwint	ZW10 interactor	272.284	404.847	144.5745	258.107
	53322	Nucb2	nucleobindin 2	133.8275	193.6415	75.5551	157.7555
	54140	Avpr1a	arginine vasopressin receptor 1A	481.701	578.291	230.566	592.5025
	54392	Ncapg	non-SMC condensin I complex, subunit G	152.366	165.934	120.338	288.6565
	54648	Ccdc120	coiled-coil domain containing 120	103.2988	190.928	74.9818	192.8475
	55950	Bri3	brain protein I3	1701.935	1594.51	2523.415	1329.99
	56384	Letm1	leucine zipper-EF-hand containing transmembrane protein 1	218.908	183.8755	271.6585	151.959
	56708	Clcf1	cardiotrophin-like cytokine factor 1	30.82725	55.96085	14.94415	45.94485
	56722	Litaf	LPS-induced TN factor	1653.14	2332.83	1047.52	2258.56
	58170	Accn5	amiloride-sensitive cation channel 5, intestinal	108.3241	156.037	227.3665	84.44755
	59036	Dact1	dapper homolog 1, antagonist of beta-catenin (xenopus)	224.394	282.827	208.521	440.965
	622404	RP23-195K8.6	hypothetical protein LOC622404	1051.16	783.7875	1123.677	576.2495
	639390	BC094435	cDNA sequence BC094435	5769.11	8156.285	2973.34	5842.17
	64384	Sirt3	sirtuin 3 (silent mating type information regulation 2, homolog) 3 (S. cerevisiae)	1455.96	925.024	2038	1191.485
	65945	Clstn1	calsyntenin 1	182.7247	113.5136	586.7795	113.4536
	66082	Abhd6	abhydrolase domain containing 6	200.598	274.5585	345.709	183.5215
	66302	Fam82b	family with sequence similarity 82, member B	401.1025	604.07	319.8435	749.25
	66361	Zfand1	zinc finger, AN1-type domain 1	158.318	172.211	232.2335	131.3185
	66431	1810049H13Rik	RIKEN cDNA 1810049H13 gene	357.8805	308.5455	516.988	286.2075
	66438	Hamp2	hepcidin antimicrobial peptide 2	3648.645	3313.61	3806.52	2035.76
	66521	Rwdd1	RWD domain containing 1	50.70065	57.4556	33.94945	70.9022
	66834	Acot13	acyl-CoA thioesterase 13	1427.355	942.489	1963.195	918.484
	66898	Baiap2l1	BAI1-associated protein 2-like 1	515.1035	1077.058	637.218	1459.205
	67015	Ccdc91	coiled-coil domain containing 91	416.518	303.335	708.407	331.2565
	67217	2810055F11Rik	RIKEN cDNA 2810055F11 gene	250.074	325.9985	559.574	234.269
	67399	Pdlim7	PDZ and LIM domain 7	204.119	350.931	118.0005	232.4845
	67455	Klhl13	kelch-like 13 (Drosophila)	818.0325	442.7045	1703.715	533.6045
	67470	Abcg8	ATP-binding cassette, sub-family G (WHITE), member 8	748.558	343.447	931.0225	521.413
	67523	1700094J05Rik	RIKEN cDNA 1700094J05 gene	486.5705	1361.874	602.9205	1717.31
	68041	Mid1ip1	Mid1 interacting protein 1 (gastrulation specific G12-like (zebrafish))	1224.396	766.581	1304.345	383.124
	68054	Serpina12	serine (or cysteine) peptidase inhibitor, clade A (alpha-1 antiproteinase, antitrypsin), member 12	1182.641	757.5515	1459.74	300.639
	68180	Hyi	hydroxypyruvate isomerase homolog (E. coli)	992.586	549.7405	1086.3	536.8325
	68298	Ncapd2	non-SMC condensin I complex, subunit D2	371.6535	349.8945	235.787	454.5635
	68347	0610011F06Rik	RIKEN cDNA 0610011F06 gene	2032.005	1642.51	2978.96	1571.38
	68371	Pbld	phenazine biosynthesis-like protein domain containing	750.524	315.6695	1415.165	646.6555
	68526	Gpr155	G protein-coupled receptor 155	413.68	345.404	615.7005	344.143
	68552	1110003E01Rik	RIKEN cDNA 1110003E01 gene	815.3235	554.218	1152.47	406.537
	68576	Hbxip	hepatitis B virus x interacting protein	896.5665	827.1955	1091.394	511.6495
	68612	Ube2c	ubiquitin-conjugating enzyme E2C	607.005	852.9605	706.904	1429.085
	68632	Myct1	myc target 1	47.98645	41.1247	74.8641	38.53385

Location	Entrez ID	Symbol	Gene Name	rAd.β-gal before	rAd.β-gal after	rAd.A20 before	rAd.A20 after
	68636	Fahd1	fumarylacetoacetate hydrolase domain containing 1	429.926	333.5315	727.8095	374.688
	68743	Anln	anillin, actin binding protein	112.2969	158.075	127.51	244.1735
	68841	1110054M08Rik	RIKEN cDNA 1110054M08 gene	355.1795	196.801	420.949	240.2915
	68955	1500001A10Rik	RIKEN cDNA 1500001A10 gene	39.02065	44.31075	30.42605	76.83925
	69772	Bdh2	3-hydroxybutyrate dehydrogenase, type 2	382.959	290.821	590.9635	293.523
	69786	Tprkb	Tp53rk binding protein	929.246	821.2045	1913.125	996.237
	70031	Cmtm8	CKLF-like MARVEL transmembrane domain containing 8	1219.675	747.461	1237.225	652.1525
	70061	Sdr9c7	4short chain dehydrogenase/reductase family 9C, member 7	194.7664	143.497	441.945	213.6885
	70229	2410024N18Rik	RIKEN cDNA 2410024N18 gene	712.5535	516.4155	967.845	484.197
	70564	5730469M10Rik	RIKEN cDNA 5730469M10 gene	1001.26	746.975	1241.415	649.761
	70799	Cep192	centrosomal protein 192	112.4145	145.6375	89.929	158.7385
	71268	Lrrfip2	leucine rich repeat (in FLII) interacting protein 2	795.4875	940.9	541.474	895.5995
	71519	Cyp2u1	cytochrome P450, family 2, subfamily u, polypeptide 1	320.991	193.7285	481.079	224.237
	71562	Afmid	arylformamidase	848.22	434.135	1350.395	557.0615
	71670	Acy3	aspartoacylase (aminoacylase) 3	1026.554	632.237	1498.085	813.724
	71687	Tmem25	transmembrane protein 25	266.384	103.7726	250.4865	144.338
	71761	Amdhd1	amidohydrolase domain containing 1	119.4681	53.53135	177.4635	92.1539
	71779	8-Mar	membrane-associated ring finger (C3HC4) 8	616.037	598.0395	1065.805	636.172
	71891	Cdadc1	cytidine and dCMP deaminase domain containing 1	353.2145	280.5685	632.809	310.763
	72852	Mblac2	metallo-beta-lactamase domain containing 2	67.299	63.7138	100.2863	53.56465
	72999	Insig2	insulin induced gene 2	2769.735	4294.4	2005.51	6113.49
	73176	3110040M04Rik	RIKEN cDNA 3110040M04 gene	86.6517	118.1415	77.91605	147.532
	73449	1700066B19Rik	RIKEN cDNA 1700066B19 gene	389.1345	218.5795	485.108	246.2965
	73673	2410076I21Rik	RIKEN cDNA 2410076I21 gene	96.2235	127.603	66.7048	119.8355
	74041	4632434I11Rik	RIKEN cDNA 4632434I11 gene	241.175	372.8795	122.35	248.73
	74043	Pex26	peroxisomal biogenesis factor 26	329.5405	294.871	560.7905	321.3825
	74122	Tmem43	transmembrane protein 43	319.55	537.95	222.015	403.885
	74159	Acbd5	acyl-Coenzyme A binding domain containing 5	1328.155	885.962	2312.285	1199.385
	74411	Ppapdc2	phosphatidic acid phosphatase type 2 domain containing 2	147.8129	113.4373	130.5535	79.9296
	76187	Adhfe1	alcohol dehydrogenase, iron containing, 1	990.038	817.133	1668.835	944.1495
	76682	1500002K03Rik	RIKEN cDNA 1500002K03 gene	210.345	168.647	327.356	98.3955
	76857	Spopl	speckle-type POZ protein-like	62.79655	71.3262	80.50335	45.2106
	76939	2510042H12Rik	RIKEN cDNA 2510042H12 gene	518.582	240.057	506.1305	165.402
	77090	Ocel1	occludin/ELL domain containing 1	407.352	428.992	595.543	352.6655
	77219	Ptgr2	prostaglandin reductase 2	198.673	203.9845	376.884	187.993
	81016	V1rd2	vomeronasal 1 receptor, D2	134.123	122.3075	79.6699	142.9245
	81910	Rrbp1	ribosome binding protein 1	1981.03	2183.12	1679.89	2931.995
	83814	Nedd4l	neural precursor cell expressed, developmentally down-regulated gene 4-like	727.355	1246.215	434.818	956.867
	85308	Fam158a	family with sequence similarity 158, member A	769.7375	337.2955	770.656	369.7795
	97165	Hmgb2	high mobility group box 2	537.7035	788.871	450.497	920.0005
	97983	C85319	expressed sequence C85319	145.1235	105.7569	221.245	118.345
	98415	Nucks1	nuclear casein kinase and cyclin-dependent kinase substrate 1	442.256	499.3775	298.1865	601.9935

**q12**	100040972	Tceal7	transcription elongation factor A (SII)-like 7	54.61105	43.2536	28.2668	41.07295
	100042616	Gm3932	predicted gene 3932	611.119	431.1935	264.647	326.5105
	100049166	LOC100049166	hypothetical protein LOC100049166	1418.71	949.7725	599.729	934.422
	100689	Spon2	spondin 2, extracellular matrix protein	382.9935	323.099	168.46	236.5645
	101187	Parp11	poly (ADP-ribose) polymerase family, member 11	705.948	462.0045	255.4295	466.4325
	102589	AI835735	expressed sequence AI835735	92.15065	96.0808	334.751	150.7715
	104346	Gas8	growth arrest specific 8	291.4205	308.4305	586.588	392.2965
	105095	AA517650	expressed sequence AA517650	312.196	204.344	152.1745	160.5785
	107823	Whsc1	Wolf-Hirschhorn syndrome candidate 1 (human)	355.9425	407.75	194.864	289.508
	108767	Pnrc1	proline-rich nuclear receptor coactivator 1	2209.875	1602.165	904.379	1499.94
	109095	Rbm15b	RNA binding motif protein 15B	724.9085	541.659	359.753	474.0965
	110382	C8b	complement component 8, beta polypeptide	1009.55	971.808	2921.745	1477.815
	110595	Timp4	tissue inhibitor of metalloproteinase 4	66.25325	57.2733	26.75005	48.4236
	114714	Rad51c	RAD51 homolog c (S. cerevisiae)	858.4335	913.1535	495.9725	669.6265
	11643	Akap4	A kinase (PRKA) anchor protein 4	405.552	298.1295	140.6575	206.717
	11650	Alppl2	alkaline phosphatase, placental-like 2	746.8075	442.001	310.6845	390.7015
	11658	Alcam	activated leukocyte cell adhesion molecule	408.3735	261.557	132.962	176.528
	11684	Alox12	arachidonate 12-lipoxygenase	238.0015	198.597	135.1795	171.5675
	11772	Ap2a2	adaptor protein complex AP-2, alpha 2 subunit	317.338	214.954	156.1115	261.6865
	11798	Xiap	X-linked inhibitor of apoptosis	24.9576	37.30985	50.72705	59.26675
	11865	Arntl	aryl hydrocarbon receptor nuclear translocator-like	341.162	215.023	153.1235	263.3265
	12032	Bcan	brevican	338.518	247.867	169.22	205.7895
	12051	Bcl3	B-cell leukemia/lymphoma 3	734.955	754.9065	289.34	527.407
	12259	C1qa	complement component 1, q subcomponent, alpha polypeptide	1221.705	1351.06	652.8225	1064.325
	12307	Calb1	calbindin 1	217.3765	168.513	92.8966	151.168
	12419	Cbx5	chromobox homolog 5 (Drosophila HP1a)	821.762	633.6	404.835	608.8285
	12449	Ccnf	cyclin F	646.6025	696.69	364.1905	571.9365
	12491	Cd36	CD36 antigen	1043.537	1021.966	574.0735	966.6495
	12505	Cd44	CD44 antigen	438.2455	614.226	185.362	305.371
	12577	Cdkn1c	cyclin-dependent kinase inhibitor 1C (P57)	620.8195	389.909	173.4875	148.998
	12659	Ovgp1	oviductal glycoprotein 1	231.551	165.6855	114.133	149.1135
	12660	Chka	choline kinase alpha	769.5205	235.0905	108.8752	251.1285
	12745	Clgn	calmegin	244.4985	186.8605	136.2195	231.375
	12763	Cmah	cytidine monophospho-N-acetylneuraminic acid hydroxylase	597.014	575.1655	1243.925	850.103
	12874	Cpd	carboxypeptidase D	184.355	138.923	87.95205	132.3665
	12950	Hapln1	hyaluronan and proteoglycan link protein 1	229.9845	133.927	105.2525	96.3119

Location	Entrez ID	Symbol	Gene Name	rAd.β-gal before	rAd.β-gal after	rAd.A20 before	rAd.A20 after
	12953	Cry2	cryptochrome 2 (photolyase-like)	1166.64	797.094	503.97	651.7605
	12978	Csf1r	colony stimulating factor 1 receptor	2589.125	1919.18	1362.25	2019.47
	13058	Cybb	cytochrome b-245, beta polypeptide	430.0595	229.1805	153.808	153.632
	13176	Dcc	deleted in colorectal carcinoma	202.698	114.4009	94.77125	112.0665
	13370	Dio1	deiodinase, iodothyronine, type I	316.249	626.2635	949.9075	485.909
	13446	Doc2a	double C2, alpha	547.748	352.485	234.755	272.937
	13508	Dscam	Down syndrome cell adhesion molecule	355.9055	242.107	183.4475	231.0235
	13829	Epb4.9	erythrocyte protein band 4.9	214.7475	167.1535	110.509	126.5105
	13929	Amz2	archaelysin family metallopeptidase 2	399.815	292.2205	198.118	294.1335
	14050	Eya3	eyes absent 3 homolog (Drosophila)	696.8235	738.3965	401.2185	537.594
	14057	Sfxn1	sideroflexin 1	2779.21	1909.455	1541.515	1084.28
	140918	Slc7a12	solute carrier family 7 (cationic amino acid transporter, y+ system), member 12	165.4755	102.5955	81.4368	120.283
	14148	Fdx1	ferredoxin 1	2260.75	2322.905	4610.22	2847.65
	14211	Smc2	structural maintenance of chromosomes 2	93.88	176.5605	222.778	179.4415
	14255	Flt3	FMS-like tyrosine kinase 3	784.5105	631.76	407.459	697.622
	14385	Slc37a4	solute carrier family 37 (glucose-6-phosphate transporter), member 4	1449.01	1722.77	2699.245	2258.21
	14457	Gas7	growth arrest specific 7	119.2265	92.0577	57.20155	87.12805
	14468	Gbp1	guanylate binding protein 1	2051.26	1033.812	409.3855	1231.628
	14469	Gbp2	guanylate binding protein 2	1190.105	719.811	243.6835	681.7115
	14573	Gdnf	glial cell line derived neurotrophic factor	339.0155	242.4045	148.5775	198.042
	14600	Ghr	growth hormone receptor	504.635	261.502	1518.76	776.5835
	14674	Gna13	guanine nucleotide binding protein, alpha 13	1737.21	1447.06	860.5685	1238.895
	14807	Grik3	glutamate receptor, ionotropic, kainate 3	427.2095	276.662	177.344	233.3405
	14825	Cxcl1	chemokine (C-X-C motif) ligand 1	4090.96	3109.215	962.6855	2482.125
	14960	H2-Aa	histocompatibility 2, class II antigen A, alpha	984.796	719.2355	327.7185	397.0325
	14961	H2-Ab1	histocompatibility 2, class II antigen A, beta 1	1541.925	1202.785	445.4765	803.9075
	14964	H2-D1	histocompatibility 2, D region locus 1	7697.35	7879.88	4565.605	7152.69
	14969	H2-Eb1	histocompatibility 2, class II antigen E beta	1389.05	1013.537	469.868	744.2075
	14972	H2-K1	histocompatibility 2, K1, K region	1513.885	917.023	592.019	969.7175
	14998	H2-DMa	histocompatibility 2, class II, locus DMa	386.9075	247.7815	139.766	227.709
	15000	H2-DMb2	histocompatibility 2, class II, locus Mb2	56.5541	45.3579	24.50895	37.0575
	15081	H3f3b	H3 histone, family 3B	417.4785	295.063	175.2085	232.058
	15229	Foxd1	forkhead box D1	48.2244	35.88775	21.62675	28.3654
	15257	Hipk1	homeodomain interacting protein kinase 1	536.331	388.0895	245.1535	357.157
	15260	Hira	histone cell cycle regulation defective homolog A (S. cerevisiae)	158.4405	114.818	78.93305	88.842
	15511	Hspa1b	heat shock protein 1B	331.7175	484.26	1312.06	497.728
	15562	Htr4	5 hydroxytryptamine (serotonin) receptor 4	313.3225	239.6405	132.7715	278.973
	15900	Irf8	interferon regulatory factor 8	732.8535	475.123	241.917	492.0805
	15939	Ier5	immediate early response 5	391.0125	354.233	169.2625	279.8305
	15945	Cxcl10	chemokine (C-X-C motif) ligand 10	2376.63	1094.475	363.0855	1882.845
	15951	Ifi204	interferon activated gene 204	74.9131	77.2753	31.2889	45.6797
	15953	Ifi47	interferon gamma inducible protein 47	4086.39	2104.875	1781.875	3111.265
	15978	Ifng	interferon gamma	83.18885	75.969	41.0942	65.3339
	15983	Ifrd2	interferon-related developmental regulator 2	809.774	1104.23	1467.815	1445.945
	16008	Igfbp2	insulin-like growth factor binding protein 2	730.652	545.5855	1837.095	1895.13
	16149	Cd74	CD74 antigen (invariant polypeptide of major histocompatibility complex, class II antigen-associated)	3531.255	2443.555	1051.818	1790.66
	16181	Il1rn	interleukin 1 receptor antagonist	264.8335	409.946	70.0083	191.2305
	16186	Il2rg	interleukin 2 receptor, gamma chain	259.744	164.3595	62.64405	121.6985
	16365	Irg1	immunoresponsive gene 1	355.3415	256.415	127.395	215.3145
	16408	Itgal	integrin alpha L	407.3435	283.258	206.086	297.4055
	16452	Jak2	Janus kinase 2	1207.055	848.103	516.1615	979.951
	16500	Kcnb1	potassium voltage gated channel, Shab-related subfamily, member 1	254.45	193.2825	139.9415	199.079
	16518	Kcnj2	potassium inwardly-rectifying channel, subfamily J, member 2	111.321	71.33725	54.00675	50.1058
	16660	Krt31	keratin 31	342.696	271.349	155.944	250.915
	16782	Lamc2	laminin, gamma 2	429.721	326.395	201.9905	261.7465
	16796	Lasp1	LIM and SH3 protein 1	1974.905	1271.955	665.817	966.3355
	16852	Lgals1	lectin, galactose binding, soluble 1	3070.96	2079.485	1193.93	1308.43
	16875	Lhx8	LIM homeobox protein 8	152.7945	119.959	89.44905	95.86875
	16913	Psmb8	proteasome (prosome, macropain) subunit, beta type 8 (large multifunctional peptidase 7)	3807.965	1954.685	1315.1	1946.654
	16956	Lpl	lipoprotein lipase	1200.257	652.7345	476.2795	672.423
	170459	Stard4	StAR-related lipid transfer (START) domain containing 4	428.99	750.5615	1318.76	764.4675
	170460	Stard5	StAR-related lipid transfer (START) domain containing 5	558.1555	675.37	1383.89	1139.188
	17069	Ly6e	lymphocyte antigen 6 complex, locus E	634.8395	478.5405	267.617	382.891
	17105	Lyz2	lysozyme 2	1532.81	840.0255	646.7635	724.3865
	17119	Mxd1	MAX dimerization protein 1	452.635	373.221	258.372	352.5725
	17184	Matr3	matrin 3	256.148	186.591	118.9795	135.026
	17218	Mcm5	minichromosome maintenance deficient 5, cell division cycle 46 (S. cerevisiae)	1202.63	1547.735	559.9345	1413.725
	17221	Cd46	CD46 antigen, complement regulatory protein	768.5115	509.1475	420.5435	462.3615
	17329	Cxcl9	chemokine (C-X-C motif) ligand 9	3152.665	1755.115	538.357	2166.464
	17476	Mpeg1	macrophage expressed gene 1	3282.155	1657.82	1560.365	2014.04
	17684	Cited2	Cbp/p300-interacting transactivator, with Glu/Asp-rich carboxy-terminal domain, 2	1160.691	572.562	460.961	444.7485
	17688	Msh6	mutS homolog 6 (E. coli)	287.886	373.3195	148.832	196.0235
	17776	Mast2	microtubule associated serine/threonine kinase 2	1001.469	1116.5	482.5065	679.3765
	17843	Mup4	major urinary protein 4	18.32165	26.51025	49.8019	26.0294
	17984	Ndn	necdin	82.5188	75.8153	283.4195	133.116
	18125	Nos1	nitric oxide synthase 1, neuronal	795.1345	543.732	283.652	455.616

Location	Entrez ID	Symbol	Gene Name	rAd.β-gal before	rAd.β-gal after	rAd.A20 before	rAd.A20 after
	18128	Notch1	Notch gene homolog 1 (Drosophila)	788.191	492.023	321.0345	290.6925
	18143	Npas2	neuronal PAS domain protein 2	209.096	76.9553	72.63435	78.46295
	18173	Slc11a1	solute carrier family 11 (proton-coupled divalent metal ion transporters), member 1	435.549	474.108	193.941	350.162
	18186	Nrp1	neuropilin 1	464.9265	587.8875	1193.44	843.01
	18548	Pcsk1	proprotein convertase subtilisin/kexin type 1	269.4225	210.734	130.129	196.1425
	18583	Pde7a	phosphodiesterase 7A	502.0775	387.468	279.1625	338.502
	18627	Per2	period homolog 2 (Drosophila)	71.09275	155.669	218.1535	131.8635
	18796	Plcb2	phospholipase C, beta 2	582.547	433.549	292.64	398.9135
	18830	Pltp	phospholipid transfer protein	376.245	325.2995	197.1695	219.726
	19015	Ppard	peroxisome proliferator activator receptor delta	348.029	257.1625	169.117	208.3385
	19142	Prss12	protease, serine, 12 neurotrypsin (motopsin)	461.6745	312.8865	225.9645	279.2335
	19205	Ptbp1	polypyrimidine tract binding protein 1	2825.94	2330.105	7772.4	4833.66
	192136	5033411D12Rik	RIKEN cDNA 5033411D12 gene	223.6895	209.2175	535.4195	261.6645
	19264	Ptprc	protein tyrosine phosphatase, receptor type, C	394.585	340.648	186.7775	242.39
	192656	Ripk2	receptor (TNFRSF)-interacting serine-threonine kinase 2	530.609	832.758	269.132	390.592
	19373	Rag1	recombination activating gene 1	188.975	273.214	500.984	279.3265
	194126	Mtmr11	myotubularin related protein 11	202.7455	208.053	96.5735	138.2305
	19645	Rb1	retinoblastoma 1	664.0205	533.004	313.422	552.7005
	19646	Rbbp4	retinoblastoma binding protein 4	628.899	1080.308	1405.03	1034.79
	19664	Rbpj	recombination signal binding protein for immunoglobulin kappa J region	904.5095	667.0415	458.3345	615.774
	19725	Rfx2	regulatory factor X, 2 (influences HLA class II expression)	266.7095	188.553	110.6325	147.288
	19777	C80913	expressed sequence C80913	1455.185	1062.794	805.439	1101.57
	19892	Rpe65	retinal pigment epithelium 65	294.825	232.715	133.8665	279.0575
	20304	Ccl5	chemokine (C-C motif) ligand 5	780.48	576.7615	346.46	544.0985
	20310	Cxcl2	chemokine (C-X-C motif) ligand 2	211.05	247.6835	33.6852	67.3737
	20540	Slc7a7	solute carrier family 7 (cationic amino acid transporter, y+ system), member 7	487.112	472.924	259.5025	289.827
	20588	Smarcc1	SWI/SNF related, matrix associated, actin dependent regulator of chromatin, subfamily c, member 1	839.464	691.9845	392.698	547.45
	20682	Sox9	SRY-box containing gene 9	151.144	142.1775	57.54735	91.1206
	20715	Serpina3g	serine (or cysteine) peptidase inhibitor, clade A, member 3G	2023.715	1237.39	495.542	1277.763
	20744	Strbp	spermatid perinuclear RNA binding protein	172.8995	139.874	68.78265	93.8402
	208228	Mobkl2a	MOB1, Mps One Binder kinase activator-like 2A (yeast)	301.1375	303.0695	164.475	239.554
	20893	Bhlhe40	basic helix-loop-helix family, member e40	784.497	1205.765	1289.815	1016.177
	209086	Samd9l	sterile alpha motif domain containing 9-like	607.8555	491.189	253.017	370.6355
	209200	Dtx3l	deltex 3-like (Drosophila)	2380.735	1662.425	1222.93	2129.505
	210105	Zfp719	zinc finger protein 719	195.9565	155.2215	98.2048	137.484
	211064	Alkbh1	alkB, alkylation repair homolog 1 (E. coli)	931.484	646.862	451.5605	557.729
	211945	Plekhh1	pleckstrin homology domain containing, family H (with MyTH4 domain) member 1	87.21055	93.23555	275.981	160.595
	212531	Sh3bgrl2	SH3 domain binding glutamic acid-rich protein like 2	247.281	239.518	460.247	275.0735
	213006	Mfsd4	major facilitator superfamily domain containing 4	345.5275	239.8475	138.9105	165.8705
	21355	Tap2	transporter 2, ATP-binding cassette, sub-family B (MDR/TAP)	1226.918	623.5565	431.1315	707.497
	21406	Tcf12	transcription factor 12	71.4957	61.473	39.5973	51.48375
	21413	Tcf4	transcription factor 4	253.568	206.853	125.628	149.3535
	21416	Tcf7l2	transcription factor 7-like 2, T-cell specific, HMG-box	541.788	371.0345	242.7685	327.972
	21417	Zeb1	zinc finger E-box binding homeobox 1	1494.335	1028.818	623.493	961.077
	214742	Rcor3	REST corepressor 3	590.1225	390.3865	255.2645	327.2575
	215789	Phactr2	phosphatase and actin regulator 2	43.28215	63.5759	76.63045	59.75055
	215900	Fam26f	family with sequence similarity 26, member F	481.633	213.5025	182.5765	298.8695
	216161	Sbno2	strawberry notch homolog 2 (Drosophila)	195.01	180.0415	101.746	163.778
	216551	1110067D22Rik	RIKEN cDNA 1110067D22 gene	430.215	326.8945	163.4725	238.57
	216613	Ccdc85a	coiled-coil domain containing 85A	106.1085	102.947	396.866	204.593
	216742	Fnip1	folliculin interacting protein 1	121.7496	213.588	313.102	312.87
	21685	Tef	thyrotroph embryonic factor	703.1285	912.542	1662.3	1110.265
	216874	Camta2	calmodulin binding transcription activator 2	1170.09	914.9605	665.5445	925.8335
	217578	Baz1a	bromodomain adjacent to zinc finger domain 1A	1068.342	824.7545	397.451	661.5845
	21812	Tgfbr1	transforming growth factor, beta receptor I	58.13285	81.14715	132.728	121.6615
	218203	Mylip	myosin regulatory light chain interacting protein	209.2185	238.38	377.2405	364.977
	21898	Tlr4	toll-like receptor 4	181.9075	121.72	84.97065	99.97835
	21929	Tnfaip3	tumor necrosis factor, alpha-induced protein 3	750.524	448.6005	166.1015	336.9945
	21958	Tnp1	transition protein 1	219.4485	190.7155	103.2368	133.857
	22025	Nr2c1	nuclear receptor subfamily 2, group C, member 1	217.8135	182.4915	110.5885	145.815
	22031	Traf3	TNF receptor-associated factor 3	372.1125	405.869	205.9985	281.186
	22113	Phlda2	pleckstrin homology-like domain, family A, member 2	298.557	235.457	142.472	183.0405
	22158	Tulp3	tubby-like protein 3	350.258	263.762	211.5995	261.817
	223332	Ranbp3l	RAN binding protein 3-like	188.577	145.84	115.226	162.175
	223775	Pim3	proviral integration site 3	744.415	1283.98	1680.735	1695.575
	223881	Rnd1	Rho family GTPase 1	317.519	516.5025	127.525	207.6125
	224617	Tbc1d24	TBC1 domain family, member 24	261.399	211.692	108.965	112.7302
	225896	Ubxn1	UBX domain protein 1	3286.115	2735.78	1616.245	1855.36
	226122	Ubtd1	ubiquitin domain containing 1	1299.455	844.394	511.2835	714.8015
	226412	R3hdm1	R3H domain 1 (binds single-stranded nucleic acids)	261.8735	190.832	116.017	140.5005
	226652	Arhgap30	Rho GTPase activating protein 30	643.5885	466.9665	276.8325	402.2155
	226747	Ahctf1	AT hook containing transcription factor 1	155.3165	114.9575	85.84015	122.089
	227195	Ino80d	INO80 complex subunit D	387.539	286.5245	209.138	318.239
	227331	Gigyf2	GRB10 interacting GYF protein 2	1051.999	598.71	418.725	533.1135
	22778	Ikzf1	IKAROS family zinc finger 1	601.1885	410.281	272.6855	408.3835
	228730	Gm114	gene model 114	881.558	656.423	407.929	500.462

Location	Entrez ID	Symbol	Gene Name	rAd.β-gal before	rAd.β-gal after	rAd.A20 before	rAd.A20 after
	229003	BC006779	cDNA sequence BC006779	1137.997	930.3665	498.3615	688.2265
	229900	Gbp6	guanylate binding protein 6	2010.51	1095.831	808.855	1470.401
	230649	Atpaf1	ATP synthase mitochondrial F1 complex assembly factor 1	191.664	238.1185	473.898	292.189
	230752	Fam176b	family with sequence similarity 176, member B	462.5815	311.9285	236.0165	296.131
	231093	Agbl5	ATP/GTP binding protein-like 5	717.961	417.2565	347.719	399.331
	231125	Zfyve28	zinc finger, FYVE domain containing 28	456.116	351.7405	199.8225	327.5055
	231655	Oasl1	2'-5' oligoadenylate synthetase-like 1	780.395	529.059	234.334	374.815
	231712	Trafd1	TRAF type zinc finger domain containing 1	1340	829.29	490.836	639.623
	232493	Gys2	glycogen synthase 2	776.2945	742.828	1535.3	973.0575
	232878	Zscan22	zinc finger and SCAN domain containing 22	531.1435	371.0445	243.926	358.037
	233099	Abpb	androgen binding protein beta	128.53	101.3813	63.0786	98.4027
	233649	Cnga4	cyclic nucleotide gated channel alpha 4	95.53285	73.67975	216.658	123.9045
	233833	Tnrc6a	trinucleotide repeat containing 6a	597.892	403.0355	319.627	374.7615
	234678	D230025D16Rik	RIKEN cDNA D230025D16 gene	341.0975	549.4685	771.9485	628.881
	235527	Plscr4	phospholipid scramblase 4	51.93245	50.6693	127.8725	72.9003
	237926	Rsad1	radical S-adenosyl methionine domain containing 1	358.3195	247.74	193.03	209.231
	23821	Bace1	beta-site APP cleaving enzyme 1	539.6165	364.3095	292.522	402.122
	23886	Gdf15	growth differentiation factor 15	1665.77	1243.135	677.951	982.9975
	239510	Phf20l1	PHD finger protein 20-like 1	246.7775	174.9805	141.269	244.5015
	23962	Oasl2	2'-5' oligoadenylate synthetase-like 2	332.209	191.6635	111.1357	182.016
	24088	Tlr2	toll-like receptor 2	604.829	395.0045	136.291	268.7345
	240880	Scyl3	SCY1-like 3 (S. cerevisiae)	217.294	267.311	458.622	353.793
	24108	Ubd	ubiquitin D	826.835	475.2125	210.5495	758.8665
	244233	Cd163l1	CD163 molecule-like 1	130.113	129.826	280.409	190.805
	246256	Fcgr4	Fc receptor, IgG, low affinity IV	672.7125	679.1735	336.0235	440.4055
	26388	Ifi202b	interferon activated gene 202B	235.2345	264.8255	118.0005	134.4141
	269424	Phf17	PHD finger protein 17	400.6165	536.7405	816.0015	545.0165
	270166	Clpx	caseinolytic peptidase X (E.coli)	410.013	270.65	179.9955	287.5955
	27878	Tada1l	transcriptional adaptor 1 (HFI1 homolog, yeast) like	536.889	398.147	164.559	309.522
	28253	Slco1b2	solute carrier organic anion transporter family, member 1b2	272.1693	210.7363	1454.75	988.548
	286940	Flnb	filamin, beta	257.6105	199.5535	118.3255	231.784
	29813	Zfp385a	zinc finger protein 385A	1411.235	805.237	651.595	628.6135
	29819	Stau2	staufen (RNA binding protein) homolog 2 (Drosophila)	469.3405	384.855	214.935	395.8675
	319387	Lphn3	latrophilin 3	605.6435	445.0125	339.8495	460.5965
	319653	Slc25a40	solute carrier family 25, member 40	905.183	509.716	466.15	589.0655
	319922	Vwc2	von Willebrand factor C domain containing 2	581.3425	312.2945	242.907	549.6165
	320374	7530414M10Rik	RIKEN cDNA 7530414M10 gene	86.00255	94.09375	223.8385	115.63
	320731	A530030E21Rik	RIKEN cDNA A530030E21 gene	70.61105	48.4799	39.06715	53.3569
	320926	C730029A08Rik	RIKEN cDNA C730029A08 gene	439.992	434.7005	996.9915	1045.411
	320988	B930096M23Rik	RIKEN cDNA B930096M23 gene	262.497	185.6055	76.4179	160.832
	328329	Mast4	microtubule associated serine/threonine kinase family member 4	149.176	139.534	67.9478	86.2524
	329739	Fam102b	family with sequence similarity 102, member B	657.4295	528.5735	326.1625	505.73
	347712	Pramel7	preferentially expressed antigen in melanoma like 7	210.347	156.593	104.5535	136.8985
	381110	Fam82a1	family with sequence similarity 82, member A1	173.9975	173.0395	307.437	178.675
	381598	6820431F20Rik	RIKEN cDNA 6820431F20 gene	229.8375	252.4045	637.6675	416.82
	381695	N4bp2l2	NEDD4 binding protein 2-like 2	19.6066	20.7011	58.8612	26.80265
	399568	BC052040	cDNA sequence BC052040	993.6425	650.7395	427.606	721.2505
	407823	Baz2b	bromodomain adjacent to zinc finger domain, 2B	140.573	103.5757	70.24885	120.2655
	50490	Nox4	NADPH oxidase 4	139.279	144.4725	330.2085	198.662
	50788	Fbxl8	F-box and leucine-rich repeat protein 8	743.7985	451.9465	301.7825	369.343
	52118	Pvr	poliovirus receptor	1851.45	1315.995	782.1395	1530.47
	52668	Ifi27l1	interferon, alpha-inducible protein 27 like 1	7335.11	4754.435	3529.815	3196.695
	52840	Dbndd2	dysbindin (dystrobrevin binding protein 1) domain containing 2	179.0335	137.0475	91.17365	116.8245
	53376	Usp2	ubiquitin specific peptidase 2	297.2545	313.026	764.0815	526.429
	53871	Pkd2l2	polycystic kidney disease 2-like 2	55.71565	54.81495	115.53	92.66585
	54123	Irf7	interferon regulatory factor 7	2271.05	1584.915	920.725	889.2035
	54196	Pabpn1	poly(A) binding protein, nuclear 1	4197.49	3002.58	1867.825	2157.755
	54342	Gnpnat1	glucosamine-phosphate N-acetyltransferase 1	606.4895	844.3965	1596.705	1282.24
	54357	Epb4.1l4b	erythrocyte protein band 4.1-like 4b	604.373	550.698	1343.205	940.834
	54635	Pdgfc	platelet-derived growth factor, C polypeptide	256.018	225.836	112.2019	162.929
	547253	Parp14	poly (ADP-ribose) polymerase family, member 14	1386.336	777.0295	438.874	667.957
	55932	Gbp3	guanylate binding protein 3	1434.595	798.1985	436.081	868.173
	56045	Samhd1	SAM domain and HD domain, 1	654.412	725.5295	247.7885	498.994
	56193	Plek	pleckstrin	233.813	235.292	97.89945	161.9175
	56362	Sult1b1	sulfotransferase family 1B, member 1	175.6005	209.7655	664.336	297.2315
	56469	Pias1	protein inhibitor of activated STAT 1	41.8074	43.02525	150.978	67.25605
	56524	Mpp6	membrane protein, palmitoylated 6 (MAGUK p55 subfamily member 6)	194.3525	120.849	63.4941	78.31505
	56551	Txn2	thioredoxin 2	2330.09	2300.84	1146.73	1626.475
	56619	Clec4e	C-type lectin domain family 4, member e	99.9886	129.48	50.3512	69.6197
	56747	Sez6l	seizure related 6 homolog like	90.89795	77.03515	253.6365	114.027
	57349	Ppbp	pro-platelet basic protein	226.078	221.0275	116.8175	186.19
	57748	Jmy	junction-mediating and regulatory protein	565.2445	384.4895	261.9695	378.5405
	58203	Zbp1	Z-DNA binding protein 1	745.127	695.5345	235.905	558.0725
	60345	Nrip2	nuclear receptor interacting protein 2	514.6095	389.821	233.646	297.69
	60533	Cd274	CD274 antigen	417.089	238.306	144.811	329.2045
	60599	Trp53inp1	transformation related protein 53 inducible nuclear protein 1	3151.705	4014.81	1687.26	2908.11
	64051	Sv2a	synaptic vesicle glycoprotein 2 a	889.8295	652.7985	393.1935	571.2925
	64209	Herpud1	homocysteine-inducible, endoplasmic reticulum stress-inducible, ubiquitin-like domain member 1	1499.82	2301.725	4611.965	3029.695
	64380	Ms4a4c	membrane-spanning 4-domains, subfamily A, member 4C	152.8605	167.7795	72.83155	85.92345
	66938	1700029G01Rik	RIKEN cDNA 1700029G01 gene	999.9325	631.0855	516.204	591.4655

Location	Entrez ID	Symbol	Gene Name	rAd.β-gal before	rAd.β-gal after	rAd.A20 before	rAd.A20 after
	66940	Shisa5	shisa homolog 5 (Xenopus laevis)	2747.76	2062.3	1340.605	1695.76
	67041	Oxct1	3-oxoacid CoA transferase 1	42.17065	33.0421	16.18045	17.9811
	67098	2210403K04Rik	RIKEN cDNA 2210403K04 gene	244.1435	286.318	467.333	440.544
	67120	Ttc14	tetratricopeptide repeat domain 14	209.453	216.4885	518.2515	333.985
	67135	2310021H06Rik	RIKEN cDNA 2310021H06 gene	576.667	429.5575	262.8885	362.351
	67177	Cdt1	chromatin licensing and DNA replication factor 1	975.203	1366.78	361.9545	763.8155
	67198	Spats2l	spermatogenesis associated, serine-rich 2-like	387.2	345.491	215.572	315.976
	67226	Tmem19	transmembrane protein 19	352.4775	277.697	202.178	263.6895
	672511	Rnf213	ring finger protein 213	3130.735	1826.395	1058.112	1653.935
	67337	Cstf1	cleavage stimulation factor, 3' pre-RNA, subunit 1	427.0995	324.581	197.393	340.858
	67358	1700093K21Rik	RIKEN cDNA 1700093K21 gene	33.63405	42.6522	76.5569	47.8372
	67452	Pnpla8	patatin-like phospholipase domain containing 8	926.12	1199.935	1811.02	1372.25
	67458	Ergic1	endoplasmic reticulum-golgi intermediate compartment (ERGIC) 1	265.2055	185.861	151.233	192.1015
	67554	Slc25a30	solute carrier family 25, member 30	96.66205	193.1595	311.8165	526.122
	677168	Gm9706	predicted gene 9706	562.758	423.874	181.441	287.0595
	67735	4930528A17Rik	RIKEN cDNA 4930528A17 gene	296.9025	210.991	145.338	177.943
	67739	Slc48a1	solute carrier family 48 (heme transporter), member 1	1977.73	1494.995	1007.463	1439.21
	67742	Samsn1	SAM domain, SH3 domain and nuclear localization signals, 1	104.4305	180.448	206.747	172.827
	67775	Rtp4	receptor transporter protein 4	3142.645	1740.515	1301.96	1708.887
	67834	Idh3a	isocitrate dehydrogenase 3 (NAD+) alpha	324.6355	326.214	983.254	505.7955
	67867	Lrrc28	leucine rich repeat containing 28	252.646	163.0855	119.237	201.081
	67878	Tmem33	transmembrane protein 33	31.2156	48.83485	72.0722	68.1197
	67969	2700080J24Rik	RIKEN cDNA 2700080J24 gene	615.0655	352.6365	207.517	335.451
	68292	Stt3b	STT3, subunit of the oligosaccharyltransferase complex, homolog B (S. cerevisiae)	255.359	226.1675	92.73285	152.569
	68468	Ly6g6c	lymphocyte antigen 6 complex, locus G6C	330.479	242.3575	174.269	179.914
	68728	Trp53inp2	transformation related protein 53 inducible nuclear protein 2	1407.61	2044.785	2374.185	2728.18
	68734	Smek1	SMEK homolog 1, suppressor of mek1 (Dictyostelium)	261.2165	182.8905	106.9805	193.7675
	68786	1110059G02Rik	RIKEN cDNA 1110059G02 gene	293.1	498.8245	804.287	612.425
	69179	Tmem110	transmembrane protein 110	900.756	657.2385	511.7175	571.677
	69276	Sec62	SEC62 homolog (S. cerevisiae)	428.4885	281.113	228.959	237.087
	69354	Slc38a4	solute carrier family 38, member 4	1095.816	1054.465	2163.815	2150.565
	69605	Lnp	limb and neural patterns	50.57645	52.4347	129.6875	72.0638
	69657	2310047D07Rik	RIKEN cDNA 2310047D07 gene	575.1125	423.1685	236.854	335.474
	69774	Ms4a6b	membrane-spanning 4-domains, subfamily A, member 6B	538.2295	591.032	226.5265	246.041
	69851	2010007E15Rik	RIKEN cDNA 2010007E15 gene	276.438	222.0565	148.33	178.225
	69893	2010305A19Rik	RIKEN cDNA 2010305A19 gene	91.9124	120.2495	171.8335	172.0745
	69918	2610020C07Rik	RIKEN cDNA 2610020C07 gene	158.791	120.787	79.89625	123.8285
	70078	Nol7	nucleolar protein 7	133.636	151.325	387.003	220.622
	70439	Taf15	TAF15 RNA polymerase II, TATA box binding protein (TBP)-associated factor	165.7915	144.6365	78.90285	98.4023
	70453	2610105M22Rik	RIKEN cDNA 2610105M22 gene	132.4065	132.622	247.594	151.6115
	70496	5730406E14Rik	RIKEN cDNA 5730406E14 gene	159.779	138.0145	69.1398	98.52715
	71101	4933407H18Rik	RIKEN cDNA 4933407H18 gene	289.982	210.139	136.5835	161.212
	71177	4933424B01Rik	RIKEN cDNA 4933424B01 gene	665.969	516.981	273.373	498.9645
	71341	5430434N17Rik	RIKEN cDNA 5430434N17 gene	75.74365	57.77565	33.5191	54.30245
	71363	Krtap7-1	keratin associated protein 7-1	280.515	225.8295	151.3405	165.629
	71365	Pdss2	prenyl (solanesyl) diphosphate synthase, subunit 2	184.023	123.551	75.68	98.74805
	71421	Amtn	amelotin	342.2425	289.5845	160.594	199.6075
	71769	Bbs10	Bardet-Biedl syndrome 10 (human)	461.6485	387.427	247.4655	353.1525
	71907	Serpina9	serine (or cysteine) peptidase inhibitor, clade A (alpha-1 antiproteinase, antitrypsin), member 9	169.5295	190.4105	399.585	286.3905
	71979	2410012M07Rik	RIKEN cDNA 2410012M07 gene	314.4475	207.5955	127.307	178.4845
	72084	Pigx	phosphatidylinositol glycan anchor biosynthesis, class X	491.169	743.4085	1317.64	712.609
	72124	Seh1l	SEH1-like (S. cerevisiae	450.7845	283.965	240.7485	288.363
	72215	1700001P01Rik	RIKEN cDNA 1700001P01 gene	378.453	312.707	178.26	286.7325
	72313	Fryl	furry homolog-like (Drosophila)	639.883	515.3695	308.3255	382.1865
	72773	2810449G22Rik	RIKEN cDNA 2810449G22 gene	406.029	301.5115	223.5235	235.465
	72804	9130017K11Rik	RIKEN cDNA 9130017K11 gene	325.42	276.295	122.9913	237.71
	73132	Slc25a16	solute carrier family 25 (mitochondrial carrier, Graves disease autoantigen), member 16	245.191	483.072	769.5505	557.657
	73174	Tbkbp1	TBK1 binding protein 1	747.0075	548.4225	370.944	517.0595
	73284	Ddit4l	DNA-damage-inducible transcript 4-like	390.8985	251.872	123.3985	155.8505
	73398	1700054F22Rik	RIKEN cDNA 1700054F22 gene	84.38465	91.00965	210.92	152.5805
	73442	Hspa12a	heat shock protein 12A	297.191	224.3955	168.6275	177.088
	73545	1700094D03Rik	RIKEN cDNA 1700094D03 gene	613.3785	483.0365	334.096	538.927
	73721	1110017D15Rik	RIKEN cDNA 1110017D15 gene	370.524	281.1805	178.367	222.341
	74088	0610012H03Rik	RIKEN cDNA 0610012H03 gene	396.804	492.1455	806.339	849.547
	74153	Ube1l	ubiquitin-activating enzyme E1-like	639.753	331.2935	232.2965	347.767
	74156	Acot12	acyl-CoA thioesterase 12	634.5465	851.6935	1571.665	1073.184
	74389	4932429P19Rik	RIKEN cDNA 4932429P19 gene	275.7855	231.1145	161.1595	210.795
	74558	Gvin1	GTPase, very large interferon inducible 1	1155.225	735.098	432.2375	701.1535
	74587	4833422B07Rik	RIKEN cDNA 4833422B07 gene	221.1655	158.049	94.16595	159.6995
	75388	Boll	bol, boule-like (Drosophila)	169.896	138.843	97.3716	115.45
	75472	1700009P17Rik	RIKEN cDNA 1700009P17 gene	715.7215	486.8855	356.7065	488.145
	75485	1700010B08Rik	RIKEN cDNA 1700010B08 gene	589.7495	434.169	264.2955	404.1655
	75710	Rbm12	RNA binding motif protein 12	494.989	326.278	228.4135	280.1195
	75723	Amotl1	angiomotin-like 1	627.4415	462.573	334.065	437.383
	75785	Klhl24	kelch-like 24 (Drosophila)	21.2618	29.25804	73.4449	58.8362
	75805	Nln	neurolysin (metallopeptidase M3 family)	129.65	169.3655	217.401	228.077
	76013	5830407E08Rik	RIKEN cDNA 5830407E08 gene	334.6405	286.2925	165.442	208.641

Location	Entrez ID	Symbol	Gene Name	rAd.β-gal before	rAd.β-gal after	rAd.A20 before	rAd.A20 after
	76093	5830469G19Rik	RIKEN cDNA 5830469G19 gene	161.832	167.77	65.58945	96.0054
	76118	5830487J09Rik	RIKEN cDNA 5830487J09 gene	101.9425	73.63865	39.93965	53.97615
	76184	Abca6	ATP-binding cassette, sub-family A (ABC1), member 6	146.983	256.862	345.553	499.497
	76464	Casc5	cancer susceptibility candidate 5	569.288	427.4945	293.028	413.8755
	77065	Ints7	integrator complex subunit 7	254.7495	220.7225	130.737	174.6685
	77337	Akr1c21	aldo-keto reductase family 1, member C21	203.3885	119.165	415.8215	220.0235
	77599	5830420C07Rik	RIKEN cDNA 5830420C07 gene	345.1175	221.341	164.186	299.1925
	77634	Snapc3	small nuclear RNA activating complex, polypeptide 3	116.927	126.8005	253.2765	161.875
	77652	Zfp422-rs1	zinc finger protein 422, related sequence 1	185.6165	127.5745	91.118	144.55
	78243	9230112D13Rik	RIKEN cDNA 9230112D13 gene	421.0835	344.6105	232.9945	273.62
	78257	Lrrc9	leucine rich repeat containing 9	164.1065	117.918	78.55205	99.478
	78715	D530015H24Rik	RIKEN cDNA D530015H24 gene	818.99	582.6595	340.82	450.8275
	78825	Pppde1	PPPDE peptidase domain containing 1	700.8965	644.6195	356.09	609.04
	78923	Chsy3	chondroitin sulfate synthase 3	79.1751	51.58735	33.65675	47.5858
	80290	Gpr146	G protein-coupled receptor 146	432.575	422.033	835.9555	582.214
	80517	Herpud2	HERPUD family member 2	226.188	215.5855	77.4022	117.4855
	80750	N4bp1	NEDD4 binding protein 1	536.97	379.5565	199.8875	300.5675
	80859	Nfkbiz	nuclear factor of kappa light polypeptide gene enhancer in B-cells inhibitor, zeta	292.4235	242.909	139.846	252.2445
	80890	Trim2	tripartite motif-containing 2	760.558	454.075	266.157	341.387
	83922	Tsga14	testis specific gene A14	207.4305	200.75	481.971	243.534
	94090	Trim9	tripartite motif-containing 9	400.7605	301.8135	180.083	254.5865
	94191	Adarb2	adenosine deaminase, RNA-specific, B2	430.082	293.906	211.969	274.8365
	96935	Susd4	sushi domain containing 4	172.1525	117.696	385.9425	261.5365
	96955	C77144	expressed sequence C77144	284.022	201.848	125.443	163.331
	97222	C77691	expressed sequence C77691	82.9981	58.39155	166.076	92.8989
	97383	C80278	expressed sequence C80278	154.5165	113.974	55.81705	98.3568
	97484	Cog8	component of oligomeric golgi complex 8	1980.245	1294.94	1019.346	1754.57
	98314	D2hgdh	D-2-hydroxyglutarate dehydrogenase	338.543	335.61	593.638	393.0225
	99686	AI606473	expressed sequence AI606473	260.9845	194.3745	142.61	193.052
	99887	Tmem56	transmembrane protein 56	24.29215	39.5798	77.64805	64.59195
	99899	Ifi44	interferon-induced protein 44	1444.515	578.1235	236.6425	412.189
	99946	6720422M22Rik	RIKEN cDNA 6720422M22 gene	98.11055	62.0192	51.67185	62.5803

**q13**	100336	Ppp1r8	protein phosphatase 1, regulatory (inhibitor) subunit 8	238.372	338.92	201.872	151.2585
	100340	Smpdl3b	sphingomyelin phosphodiesterase, acid-like 3B	209.559	406.3445	112.273	130.281
	101351	A130022J15Rik	RIKEN cDNA A130022J15 gene	136.7515	231.004	105.262	129.9355
	101602	AI467606	expressed sequence AI467606	122.7666	156.4945	84.73135	74.2694
	103149	Upb1	ureidopropionase, beta	2502.515	1555.295	3820.345	3355.335
	103266	AI597468	expressed sequence AI597468	476.2905	828.967	400.209	475.302
	103784	Wdr92	WD repeat domain 92	151.4757	162.802	137.379	76.784
	104776	Aldh6a1	aldehyde dehydrogenase family 6, subfamily A1	1899.365	1318.415	3341.725	2399.295
	104923	Adi1	acireductone dioxygenase 1	1621.58	819.3025	2098.605	1625.075
	106572	Rab31	RAB31, member RAS oncogene family	292.4135	580.032	213.3395	280.9785
	107513	Ssr1	signal sequence receptor, alpha	196.433	326.177	189.847	179.621
	107869	Cth	cystathionase (cystathionine gamma-lyase)	2633.12	1935.43	3618.71	4528.715
	108012	Ap1s2	adaptor-related protein complex 1, sigma 2 subunit	81.5739	139.5305	71.29755	69.6313
	108037	Shmt2	serine hydroxymethyltransferase 2 (mitochondrial)	1149.39	799.5405	1291.405	1366.1
	108062	Cstf2	cleavage stimulation factor, 3' pre-RNA subunit 2	593.549	902.234	508.4475	466.2175
	108101	Fermt3	fermitin family homolog 3 (Drosophila)	102.2729	160.988	68.7559	80.69545
	108138	Xrcc4	X-ray repair complementing defective repair in Chinese hamster cells 4	163.462	245.8855	177.5885	131.019
	108143	Taf9	TAF9 RNA polymerase II, TATA box binding protein (TBP)-associated factor	570.1715	768.0555	563.645	435.463
	108151	Sema3d	sema domain, immunoglobulin domain (Ig), short basic domain, secreted, (semaphorin) 3D	71.85585	62.7058	86.19665	105.721
	108671	Dnajc9	DnaJ (Hsp40) homolog, subfamily C, member 9	954.6875	1478.58	602.03	739.853
	108689	Obfc1	oligonucleotide/oligosaccharide-binding fold containing 1	276.108	336.34	149.032	163.2235
	108745	2310051F07Rik	RIKEN cDNA 2310051F07 gene	333.809	490.88	211.303	249.461
	108853	Mtrf1l	mitochondrial translational release factor 1-like	159.649	227.542	149.8245	120.3555
	109075	Exosc4	exosome component 4	727.722	1091.302	605.953	478.5885
	109229	Fam118b	family with sequence similarity 118, member B	74.38435	134.3085	100.4557	65.2173
	109674	Ampd2	adenosine monophosphate deaminase 2 (isoform L)	1016.995	1173.805	750.784	577.0135
	110208	Pgd	phosphogluconate dehydrogenase	2678.41	2373.035	788.2915	1061.359
	110796	Tshz1	teashirt zinc finger family member 1	195.542	330.008	230.0565	181.6195
	11487	Adam10	a disintegrin and metallopeptidase domain 10	570.6955	871.1175	457.6525	473.0155
	11669	Aldh2	aldehyde dehydrogenase 2, mitochondrial	295.631	194.586	347.888	364.1465
	11735	Ank3	ankyrin 3, epithelial	174.5965	111.245	289.17	249.148
	12175	Bnip2	BCL2/adenovirus E1B interacting protein 2	977.7525	1179.907	707.01	529.1955
	12332	Capg	capping protein (actin filament), gelsolin-like	156.9464	145.969	49.10705	79.6408
	12368	Casp6	caspase 6	384.0965	381.292	600.783	750.463
	12411	Cbs	cystathionine beta-synthase	2167.05	1187.13	1973.09	2534.365
	12448	Ccne2	cyclin E2	642.696	1166.06	451.5895	403.026
	12450	Ccng1	cyclin G1	1304.085	1778.295	879.2145	722.52
	12455	Ccnt1	cyclin T1	725.755	638.669	402.156	358.0565
	12499	Entpd5	ectonucleoside triphosphate diphosphohydrolase 5	656.9035	595.3815	981.18	1116.69
	12566	Cdk2	cyclin-dependent kinase 2	502.199	817.913	387.731	446.821
	12609	Cebpd	CCAAT/enhancer binding protein (C/EBP), delta	628.385	1347.185	364.614	684.759
	12724	Clcn2	chloride channel 2	231.9645	115.598	377.7655	265.913
	12727	Clcn4-2	chloride channel 4-2	275.509	329.3345	203.67	124.7415
	12780	Abcc2	ATP-binding cassette, sub-family C (CFTR/MRP), member 2	708.9695	516.872	734.2985	919.8805

Location	Entrez ID	Symbol	Gene Name	rAd.β-gal before	rAd.β-gal after	rAd.A20 before	rAd.A20 after
	12822	Col18a1	collagen, type XVIII, alpha 1	2949.305	1319.65	2647.595	2725.425
	12846	Comt1	catechol-O-methyltransferase 1	4957.545	3889.915	7569.315	6521.425
	12865	Cox7a1	cytochrome c oxidase, subunit VIIa 1	189.6865	195.659	177.4895	106.444
	12916	Crem	cAMP responsive element modulator	436.645	588.122	315.9925	325.2035
	13026	Pcyt1a	phosphate cytidylyltransferase 1, choline, alpha isoform	305.395	257.7005	455.154	620.642
	13144	Dapk3	death-associated protein kinase 3	352.743	473.408	315.7135	254.1185
	13171	Dbt	dihydrolipoamide branched chain transacylase E2	496.8325	278.097	836.051	633.731
	13437	Dnpep	aspartyl aminopeptidase	1056.23	1599.465	1211.19	851.149
	13589	Mapre1	microtubule-associated protein, RP/EB family, member 1	1179.9	1404.75	721.814	773.4665
	13660	Ehd1	EH-domain containing 1	630.026	739.1945	408.4785	378.214
	13728	Mark2	MAP/microtubule affinity-regulating kinase 2	395.103	447.663	234.2625	231.784
	14007	Cugbp2	CUG triplet repeat, RNA binding protein 2	210.5085	269.75	167.493	149.999
	14067	F5	coagulation factor V	1461.16	1212.11	2402.06	2189.42
	14073	Faah	fatty acid amide hydrolase	1231.17	759.7225	1626.45	1372.785
	140792	Colec12	collectin sub-family member 12	232.4415	206.658	345.2005	358.5245
	14081	Acsl1	acyl-CoA synthetase long-chain family member 1	1497.316	735.108	3868.335	3102.42
	140919	Slc17a6	solute carrier family 17 (sodium-dependent inorganic phosphate cotransporter), member 6	102.6468	96.3895	109.1715	174.6555
	14120	Fbp2	fructose bisphosphatase 2	329.423	471.444	184.9875	213.671
	14155	Fem1b	feminization 1 homolog b (C. elegans)	627.09	858.1875	474.7735	435.8355
	14302	Frk	fyn-related kinase	58.32215	103.1992	43.7483	41.0192
	14583	Gfpt1	glutamine fructose-6-phosphate transaminase 1	341.507	640.9745	348.5955	368.0345
	14651	Hagh	hydroxyacyl glutathione hydrolase	2862.595	1639.63	4383.24	2903.87
	14678	Gnai2	guanine nucleotide binding protein (G protein), alpha inhibiting 2	855.116	877.3215	441.3195	223.444
	14756	Gpld1	glycosylphosphatidylinositol specific phospholipase D1	1533.16	870.7385	1780.515	1758.36
	14874	Gstz1	glutathione transferase zeta 1 (maleylacetoacetate isomerase)	3443.36	2318.275	5453.535	4212.48
	14885	Gtf2h4	general transcription factor II H, polypeptide 4	612.137	866.4415	453.14	371.272
	15182	Hdac2	histone deacetylase 2	237.8575	307.374	194.822	177.9625
	15186	Hdc	histidine decarboxylase	134.765	198.7735	115.658	112.439
	15191	Hdgf	hepatoma-derived growth factor	1138.575	1108.235	751.1655	432.138
	15388	Hnrnpl	heterogeneous nuclear ribonucleoprotein L	921.558	1307.68	879.501	663.1815
	15445	Hpd	4-hydroxyphenylpyruvic acid dioxygenase	9407.43	5033.495	10861.25	10381.07
	15529	Sdc2	syndecan 2	1050.76	638.9535	1454.41	1486.365
	16179	Irak1	interleukin-1 receptor-associated kinase 1	511.9325	534.365	267.237	215.7745
	16188	Il3ra	interleukin 3 receptor, alpha chain	192.9335	187.802	105.8536	108.2725
	16325	Inhbc	inhibin beta-C	1235.582	880.6245	1710.21	1535.465
	16418	Eif6	eukaryotic translation initiation factor 6	2517.5	4746.275	1494.295	2259.83
	17022	Lum	lumican	161.358	155.3325	293.513	378.2785
	170442	Bbox1	butyrobetaine (gamma), 2-oxoglutarate dioxygenase 1 (gamma-butyrobetaine hydroxylase)	1775.755	1065.312	3678.34	2259.755
	17064	Cd93	CD93 antigen	448.946	703.2025	273.372	338.2325
	17112	Tm4sf1	transmembrane 4 superfamily member 1	350.1275	311.371	561.1985	524.803
	17299	Mettl1	methyltransferase like 1	211.9575	392.19	165.511	123.0195
	17756	Mtap2	microtubule-associated protein 2	133.9215	243.737	99.67635	129.718
	17863	Myb	myeloblastosis oncogene	57.1143	72.3675	28.5727	26.29835
	17886	Myh9	myosin, heavy polypeptide 9, non-muscle	533.0055	639.9365	272.8315	341.3205
	17972	Ncf4	neutrophil cytosolic factor 4	301.168	440.662	153.0445	200.9535
	18003	Nedd9	neural precursor cell expressed, developmentally down-regulated gene 9	176.67	168.5475	65.89995	93.384
	18176	Nras	neuroblastoma ras oncogene	280.63	386.0085	234.198	222.3685
	18242	Oat	ornithine aminotransferase	2655.405	2867.12	5060.44	8406.925
	18452	P4ha2	procollagen-proline, 2-oxoglutarate 4-dioxygenase (proline 4-hydroxylase), alpha II polypeptide	273.3945	441.6255	225.438	267.9335
	18536	Pcm1	pericentriolar material 1	100.7195	166.22	70.08235	91.36245
	18537	Pcmt1	protein-L-isoaspartate (D-aspartate) O-methyltransferase 1	650.6055	814.869	704.5755	450.372
	18582	Pde6d	phosphodiesterase 6D, cGMP-specific, rod, delta	217.9395	290.155	180.1635	160.415
	18607	Pdpk1	3-phosphoinositide dependent protein kinase-1	233.718	244.8	113.8772	72.88455
	18643	Pfn1	profilin 1	4461.98	5391.205	3068.72	3288.15
	18813	Pa2g4	proliferation-associated 2G4	605.0245	836.712	410.336	363.1055
	19013	Ppara	peroxisome proliferator activated receptor alpha	771.97	736.86	1917.495	1624.225
	19122	Prnp	prion protein	620.9005	404.3125	912.763	745.0105
	19125	Prodh	proline dehydrogenase	1024.014	483.44	1183.11	1327.27
	192156	Mvd	mevalonate (diphospho) decarboxylase	196.852	386.3625	183.722	122.8435
	19290	Pura	purine rich element binding protein A	654.27	624.2525	527.8985	296.122
	19348	Kif20a	kinesin family member 20A	125.1552	125.71	143.7185	229.731
	19651	Rbl2	retinoblastoma-like 2	183.7075	135.982	409.227	290.5525
	19656	Rbmxrt	RNA binding motif protein, X chromosome retrogene	699.522	909.876	524.265	511.902
	19659	Rbp1	retinol binding protein 1, cellular	389.851	167.999	307.8025	293.446
	19720	Trim27	tripartite motif-containing 27	728.1865	994.153	526.939	499.512
	19733	Rgn	regucalcin	4135.505	2618.82	7015.77	5505.12
	20016	Polr1c	polymerase (RNA) I polypeptide C	452.032	726.7565	424.7125	348.46
	20411	Sorbs1	sorbin and SH3 domain containing 1	546.4185	504.396	464.26	165.276
	20419	Shcbp1	Shc SH2-domain binding protein 1	320.3805	265.039	312.252	622.6525
	20493	Slc10a1	solute carrier family 10 (sodium/bile acid cotransporter family), member 1	1185.914	832.5765	3906.43	3025.365
	20511	Slc1a2	solute carrier family 1 (glial high affinity glutamate transporter), member 2	328.1435	270.028	530.794	535.6545
	20655	Sod1	superoxide dismutase 1, soluble	619.1835	316.4945	621.292	855.542
	20656	Sod2	superoxide dismutase 2, mitochondrial	407.937	640.8715	532.7945	337.8745
	208146	Yeats2	YEATS domain containing 2	161.9902	148.2335	83.70855	64.1473
	208292	Zfp871	zinc finger protein 871	128.846	111.5233	270.163	362.5165

Location	Entrez ID	Symbol	Gene Name	rAd.β-gal before	rAd.β-gal after	rAd.A20 before	rAd.A20 after
	208665	Akr1d1	aldo-keto reductase family 1, member D1	745.33	289.5195	1292.4	1030.895
	20928	Abcc9	ATP-binding cassette, sub-family C (CFTR/MRP), member 9	326.001	309.123	452.3615	517.445
	209692	Dhtkd1	dehydrogenase E1 and transketolase domain containing 1	1206.94	682.5115	1470.26	1315.395
	212647	Aldh4a1	aldehyde dehydrogenase 4 family, member A1	2094.68	963.1455	3050.68	2359.515
	214359	Tmem51	transmembrane protein 51	620.948	678.2115	297.9915	368.4565
	215708	Fam73a	family with sequence similarity 73, member A	131.3135	190.2405	109.9416	96.0339
	216965	Taok1	TAO kinase 1	431.746	450.363	285.6935	240.7225
	217333	Trim47	tripartite motif-containing 47	631.0445	854.667	224.263	330.8945
	21750	Terf2	telomeric repeat binding factor 2	297.933	348.283	190.889	162.9895
	21915	Dtymk	deoxythymidylate kinase	1024.354	1346.21	631.748	553.9835
	21927	Tnfaip1	tumor necrosis factor, alpha-induced protein 1 (endothelial)	828.7675	1173.31	431.952	591.826
	21985	Tpd52	tumor protein D52	779.5225	1595.065	625.911	886.407
	22032	Traf4	TNF receptor associated factor 4	559.91	671.3505	430.6215	380.18
	22099	Tsn	translin	987.3185	1205.65	691.7865	449.1435
	22129	Ttc3	tetratricopeptide repeat domain 3	347.3615	373.3155	281.317	195.4705
	22137	Ttk	Ttk protein kinase	203.9565	157.3705	184.5715	296.449
	22192	Ube2m	ubiquitin-conjugating enzyme E2M (UBC12 homolog, yeast)	584.227	814.119	462.8195	369.72
	22361	Vnn1	vanin 1	338.1835	388.703	215.5825	202.2165
	22401	Zmat3	zinc finger matrin type 3	514.582	747.6415	255.0205	314.7635
	225160	Thoc1	THO complex 1	156.838	235.47	139.385	102.6021
	225631	Onecut2	one cut domain, family member 2	205.177	158.643	277.853	328.1295
	227333	Dgkd	diacylglycerol kinase, delta	332.972	535.8155	236.313	243.8325
	227638	Qsox2	quiescin Q6 sulfhydryl oxidase 2	190.057	290.2665	173.067	169.386
	227746	Rabepk	Rab9 effector protein with kelch motifs	175.5935	315.012	195.481	156.2425
	227753	Gsn	gelsolin	322.261	185.2095	167.033	97.4166
	227835	Gtdc1	glycosyltransferase-like domain containing 1	544.403	828.524	283.3235	364.441
	228368	Slc35c1	solute carrier family 35, member C1	460.7235	528.5395	261.915	312.597
	229317	Eif2a	eukaryotic translation initiation factor 2a	108.4987	201.152	138.9845	103.787
	230857	Ece1	endothelin converting enzyme 1	1497.905	772.5645	1640.35	2202.8
	231642	Alkbh2	alkB, alkylation repair homolog 2 (E. coli)	359.8555	526.287	205.5535	228.6705
	231691	Sds	serine dehydratase	2479.09	2618.575	3706.545	4745.18
	231872	Aimp2	aminoacyl tRNA synthetase complex-interacting multifunctional protein 2	739.299	1301.97	759.868	662.5665
	233908	Fus	fusion, derived from t(12;16) malignant liposarcoma (human)	1196.83	2028.105	560.4135	1119.915
	234358	D10627	cDNA sequence D10627	100.9299	138.4875	76.82725	75.83265
	234725	Zfp612	zinc finger protein 612	76.74875	98.197	50.9554	39.68675
	235534	Acpl2	acid phosphatase-like 2	388.321	462.5225	203.0205	229.3325
	238055	Apob	apolipoprotein B	1177.645	2073.47	2738.535	6304.005
	23874	Farsb	phenylalanyl-tRNA synthetase, beta subunit	720.536	1107.438	569.672	500.2455
	238831	Ppwd1	peptidylprolyl isomerase domain and WD repeat containing 1	229.354	298.8815	178.8185	157.8025
	23999	Twf2	twinfilin, actin-binding protein, homolog 2 (Drosophila)	126.6055	196.203	71.95625	78.8315
	24059	Slco2a1	solute carrier organic anion transporter family, member 2a1	416.612	345.5265	468.734	724.9465
	240638	Slc16a12	solute carrier family 16 (monocarboxylic acid transporters), member 12	507.8575	321.6365	1081.665	755.8845
	241226	Itga8	integrin alpha 8	112.7605	66.40355	161.2135	137.0145
	241490	Rbm45	RNA binding motif protein 45	377.2465	773.639	362.4695	343.946
	243382	Ppm1k	protein phosphatase 1K (PP2C domain containing)	829.8555	597.8995	1544.025	1147.215
	245404	Decaf12l1	DDB1 and CUL4 associated factor 12-like 1	48.3109	38.4741	27.3237	9.788365
	245631	Mum1l1	melanoma associated antigen (mutated) 1-like 1	55.27255	129.117	44.02535	41.77505
	245841	Polr2h	polymerase (RNA) II (DNA directed) polypeptide H	373.555	644.7865	471.7585	352.612
	257635	Sdsl	serine dehydratase-like	988.4165	2223.875	544.938	634.681
	26378	Decr2	2-4-dienoyl-Coenzyme A reductase 2, peroxisomal	1070.421	923.9355	1931.59	1855.095
	26458	Slc27a2	solute carrier family 27 (fatty acid transporter), member 2	4405.185	3929.115	8003.835	7178.35
	268860	Abat	4-aminobutyrate aminotransferase	990.593	519.2715	1864.43	1447.825
	269113	Nup54	nucleoporin 54	358.866	466.0835	244.5955	247.769
	27015	Polk	polymerase (DNA directed), kappa	376.1945	379.8035	185.559	207.127
	27357	Gyg	glycogenin	678.982	1029.133	489.4035	551.4925
	27376	Slc25a10	solute carrier family 25 (mitochondrial carrier, dicarboxylate transporter), member 10	1104.204	785.0645	1630.7	1600.26
	28036	Larp7	La ribonucleoprotein domain family, member 7	470.9475	523.292	294.9645	285.6
	280662	Afm	afamin	2068.555	1333.45	3269.815	2404.385
	30795	Fkbp3	FK506 binding protein 3	809.4265	1128.525	769.808	556.525
	30926	Glrx3	glutaredoxin 3	1905.72	1712.51	1563.97	1025.335
	317758	Gimap9	GTPase, IMAP family member 9	198.4875	311.385	158.4245	135.7565
	319190	Hist2h2be	histone cluster 2, H2be	94.77605	41.00285	101.3431	92.27975
	319263	Pcmtd1	protein-L-isoaspartate (D-aspartate) O-methyltransferase domain containing 1	426.5815	356.8455	881.096	866.3745
	319660	Tmem195	transmembrane protein 195	954.5865	554.241	1908.4	1351.735
	319801	9630033F20Rik	RIKEN cDNA 9630033F20 gene	279.6485	456.0185	274.072	213.149
	319953	Ttll1	tubulin tyrosine ligase-like 1	113.7744	141.214	82.1999	57.65415
	320165	Tacc1	transforming, acidic coiled-coil containing protein 1	276.21	426.1525	248.9	238.7605
	320267	Fubp3	far upstream element (FUSE) binding protein 3	325.952	514.2375	257.417	260.619
	320708	B430007K19Rik	RIKEN cDNA B430007K19 gene	66.3952	68.05085	35.51905	36.74925
	327762	Dna2	DNA replication helicase 2 homolog (yeast)	456.258	796.637	356.009	371.8225
	328035	Fads6	fatty acid desaturase domain family, member 6	951.8235	613.3525	1031	1057.115
	331026	Gmppb	GDP-mannose pyrophosphorylase B	452.4915	1060.459	490.01	338.801
	338359	Supv3l1	suppressor of var1, 3-like 1 (S. cerevisiae)	424.91	667.3965	450.6015	386.5835
	380601	Fastkd5	FAST kinase domains 5	400.44	606.0565	466.8315	302.563
	402765	6030422H21Rik	RIKEN cDNA 6030422H21 gene	54.4808	89.54245	51.4715	37.06865
	433247	Cyp2c68	cytochrome P450, family 2, subfamily c, polypeptide 68	1803.615	1253.39	4294.01	2558.455
	50492	Thop1	thimet oligopeptidase 1	254.6	491.7375	181.1035	191.269

Location	Entrez ID	Symbol	Gene Name	rAd.β-gal before	rAd.β-gal after	rAd.A20 before	rAd.A20 after
	50708	Hist1h1c	histone cluster 1, H1c	1820.725	1043.224	2022.44	2294.495
	50793	Orc3l	origin recognition complex, subunit 3-like (S. cerevisiae)	372.1385	575.0465	395.756	346.1055
	50799	Slc25a13	solute carrier family 25 (mitochondrial carrier, adenine nucleotide translocator), member 13	1074.345	1001.671	2044.94	2335.685
	50926	Hnrpdl	heterogeneous nuclear ribonucleoprotein D-like	262.9475	484.6825	234.1965	249.98
	51886	Fubp1	far upstream element (FUSE) binding protein 1	98.12915	150.9225	61.72085	74.9528
	52206	Anapc4	anaphase promoting complex subunit 4	183.3575	336.6955	145.4065	139.279
	52585	Dhrs1	dehydrogenase/reductase (SDR family) member 1	1472.695	1100.035	1940.415	2071.005
	52637	Cisd1	CDGSH iron sulfur domain 1	1777.985	1031.506	2582.135	2146.215
	52710	Gpr172b	G protein-coupled receptor 172B	207.1675	403.4225	142.774	147.7605
	53623	Gria3	glutamate receptor, ionotropic, AMPA3 (alpha 3)	63.1246	68.7864	35.6102	35.21385
	53902	Rcan3	regulator of calcineurin 3	77.98965	131.997	58.23615	67.4619
	54208	Arl6ip1	ADP-ribosylation factor-like 6 interacting protein 1	2398.145	1371.22	3056.57	3417.92
	54369	Nme6	non-metastatic cells 6, protein expressed in (nucleoside-diphosphate kinase)	581.818	845.867	557.2745	416.9465
	54401	Ywhab	tyrosine 3-monooxygenase/tryptophan 5-monooxygenase activation protein, beta polypeptide	530.54	655.4465	395.407	363.176
	54484	Mkrn1	makorin, ring finger protein, 1	359.6965	437.317	149.787	228.9085
	56086	Set	SET translocation	2921.61	3295.755	1766.665	1617.725
	56173	Cldn14	claudin 14	374.2585	730.919	240.4135	334.0105
	56490	Zbtb20	zinc finger and BTB domain containing 20	360.0905	276.154	497.086	612.9715
	56636	Fgf21	fibroblast growth factor 21	308.218	541.845	234.6405	288.027
	56724	Cript	cysteine-rich PDZ-binding protein	56.52965	82.039	58.2766	38.68645
	56771	Med20	mediator complex subunit 20	473.852	728.486	394.829	352.863
	56794	Hacl1	2-hydroxyacyl-CoA lyase 1	2790.905	1418.37	3143.175	3183.22
	57259	Tob2	transducer of ERBB2, 2	304.5765	468.847	196.7615	228.377
	58244	Stx6	syntaxin 6	220.5685	247.4875	139.62	124.3175
	58521	Eid1	EP300 interacting inhibitor of differentiation 1	332.111	385.6875	151.6035	216.2945
	58809	Rnase4	ribonuclease, RNase A family 4	5238.48	4311.92	7839.69	8629.915
	59069	Tpm3	tropomyosin 3, gamma	1195.679	1549.09	676.444	801.126
	60315	Myg1	melanocyte proliferating gene 1	824.471	1182.405	674.0075	566.609
	620417	Gm6150	predicted gene 6150	147.2305	192.553	113.1435	106.3535
	66111	Tmed3	transmembrane emp24 domain containing 3	924.0215	1458.195	730.129	510.8595
	66208	Nenf	neuron derived neurotrophic factor	573.8155	645.329	432.195	349.068
	66229	Rpl7l1	ribosomal protein L7-like 1	683.353	859.687	690.052	492.3155
	66235	Eif1ay	eukaryotic translation initiation factor 1A, Y-linked	1135.836	1669.45	1067.31	879.0665
	66540	Fam107b	family with sequence similarity 107, member B	291.534	272.483	533.45	600.793
	66580	Esf1	ESF1, nucleolar pre-rRNA processing protein, homolog (S. cerevisiae)	323.6395	382.61	191.107	193.2855
	66661	Srp72	signal recognition particle 72	726.888	861.328	494.6125	280.7995
	666737	4632427E13Rik	RIKEN cDNA 4632427E13 gene	234.825	236.9845	125.412	122.469
	66901	Proz	protein Z, vitamin K-dependent plasma glycoprotein	632.435	335.73	1275.035	1025.254
	66905	Plin3	perilipin 3	1111.882	1784.19	836.807	741.099
	66910	Tmem107	transmembrane protein 107	209.032	376.716	241.0785	146.1695
	66923	Pbrm1	polybromo 1	637.761	763.441	466.8885	319.579
	66964	Golt1b	golgi transport 1 homolog B (S. cerevisiae)	88.61395	165.741	68.7226	74.9981
	67036	Mrpl45	mitochondrial ribosomal protein L45	816.123	937.304	497.0275	356.4475
	67154	Mtdh	metadherin	1424.51	1436.17	862.1655	795.8355
	67225	Rnpc3	RNA-binding region (RNP1, RRM) containing 3	68.9616	114.75	58.83065	59.32715
	67245	Peli1	pellino 1	248.811	262.3105	213.6155	143.8245
	67278	2900092E17Rik	RIKEN cDNA 2900092E17 gene	423.341	538.373	286.0835	285.455
	673094	Cd99	CD99 antigen	860.829	945.957	783.8825	540.7475
	67434	Ankrd33b	ankyrin repeat domain 33B	323.2685	185.2905	279.316	336.431
	67513	2610002J02Rik	RIKEN cDNA 2610002J02 gene	388.4825	561.5885	162.9945	229.821
	67604	1110007L15Rik	RIKEN cDNA 1110007L15 gene	547.6885	1038.661	631.195	554.0745
	67664	Rnf125	ring finger protein 125	2213.58	636.476	1910.475	1845.91
	67701	Wfdc2	WAP four-disulfide core domain 2	657.58	617.604	412.8335	276.417
	67711	Nsmce1	non-SMC element 1 homolog (S. cerevisiae)	473.5115	740.3205	430.2085	367.721
	67832	Bxdc2	brix domain containing 2	97.49445	140.2305	86.49405	68.0377
	67956	Setd8	SET domain containing (lysine methyltransferase) 8	642.64	615.2425	453.3275	349.394
	67967	Pold3	polymerase (DNA-directed), delta 3, accessory subunit	205.945	253.415	110.9454	100.7485
	68001	1110004E09Rik	RIKEN cDNA 1110004E09 gene	259.2065	390.4115	279.208	227.024
	68106	Nt5c3l	5'-nucleotidase, cytosolic III-like	193.9875	270.05	173.6455	108.2153
	68153	Gtf2e2	general transcription factor II E, polypeptide 2 (beta subunit)	395.534	633.5855	376.743	342.1785
	68278	Ddx39	DEAD (Asp-Glu-Ala-Asp) box polypeptide 39	766.6075	1136.975	453.148	528.464
	68404	Nrn1	neuritin 1	2130.365	828.0365	2726.7	2173.77
	68480	1110007C09Rik	RIKEN cDNA 1110007C09 gene	350.381	591.104	300.848	368.249
	68646	1110020G09Rik	RIKEN cDNA 1110020G09 gene	340.4225	205.8465	681.548	593.547
	68837	Foxk2	forkhead box K2	290.093	473.198	270.4205	242.138
	68988	Prpf31	PRP31 pre-mRNA processing factor 31 homolog (yeast)	372.751	564.3445	234.5325	268.4835
	69053	1810013L24Rik	RIKEN cDNA 1810013L24 gene	311.287	320.44	437.104	531.1205
	69072	Ebna1bp2	EBNA1 binding protein 2	444.272	695.867	396.3855	362.7515
	69113	Alkbh3	alkB, alkylation repair homolog 3 (E. coli)	820.875	1189.87	734.7615	610.5625
	69178	Snx5	sorting nexin 5	616.1965	974.93	482.0555	482.9875
	69617	Pitrm1	pitrilysin metallepetidase 1	169.5475	186.3725	194.397	104.7861
	69719	Cad	carbamoyl-phosphate synthetase 2, aspartate transcarbamylase, and dihydroorotase	240.015	490.5855	184.893	200.5245
	69792	Med6	mediator of RNA polymerase II transcription, subunit 6 homolog (yeast)	131.5295	186.1275	126.7916	103.515
	70120	Yars2	tyrosyl-tRNA synthetase 2 (mitochondrial)	233.3225	392.8125	332.2725	226.3545
	70237	Bhlhb9	basic helix-loop-helix domain containing, class B9	121.667	218.3705	79.26415	103.2945
	70335	Reep6	receptor accessory protein 6	3380.27	2353.09	4974.625	5560.05

Location	Entrez ID	Symbol	Gene Name	rAd.β-gal before	rAd.β-gal after	rAd.A20 before	rAd.A20 after
	70359	Gtpbp3	GTP binding protein 3	239.55	317.33	180.7595	174.67
	70451	Dhrs13	dehydrogenase/reductase (SDR family) member 13	472.415	916.0315	297.425	319.8785
	70620	Ube2v2	ubiquitin-conjugating enzyme E2 variant 2	985.396	1617.535	823.371	962.0035
	70678	3021401C12Rik	RIKEN cDNA 3021401C12 gene	146.491	90.63635	168.422	183.741
	71175	Nipbl	Nipped-B homolog (Drosophila)	407.13	405.699	234.895	190.0165
	71514	Sfpq	splicing factor proline/glutamine rich (polypyrimidine tract binding protein associated)	148.8805	255.829	115.727	137.8825
	71653	4930506M07Rik	RIKEN cDNA 4930506M07 gene	503.8555	825.5135	379.825	404.369
	71701	Pnpt1	polyribonucleotide nucleotidyltransferase 1	291.4315	455.15	230.9635	249.267
	71703	Armcx3	armadillo repeat containing, X-linked 3	108.2739	183.504	68.06485	73.95365
	71755	Dhdh	dihydrodiol dehydrogenase (dimeric)	264.8265	195.8345	610.9785	489.8835
	71756	Cpn2	carboxypeptidase N, polypeptide 2	2054.84	927.042	2150.24	2219.54
	71844	Nupl1	nucleoporin like 1	425.6555	672.241	203.091	244.8555
	71911	Bdh1	3-hydroxybutyrate dehydrogenase, type 1	813.8805	622.9165	1453.3	1477.775
	72057	Phf10	PHD finger protein 10	711.949	1072.84	609.4265	538.08
	72108	Ddhd2	DDHD domain containing 2	549.9055	704.3365	511.1155	336.3395
	72183	Snx6	sorting nexin 6	236.6125	307.2875	166.9425	142.47
	72269	Cda	cytidine deaminase	409.154	828.758	230.073	346.5675
	72482	Acbd6	acyl-Coenzyme A binding domain containing 6	262.4855	399.435	203.6805	196.445
	72658	2700097O09Rik	RIKEN cDNA 2700097O09 gene	264.135	404.3725	245.5	214.5605
	72938	Hspb11	heat shock protein family B (small), member 11	240.972	387.1525	270.952	165.9
	73569	Vgll3	vestigial like 3 (Drosophila)	87.26535	74.6928	110.595	133.84
	73658	Spns1	spinster homolog 1 (Drosophila)	898.1145	1308.035	732.0435	695.432
	73699	Ppp2r1b	protein phosphatase 2 (formerly 2A), regulatory subunit A (PR 65), beta isoform	294.61	437.08	262.754	256.5215
	74129	Dmgdh	dimethylglycine dehydrogenase precursor	2081.745	1826.185	2485.01	3251.5
	74132	Rnf6	ring finger protein (C3H2C3 type) 6	719.9805	472.349	281.17	208.2355
	74246	Gale	galactose-4-epimerase, UDP	1115.455	2041.61	559.6835	823.6645
	74451	Pgs1	phosphatidylglycerophosphate synthase 1	269.3455	452.6865	182.9195	225.5915
	74559	Elovl7	ELOVL family member 7, elongation of long chain fatty acids (yeast)	93.59185	172.84	64.6622	68.51175
	74718	Snx16	sorting nexin 16	302.1445	454.4095	229.7385	210.897
	74747	Ddit4	DNA-damage-inducible transcript 4	544.96	1208.73	289.557	407.186
	74838	Narg1	NMDA receptor-regulated gene 1	395.2575	665.745	321.889	382.5285
	75625	Mageh1	melanoma antigen, family H, 1	114.4759	252.303	123.0275	118.6675
	75627	Snapc1	small nuclear RNA activating complex, polypeptide 1	252.029	377.9255	215.1325	205.18
	75735	Pank1	pantothenate kinase 1	1716.425	898.604	2581.38	1791.475
	76257	Slc38a3	solute carrier family 38, member 3	4180.945	3379.455	6719.645	6380.08
	76279	Cyp2d26	cytochrome P450, family 2, subfamily d, polypeptide 26	5095.235	2742.765	6160.565	6124.695
	76650	Srxn1	sulfiredoxin 1 homolog (S. cerevisiae)	1342.502	1785.715	472.6355	757.1975
	76740	Efr3a	EFR3 homolog A (S. cerevisiae)	463.73	485.568	422.289	275.0615
	78388	Mvp	major vault protein	375.1695	695.3115	174.248	288.013
	78925	Srd5a1	steroid 5 alpha-reductase 1	508.9205	260.478	904.977	869.0115
	83921	Tmem2	transmembrane protein 2	259.45	523.9135	218.435	268.9705
	93697	Narg2	NMDA receptor-regulated gene 2	347.031	344.9415	265.786	173.666
	93726	Ear11	eosinophil-associated, ribonuclease A family, member 11	392.4575	633.363	305.884	315.864
	94061	Mrpl1	mitochondrial ribosomal protein L1	422.352	756.8305	735.7755	413.243
	94175	Hrg	histidine-rich glycoprotein	5673.385	3893.515	7211.53	6825.14
	94181	Nans	N-acetylneuraminic acid synthase (sialic acid synthase)	395.4425	694.6375	246.7675	263.1505
	94279	Sfxn2	sideroflexin 2	868.5085	325.3415	709.004	663.267
	97073	C79452	expressed sequence C79452	248.8145	198.337	236.784	337.3005

**q14**	106052	Fbxo4	F-box protein 4	226.317	473.498	264.808	254.2545
	106648	Cyp4f15	cytochrome P450, family 4, subfamily f, polypeptide 15	1603.945	573.693	1800.99	1554.765
	106957	Slc39a6	solute carrier family 39 (metal ion transporter), member 6	125.6265	278.997	84.8228	126.6135
	108156	Mthfd1	methylenetetrahydrofolate dehydrogenase (NADP+ dependent), methenyltetrahydrofolate cyclohydrolase, formyltetrahydrofolate synthase	1980.34	1040.055	1841.01	1760.06
	108673	Ccdc86	coiled-coil domain containing 86	263.651	510.469	224.0695	282.2145
	109129	Mmadhc	methylmalonic aciduria (cobalamin deficiency) cblD type, with homocystinuria	1676.845	3176.675	1738.23	1902.155
	114249	Npnt	nephronectin	147.3105	299.2595	104.5765	169.012
	11522	Adh1	alcohol dehydrogenase 1 (class I)	11693.2	5636.65	12186.3	11745.4
	11555	Adrb2	adrenergic receptor, beta 2	89.1406	399.153	68.7311	114.2545
	116852	Akr1c20	aldo-keto reductase family 1, member C20	2768.28	799.3725	3155.655	2310.575
	11690	Alox5ap	arachidonate 5-lipoxygenase activating protein	239.1895	524.124	176.936	268.9965
	11739	Slc25a4	solute carrier family 25 (mitochondrial carrier, adenine nucleotide translocator), member 4	214.6745	611.6505	129.9055	246.6415
	11847	Arg2	arginase type II	38.48205	198.064	25.24305	39.7333
	11997	Akr1b7	aldo-keto reductase family 1, member B7	79.4792	795.318	55.248	97.58785
	12390	Cav2	caveolin 2	124.6961	467.6255	212.0255	184.883
	12593	Cdyl	chromodomain protein, Y chromosome-like	138.7585	257.5175	118.9335	125.953
	12663	Chml	choroideremia-like	85.97405	162.6885	51.5222	60.83535
	12702	Socs3	suppressor of cytokine signaling 3	300.6735	983.3845	249.214	497.207
	12962	Crybb3	crystallin, beta B3	179.0525	569.013	188.1175	177.54
	12984	Csf2rb2	colony stimulating factor 2 receptor, beta 2, low-affinity (granulocyte-macrophage)	120.7415	242.1355	69.75985	115.3399
	13088	Cyp2b10	cytochrome P450, family 2, subfamily b, polypeptide 10	974.695	3001.8	713.055	874.558
	13109	Cyp2j5	cytochrome P450, family 2, subfamily j, polypeptide 5	895.0315	370.596	1172.26	815.3655
	13121	Cyp51	cytochrome P450, family 51	181.988	449.225	248.801	217.295
	13124	Cyp8b1	cytochrome P450, family 8, subfamily b, polypeptide 1	3357.545	1154.707	3612.745	2962.055

Location	Entrez ID	Symbol	Gene Name	rAd.β-gal before	rAd.β-gal after	rAd.A20 before	rAd.A20 after
	13211	Dhx9	DEAH (Asp-Glu-Ala-His) box polypeptide 9	826.981	1415.775	719.142	864.599
	14245	Lpin1	lipin 1	444.88	1619.175	725.529	533.117
	14261	Fmo1	flavin containing monooxygenase 1	2338.55	832.8485	3112.3	2121.635
	14313	Fst	follistatin	165.0695	406.6565	110.7145	147.9785
	15160	Serpind1	serine (or cysteine) peptidase inhibitor, clade D, member 1	3915.555	1269.285	4958.7	4375.52
	15251	Hif1a	hypoxia inducible factor 1, alpha subunit	976.414	1957.15	849.6255	1050.995
	15357	Hmgcr	3-hydroxy-3-methylglutaryl-Coenzyme A reductase	174.098	546.9185	244.972	200.9835
	15463	Agfg1	ArfGAP with FG repeats 1	119.383	231.567	98.76345	124.7955
	16178	Il1r2	interleukin 1 receptor, type II	104.9461	428.221	87.11225	97.547
	16413	Itgb1bp1	integrin beta 1 binding protein 1	288.2025	498.082	266.276	199.2685
	16525	Kcnk1	potassium channel, subfamily K, member 1	146.709	518.9725	176.342	144.1375
	16621	Klkb1	kallikrein B, plasma 1	2631.8	502.1375	3143.12	1809.95
	16847	Lepr	leptin receptor	31.38695	338.908	30.31585	27.5263
	170768	Pfkfb3	6-phosphofructo-2-kinase/fructose-2,6-biphosphatase 3	137.4625	413.951	191.2445	141.0955
	171180	Syt12	synaptotagmin XII	108.7735	655.537	113.4675	224.261
	17356	Mllt4	myeloid/lymphoid or mixed-lineage leukemia (trithorax homolog, Drosophila); translocated to, 4	344.0305	662.933	348.4175	373.8385
	17394	Mmp8	matrix metallopeptidase 8	152.8325	422.3765	127.2105	136.3775
	18416	Otc	ornithine transcarbamylase	118.422	51.39505	185.4465	103.8408
	18432	Mybbp1a	MYB binding protein (P160) 1a	328.6305	768.1545	366.9605	440.215
	18701	Pigf	phosphatidylinositol glycan anchor biosynthesis, class F	452.7625	843.91	435.2815	357.71
	18733	Lilrb3	leukocyte immunoglobulin-like receptor, subfamily B (with TM and ITIM domains), member 3	115.4015	198.3225	97.8129	100.1654
	18824	Plp2	proteolipid protein 2	145.557	372.4505	51.5094	104.145
	18938	Ppp1r14b	protein phosphatase 1, regulatory (inhibitor) subunit 14B	957.923	1910.325	760.0025	838.5805
	19073	Srgn	serglycin	747.7745	1852.67	462.61	767.4265
	192166	Sardh	sarcosine dehydrogenase	3728.045	1668.705	3840.155	2991.46
	192653	Ttc36	tetratricopeptide repeat domain 36	8097.855	3175.355	8238.18	6925.3
	19376	Rab34	RAB34, member of RAS oncogene family	278.864	578.003	271.3815	311.563
	20230	Satb1	special AT-rich sequence binding protein 1	24.43485	50.4068	17.1233	25.64855
	20305	Ccl6	chemokine (C-C motif) ligand 6	140.0215	707.462	102.3031	205.3135
	20322	Sord	sorbitol dehydrogenase	3407.97	707.108	4730.065	3006.09
	20502	Slc16a2	solute carrier family 16 (monocarboxylic acid transporters), member 2	1006.387	384.496	1436.585	899.71
	20516	Slc20a2	solute carrier family 20, member 2	227.606	458.1155	281.325	247.1675
	211389	Suox	sulfite oxidase	1383.38	630.947	1488.095	1265.18
	214459	Fnbp1l	formin binding protein 1-like	373.0135	665.672	275.5235	369.6335
	217258	Abca8a	ATP-binding cassette, sub-family A (ABC1), member 8a	1318.915	285.1915	1857.265	1076.343
	230125	Mcart1	mitochondrial carrier triple repeat 1	848.633	2154.69	727.7265	1159.535
	230514	Leprot	leptin receptor overlapping transcript	357.5655	743.203	354.4405	385.5685
	230737	Gnl2	guanine nucleotide binding protein-like 2 (nucleolar)	462.302	1131.52	454.893	604.213
	233724	Tmem41b	transmembrane protein 41B	83.2496	187.2005	111.6023	77.5362
	237831	Slc13a5	solute carrier family 13 (sodium-dependent citrate transporter), member 5	294.4715	958.397	280.0065	383.14
	23957	Nr0b2	nuclear receptor subfamily 0, group B, member 2	1635.29	408.944	1286.037	955.877
	240505	Cdc42bpg	CDC42 binding protein kinase gamma (DMPK-like)	176.396	368.2295	147.468	169.13
	244745	Dpy19l1	dpy-19-like 1 (C. elegans)	1211.855	2606.74	1063.935	1245.99
	252903	Ap1s3	adaptor-related protein complex AP-1, sigma 3	77.92255	144.6785	72.1272	71.6742
	26879	B3galnt1	UDP-GalNAc:betaGlcNAc beta 1,3-galactosaminyltransferase, polypeptide 1	91.3092	179.592	89.27915	53.6984
	27053	Asns	asparagine synthetase	43.01205	164.859	19.22665	55.17675
	27413	Abcb11	ATP-binding cassette, sub-family B (MDR/TAP), member 11	3651.045	827.294	2613.905	2478.615
	28199	Dcaf11	DDB1 and CUL4 associated factor 11	2471.815	1022.823	2879.845	2671.465
	29811	Ndrg2	N-myc downstream regulated gene 2	3932.09	1567.55	4629.33	3698.795
	320078	Olfml2b	olfactomedin-like 2B	129.1	272.8425	129.2175	138.9185
	320117	C730049O14Rik	RIKEN cDNA C730049O14 gene	2415.08	492.413	1304.835	1181.37
	331535	Serpina7	serine (or cysteine) peptidase inhibitor, clade A (alpha-1 antiproteinase, antitrypsin), member 7	1549.42	3545.34	829.013	1068.855
	402735	B230114P17Rik	RIKEN cDNA B230114P17 gene	329.237	184.6605	410.0635	321.777
	433855	AI506816	expressed sequence AI506816	219.2925	464.85	176.4155	209.2045
	51797	Ctps	cytidine 5'-triphosphate synthase	669.936	1448.115	407.972	731.8615
	53605	Nap1l1	nucleosome assembly protein 1-like 1	485.8305	1133.913	323.234	320.8455
	55989	Nop58	NOP58 ribonucleoprotein homolog (yeast)	206.3545	543.769	176.363	253.9155
	56095	Ftsj3	FtsJ homolog 3 (E. coli)	272.575	541.887	236.625	292.2015
	56360	Acot9	acyl-CoA thioesterase 9	270.805	626.3655	181.9675	309.863
	56449	Csda	cold shock domain protein A	358.4415	853.6365	449.7015	422.933
	56744	Pf4	platelet factor 4	407.633	783.563	330.204	320.5565
	64339	Fndc4	fibronectin type III domain containing 4	134.4465	272.2535	129.3465	149.182
	64424	Polr1e	polymerase (RNA) I polypeptide E	130.689	289.637	148.5565	140.774
	66143	Eef1e1	eukaryotic translation elongation factor 1 epsilon 1	398.7865	913.49	613.694	529.3895
	66645	Pspc1	paraspeckle protein 1	131.0015	538.2855	139.511	189.869
	66972	Slc25a23	solute carrier family 25 (mitochondrial carrier; phosphate carrier), member 23	1334.835	413.275	1504.786	826.0205
	66979	Pole4	polymerase (DNA-directed), epsilon 4 (p12 subunit)	279.1835	604.9475	364.9715	306.65
	67426	Cabc1	chaperone, ABC1 activity of bc1 complex like (S. pombe)	3112.26	456.86	2317.205	1541.54
	67429	Nudcd1	NudC domain containing 1	375.931	705.31	430.5815	406.89
	67469	Abhd5	abhydrolase domain containing 5	514.549	914.2275	822.8885	478.606
	67770	5830433M19Rik	RIKEN cDNA 5830433M19 gene	107.7685	258.273	117.4309	113.62
	67877	Nat5	N-acetyltransferase 5 (ARD1 homolog, S. cerevisiae)	684.132	1410.405	647.0555	830.501
	68444	Cyp2d13	cytochrome P450, family 2, subfamily d, polypeptide 13	722.6445	328.5755	1107.842	726.277
	68567	Cgref1	cell growth regulator with EF hand domain 1	116.276	437.344	109.57	200.68
	70152	Mettl7a1	methyltransferase like 7A1	2925.2	975.646	4237.045	2982.15

Location	Entrez ID	Symbol	Gene Name	rAd.β-gal before	rAd.β-gal after	rAd.A20 before	rAd.A20 after
	70434	2610201A13Rik	RIKEN cDNA 2610201A13 gene	56.78465	191.866	53.80445	69.6253
	71706	Slc46a3	solute carrier family 46, member 3	574.408	229.2645	993.287	592.407
	71986	Ddx28	DEAD (Asp-Glu-Ala-Asp) box polypeptide 28	411.6565	1152.05	309.207	505.009
	72535	Aldh1b1	aldehyde dehydrogenase 1 family, member B1	818.783	322.685	581.1025	553.719
	72844	Kctd17	potassium channel tetramerisation domain containing 17	316.4425	717.402	215.937	321.079
	74318	Hopx	HOP homeobox	1360.64	2858.96	1323.45	1377.22
	74840	Manf	mesencephalic astrocyte-derived neurotrophic factor	2678.635	5294.51	3176.295	2835.315
	75746	Morc4	microrchidia 4	120.5945	377.173	87.8711	141.8335
	76737	Creld2	cysteine-rich with EGF-like domains 2	1308.59	2836.13	1506.725	933.665
	76889	Adck4	aarF domain containing kinase 4	456.1	2030.295	416.629	539.786
	77125	Il33	interleukin 33	99.5805	315.183	87.2039	96.46235
	77670	9130208E07Rik	RIKEN cDNA 9130208E07 gene	239.981	477.186	207.114	241.918
	78752	Csgalnact2	chondroitin sulfate N-acetylgalactosaminyltransferase 2	158.2725	423.068	254.138	222.1425
	81703	Jdp2	Jun dimerization protein 2	113.4566	242.622	107.6648	132.803
	83379	Klb	klotho beta	418.7465	184.9945	494.059	518.824
	83702	Akr1c6	aldo-keto reductase family 1, member C6	7617.08	2989.265	10222.96	7566.315
	83961	Nrg4	neuregulin 4	150.225	855.5895	162.504	301.7825
**q15**	105833	Ccdc65	coiled-coil domain containing 65	96.80635	102.3582	288.537	133.2205
	105887	Ugt3a1	UDP glycosyltransferases 3 family, polypeptide A1	531.3695	329.5175	1584.12	586.635
	107770	Tm6sf2	transmembrane 6 superfamily member 2	1469.595	1378.56	662.1735	1233.97
	112417	Ugt2b37	UDP glucuronosyltransferase 2 family, polypeptide B37	211.397	187.5855	617.4065	245.443
	11465	Actg1	actin, gamma, cytoplasmic 1	7254.705	8257.32	3182.66	7311.165
	11656	Alas2	aminolevulinic acid synthase 2, erythroid	1819.855	1206.202	3694.495	1385.79
	11910	Atf3	activating transcription factor 3	488.6985	324.5615	175.3805	536.2245
	12227	Btg2	B-cell translocation gene 2, anti-proliferative	1193.815	1590.085	297.9715	1159.11
	13096	Cyp2c37	cytochrome P450, family 2. subfamily c, polypeptide 37	161.9155	107.2965	349.9035	127.743
	13107	Cyp2f2	cytochrome P450, family 2, subfamily f, polypeptide 2	3990.695	2748.845	8753.58	3811.89
	13170	Dbp	D site albumin promoter binding protein	96.95215	313.489	2422.29	336.5855
	14104	Fasn	fatty acid synthase	3538.315	1845.895	1650.92	849.395
	14228	Fkbp4	FK506 binding protein 4	227.243	193.6355	808.099	342.72
	16180	Il1rap	interleukin 1 receptor accessory protein	836.981	667.74	2199.275	857.1715
	16803	Lbp	lipopolysaccharide binding protein	3247.285	3974.365	1436.025	3575.515
	17219	Mcm6	minichromosome maintenance deficient 6 (MIS5 homolog, S. pombe) (S. cerevisiae)	2137.42	2725.605	899.256	2483.705
	18142	Npas1	neuronal PAS domain protein 1	89.0443	79.57335	210.946	88.8011
	19255	Ptpn2	protein tyrosine phosphatase, non-receptor type 2	1094.907	1432.065	513.495	947.651
	20210	Saa3	serum amyloid A 3	5431.755	7319.92	421.0275	3085.21
	209488	Hsh2d	hematopoietic SH2 domain containing	191.57	178.56	91.05935	181.0825
	215929	AI317395	expressed sequence AI317395	228.4745	179.42	630.448	211.419
	217410	Trib2	tribbles homolog 2 (Drosophila)	6.35326	8.54448	39.51045	12.25635
	217845	Ifi27l2b	interferon, alpha-inducible protein 27 like 2B	684.728	677.019	324.0315	575.661
	218103	Slc17a2	solute carrier family 17 (sodium phosphate), member 2	842.25	573.412	2152.94	1040.867
	21835	Thrsp	thyroid hormone responsive SPOT14 homolog (Rattus)	1693.074	889.2525	7225.68	879.156
	22151	Tubb2a	tubulin, beta 2A	3541.125	5086.72	1131.81	5382.695
	22352	Vim	vimentin	1708.07	1601.48	622.8905	1713.065
	233067	Lrfn3	leucine rich repeat and fibronectin type III domain containing 3	551.426	376.5615	220.567	433.65
	234757	BC024137	cDNA sequence BC024137	182.258	115.2445	869.9735	138.544
	236794	Slc9a6	solute carrier family 9 (sodium/hydrogen exchanger), member 6	244.5095	308.676	723.9265	376.017
	245867	Pcmtd2	protein-L-isoaspartate (D-aspartate) O-methyltransferase domain containing 2	174.4275	143.8255	407.0445	159.738
	26424	Nr5a2	nuclear receptor subfamily 5, group A, member 2	394.7785	385.373	1297.955	709.3265
	268534	Sntg2	syntrophin, gamma 2	114.788	68.77285	198.028	98.8665
	28248	Slco1a1	solute carrier organic anion transporter family, member 1a1	514.613	378.842	3307.48	805.933
	29869	Ulk2	Unc-51 like kinase 2 (C. elegans)	296.7065	273.875	637.236	351.757
	321018	Serpina4-ps1	serine (or cysteine) peptidase inhibitor, clade A, member 4, pseudogene 1	178.297	704.241	1567.435	377.5875
	338365	Slc41a2	solute carrier family 41, member 2	743.2475	1256.88	284.47	1171.04
	353187	Nr1d2	nuclear receptor subfamily 1, group D, member 2	386.313	785.768	2036.68	997.234
	52163	Camk1	calcium/calmodulin-dependent protein kinase I	388.3465	398.6165	961.5175	509.771
	55927	Hes6	hairy and enhancer of split 6 (Drosophila)	547.621	335.8255	1418.45	429.8365
	57429	Sult5a1	sulfotransferase family 5A, member 1	233.572	220.9905	640.6365	231.3695
	58800	Trpm7	transient receptor potential cation channel, subfamily M, member 7	350.6335	412.3015	1293.64	491.6525
	619326	9130409I23Rik	RIKEN cDNA 9130409I23 gene	274.0765	119.0015	749.0635	106.9755
	66129	1110018J18Rik	RIKEN cDNA 1110018J18 gene	311.9605	446.0175	775.665	445.34
	67528	Nudt7	nudix (nucleoside diphosphate linked moiety X)-type motif 7	1517.899	1180.33	7430.225	1501.615
	67876	Coq10b	coenzyme Q10 homolog B (S. cerevisiae)	102.4667	184.813	414.6515	161.2395
	68337	Crip2	cysteine rich protein 2	2278.015	2081.9	1163.72	2191.66
	69981	Tmem30a	transmembrane protein 30A	151.7775	148.861	400.6285	168.6485
	70024	Mcm10	minichromosome maintenance deficient 10 (S. cerevisiae)	1018.445	760.269	2846.62	850.9475
	71773	Ugt2b1	UDP glucuronosyltransferase 2 family, polypeptide B1	2768.625	1925.24	6525.745	2761.35
	72094	Ugt2a3	UDP glucuronosyltransferase 2 family, polypeptide A3	1443.305	1361.895	3006.135	1625.795
	72972	Gcap14	granule cell antiserum positive 14	1079.517	2180.825	430.6505	1354.1
	75600	Calml4	calmodulin-like 4	172.2285	179.0535	79.0794	171.5775
	76161	6330527O06Rik	RIKEN cDNA 6330527O06 gene	33.42015	27.59135	77.95525	38.38225
	76971	2810007J24Rik	RIKEN cDNA 2810007J24 gene	1475.955	671.4385	6313.62	1395.72
	77619	Prelid2	PRELI domain containing 2	808.1055	1378.265	280.016	769.6845

The genes were identified in any of following comparison: i) βgal after vs. before resection, ii) A20 after vs. before resection, iii) A20 before resection vs. βgal before resection, or iv) A20 after resection vs. βgal after resection. The gene list was divided in 15 sections on the basis of the Venn diagram shown in Fig. S2. The table depicts the average expression level of the gene in A20 before and after resection as well as βgal before and after resection groups.
