# Supplementary material for: A20 Modulates Lipid Metabolism and Energy Production to Promote Liver Regeneration
Source: PLoS One. 2011 Mar 17;6(3):e17715. doi: 10.1371/journal.pone.0017715 (PMC3060102; doi:10.1371/journal.pone.0017715)
Supplement: Table S3 — Gene Ontology analysis of SOM patterns. This analysis was performed using the Database for Annotation, Visualization and Integrated Discovery (DAVID ) v6.7 and gene ontology categories with multiple test (Holm–Bonferroni method) corrected P value <0.05 were considered significant. (PDF) [file pone.0017715.s008.pdf]

Supplementary Table 3: Gene Ontology analysis of SOM patterns

Cluster	GO category	PValue	Bonferroni	Benjamini	FDR
A	GO:0006953~acute-phase response	1.58E-06	1.24E-04	1.24E-04	0.00167495
	GO:0002526~acute inflammatory response	3.25E-05	0.00253446	0.00126803	0.03440818
	GO:0006952~defense response	3.20E-04	0.02465078	0.00828538	0.33791341
	GO:0006954~inflammatory response	6.70E-04	0.05093971	0.01298569	0.70651655
	GO:0009611~response to wounding	0.00233891	0.16693894	0.03587051	2.44652206
	GO:0033209~tumor necrosis factor-mediated signaling pathway	0.00323458	0.2233038	0.04124304	3.36896172
B	GO:0055085~transmembrane transport	1.32E-04	0.11524319	0.11524319	0.20665382
	GO:0055066~di-, tri-valent inorganic cation homeostasis	0.00220131	0.87005715	0.63952413	3.38895782
	GO:0006461~protein complex assembly	0.00296841	0.93625292	0.60052762	4.54443584
	GO:0070271~protein complex biogenesis	0.00296841	0.93625292	0.60052762	4.54443584
	GO:0032101~regulation of response to external stimulus	0.00394095	0.97417802	0.59913568	5.99078609
	GO:0055065~metal ion homeostasis	0.00436589	0.98260657	0.55528985	6.61628447
	GO:0065003~macromolecular complex assembly	0.00520051	0.99199942	0.55278102	7.83351037
	GO:0055080~cation homeostasis	0.00590572	0.99585107	0.54322198	8.85038871
	GO:0045444~fat cell differentiation	0.00654814	0.99771985	0.53253904	9.76759295
	GO:0048878~chemical homeostasis	0.00782144	0.99930467	0.55420815	11.5600474
	GO:0043933~macromolecular complex subunit organization	0.00804847	0.99943746	0.52683167	11.8761213
	GO:0050729~positive regulation of inflammatory response	0.00976166	0.9998865	0.56211168	14.2273517
	GO:0030005~cellular di-, tri-valent inorganic cation homeostasis	0.00989459	0.99989977	0.53575142	14.4073143
C	GO:0002274~myeloid leukocyte activation	0.00343368	0.81903955	0.81903955	4.82849702
	GO:0007049~cell cycle	0.01708181	0.99980896	0.98617813	21.956233
	GO:0055114~oxidation reduction	0.02469342	0.99999599	0.98411267	30.2152692
	GO:0042116~macrophage activation	0.02475031	0.9999961	0.95557363	30.2738096
	GO:0051301~cell division	0.03266343	0.99999993	0.963152	37.9863551
	GO:0001817~regulation of cytokine production	0.04709859	1	0.98161458	50.0497802
D	GO:0022613~ribonucleoprotein complex biogenesis	2.30E-05	0.03904446	0.03904446	0.03894293
	GO:0042254~ribosome biogenesis	7.15E-05	0.11623301	0.05991118	0.1207692
	GO:0006259~DNA metabolic process	4.73E-04	0.5581783	0.23836128	0.79568719
	GO:0006396~RNA processing	7.81E-04	0.74058583	0.28632838	1.31096113
	GO:0046907~intracellular transport	0.00153269	0.92938492	0.41145525	2.55886437
	GO:0030036~actin cytoskeleton organization	0.0019722	0.96700275	0.4336569	3.28120744
	GO:0007010~cytoskeleton organization	0.00209184	0.97317661	0.40364901	3.47695212
	GO:0031532~actin cytoskeleton reorganization	0.00249615	0.98668322	0.41715927	4.13571911
	GO:0009967~positive regulation of signal transduction	0.00273007	0.99112053	0.40837975	4.51493643
	GO:0030029~actin filament-based process	0.00325904	0.99645004	0.43111776	5.36725376
	GO:0006974~response to DNA damage stimulus	0.00366657	0.99824882	0.43844331	6.01900224
	GO:0010647~positive regulation of cell communication	0.00555722	0.99993425	0.55177993	8.98783302
	GO:0007049~cell cycle	0.00586708	0.99996163	0.54258641	9.46590707
	GO:0015931~nucleobase, nucleoside, nucleotide and nucleic acid	0.00823304	0.99999938	0.63954863	13.0390865
E	GO:0055114~oxidation reduction	1.03E-09	9.38E-07	9.38E-07	1.61E-06
	GO:0008202~steroid metabolic process	1.43E-05	0.01297239	0.00650737	0.02235257
	GO:0006694~steroid biosynthetic process	4.36E-04	0.32811686	0.12414695	0.67853137
F	GO:0000097~sulfur amino acid biosynthetic process	0.0153517	0.85086494	0.85086494	16.33818
	GO:0000096~sulfur amino acid metabolic process	0.02510419	0.95616115	0.79062272	25.4098969
	GO:0044271~nitrogen compound biosynthetic process	0.04272496	0.9953493	0.83308088	39.5575984
	GO:0044272~sulfur compound biosynthetic process	0.0443393	0.99622116	0.75206403	40.7225295
	GO:0008652~cellular amino acid biosynthetic process	0.04751051	0.99748925	0.69803212	42.9514196
G	GO:0006955~immune response	2.03E-16	3.74E-13	3.74E-13	3.77E-13
	GO:0006952~defense response	9.07E-08	1.53E-04	7.63E-05	1.53E-04
	GO:0042110~T cell activation	4.35E-07	7.32E-04	2.44E-04	7.33E-04
	GO:0050865~regulation of cell activation	1.74E-06	0.00292082	7.31E-04	0.00292794
	GO:0001775~cell activation	3.01E-06	0.00505593	0.00101324	0.00507364

Cluster	GO category	PValue	Bonferroni	Benjamini	FDR
	GO:0030097~hemopoiesis	3.90E-06	0.00654528	0.00109387	0.00657308
	GO:0051249~regulation of lymphocyte activation	4.38E-06	0.00734654	0.00105283	0.00738069
	GO:0045619~regulation of lymphocyte differentiation	4.39E-06	0.00736451	9.24E-04	0.0073988
	GO:0002694~regulation of leukocyte activation	8.76E-06	0.01463978	0.00163733	0.01476151
	GO:0050863~regulation of T cell activation	1.10E-05	0.01839036	0.00185444	0.01857821
	GO:0045582~positive regulation of T cell differentiation	1.16E-05	0.01930859	0.00177092	0.01951483
	GO:0045580~regulation of T cell differentiation	1.21E-05	0.02020353	0.00169942	0.02042852
	GO:0019882~antigen processing and presentation	1.24E-05	0.02069267	0.00160715	0.02092827
	GO:0045321~leukocyte activation	1.54E-05	0.02551378	0.00184436	0.02586713
	GO:0046649~lymphocyte activation	1.60E-05	0.0265876	0.00179488	0.02697048
**H**	GO:0010565~regulation of cellular ketone metabolic process	6.79E-04	0.20339696	0.20339696	0.91809586
	GO:0042304~regulation of fatty acid biosynthetic process	0.01513572	0.99395927	0.92227784	18.716886
	GO:0010907~positive regulation of glucose metabolic process	0.01513572	0.99395927	0.92227784	18.716886
	GO:0010676~positive regulation of cellular carbohydrate metabo	0.01680387	0.99657663	0.84928686	20.5677827
	GO:0045913~positive regulation of carbohydrate metabolic proc	0.01680387	0.99657663	0.84928686	20.5677827
	GO:0043467~regulation of generation of precursor metabolites a	0.02179214	0.99937708	0.84201759	25.8721648
	GO:0010906~regulation of glucose metabolic process	0.02344951	0.99964704	0.79603993	27.5606209
	GO:0006109~regulation of carbohydrate metabolic process	0.02510419	0.99980002	0.75817581	29.2107382
	GO:0010675~regulation of cellular carbohydrate metabolic proce	0.02510419	0.99980002	0.75817581	29.2107382
	GO:0019217~regulation of fatty acid metabolic process	0.03169614	0.9999794	0.78592885	35.4448849
	GO:0046890~regulation of lipid biosynthetic process	0.0366121	0.99999626	0.79026318	39.7585572
	GO:0008624~induction of apoptosis by extracellular signals	0.04150419	0.99999932	0.79358223	43.7848438
**I**	GO:0055114~oxidation reduction	1.49E-15	1.56E-12	1.56E-12	2.30E-12
	GO:0008202~steroid metabolic process	2.62E-06	0.00283067	0.00141634	0.00418754
	GO:0006631~fatty acid metabolic process	8.64E-06	0.00928285	0.00310391	0.01377649
	GO:0055092~sterol homeostasis	9.28E-05	0.09534576	0.02473945	0.14791792
	GO:0042632~cholesterol homeostasis	9.28E-05	0.09534576	0.02473945	0.14791792
	GO:0055088~lipid homeostasis	2.72E-04	0.25428201	0.05699299	0.4325084
	GO:0046395~carboxylic acid catabolic process	9.57E-04	0.64459466	0.15837107	1.51662579
	GO:0016054~organic acid catabolic process	9.57E-04	0.64459466	0.15837107	1.51662579
	GO:0019318~hexose metabolic process	0.00104055	0.6751444	0.14838857	1.64730082
	GO:0008610~lipid biosynthetic process	0.00141742	0.78387512	0.17426994	2.23763383
	GO:0019319~hexose biosynthetic process	0.00157794	0.81832164	0.17262889	2.48805712
	GO:0016053~organic acid biosynthetic process	0.00207659	0.8940781	0.20108868	3.26218421
	GO:0046394~carboxylic acid biosynthetic process	0.00207659	0.8940781	0.20108868	3.26218421
	GO:0005996~monosaccharide metabolic process	0.00210133	0.89687619	0.18659777	3.30043594
	GO:0046364~monosaccharide biosynthetic process	0.00296055	0.95932473	0.23420793	4.62031798
**J**	GO:0048193~Golgi vesicle transport	0.00188304	0.25755046	0.25755046	2.24540445
	GO:0006888~ER to Golgi vesicle-mediated transport	0.03568976	0.99679188	0.9433597	35.459986
	GO:0006886~intracellular protein transport	0.04574646	0.99938782	0.91508997	43.1186003
**K**	GO:0007049~cell cycle	0.00159644	0.24512046	0.24512046	1.94149541
	GO:0022402~cell cycle process	0.00231148	0.33454984	0.18424871	2.79981588
	GO:0000910~cytokinesis	0.03902641	0.99909382	0.90323048	38.6452764
**L**	GO:0007067~mitosis	0.0230449	0.98309343	0.98309343	24.8606348
	GO:0000280~nuclear division	0.0230449	0.98309343	0.98309343	24.8606348
	GO:0000087~M phase of mitotic cell cycle	0.02395792	0.98564527	0.88018877	25.7170027
	GO:0048285~organelle fission	0.02465247	0.9873266	0.76685232	26.362446
	GO:0000278~mitotic cell cycle	0.03657942	0.99852841	0.80413964	36.6726958
	GO:0007422~peripheral nervous system development	0.03689879	0.99861137	0.73176376	36.9295823
	GO:0051301~cell division	0.04724974	0.99979043	0.75628081	44.7549182
	GO:0000279~M phase	0.04785623	0.99981253	0.70653027	45.1845071
	GO:0010810~regulation of cell-substrate adhesion	0.04891091	0.99984558	0.66612124	45.9242634
**P**	GO:0055114~oxidation reduction	5.99E-05	0.02583107	0.02583107	0.08456959

Cluster	GO category	PValue	Bonferroni	Benjamini	FDR
	GO:0006732~coenzyme metabolic process	0.0068268	0.94989042	0.7761483	9.22412616
	GO:0051186~cofactor metabolic process	0.01315056	0.99692643	0.85460565	17.0570745
	GO:0016054~organic acid catabolic process	0.02042539	0.99987883	0.89508218	25.2893961
	GO:0046395~carboxylic acid catabolic process	0.02042539	0.99987883	0.89508218	25.2893961
	GO:0009225~nucleotide-sugar metabolic process	0.02690746	0.99999334	0.90781251	31.9783456

The analysis was performed using the Database for Annotation, Visualization and Integrated Discovery (DAVID) v6.7 and gene ontology categories with multiple test (Holm–Bonferroni method) corrected P value <0.05 were considered significant.
